# Mortality rates among patients successfully treated for hepatitis C in the era of interferon-free antivirals: population based cohort study

**DOI:** 10.1136/bmj-2022-074001

**Published:** 2023-08-02

**Authors:** Victoria Hamill, Stanley Wong, Jennifer Benselin, Mel Krajden, Peter C Hayes, David Mutimer, Amanda Yu, John F Dillon, William Gelson, Hector A Velásquez García, Alan Yeung, Philip Johnson, Stephen T Barclay, Maria Alvarez, Hidenori Toyoda, Kosh Agarwal, Andrew Fraser, Sofia Bartlett, Mark Aldersley, Andy Bathgate, Mawuena Binka, Paul Richardson, Joanne R Morling, Stephen D Ryder, Douglas MacDonald, Sharon Hutchinson, Eleanor Barnes, Indra Neil Guha, William L Irving, Naveed Z Janjua, Hamish Innes

**Affiliations:** 1 School of Health and Life Sciences, Glasgow Caledonian University, Glasgow, UK; 2 Public Health Scotland, Glasgow, UK; 3 British Columbia Centre for Disease Control, Vancouver, British Columbia, Canada; 4 NIHR Nottingham Biomedical Research Centre, Nottingham University Hospitals NHS Trust and the University of Nottingham, Nottingham, UK; 5 Nottingham Digestive Diseases Centre, School of Medicine, University of Nottingham, UK; 6 Department of Pathology and Laboratory Medicine, The University of British Columbia, Vancouver, British Columbia, Canada; 7 Royal Infirmary of Edinburgh, Edinburgh, UK; 8 Liver and Hepatology Unit, Queen Elizabeth Hospital, University Hospitals Birmingham NHS Foundation Trust, Birmingham, UK; 9 Division of Molecular and Clinical Medicine, School of Medicine, University of Dundee, UK; 10 Cambridge Liver Unit, Cambridge University Hospitals NHS Foundation Trust, Cambridge, UK; 11 School of Population and Public Health, University of British Columbia, Vancouver, British Columbia, Canada; 12 Department of Molecular and Clinical Cancer Medicine, University of Liverpool, Liverpool, UK; 13 Glasgow Royal Infirmary, Glasgow, UK; 14 Department of Gastroenterology, Ogaki Municipal Hospital, Ogaki, Japan; 15 Institute of Liver Studies, King’s College Hospital NHS Foundation Trust, London, UK; 16 Aberdeen Royal Infirmary, Aberdeen, UK; 17 Queen Elizabeth University Hospital, Glasgow, UK; 18 Leeds Liver Unit, St James’s University Hospital, Leeds, UK; 19 Royal Liverpool and Broadgreen University Hospitals NHS Trust, Liverpool, UK; 20 Lifespan and Population Health, University of Nottingham, Nottingham, UK; 21 Gastroenterology and Hepatology, Royal Free London NHS Foundation Trust, London, UK; 22 Nuffield Department of Medicine and the Oxford NIHR Biomedical Research Centre, University of Oxford, Oxford, UK; 23 Centre for Health Evaluation and Outcome Sciences, St Paul’s Hospital Vancouver, British Columbia, Canada; * Joint first authors

## Abstract

**Objectives:**

To quantify mortality rates for patients successfully treated for hepatitis C in the era of interferon-free, direct acting antivirals and compare these rates with those of the general population.

**Design:**

Population based cohort study.

**Setting:**

British Columbia, Scotland, and England (England cohort consists of patients with cirrhosis only).

**Participants:**

21 790 people who were successfully treated for hepatitis C in the era of interferon-free antivirals (2014-19). Participants were divided into three liver disease severity groups: people without cirrhosis (pre-cirrhosis), those with compensated cirrhosis, and those with end stage liver disease. Follow-up started 12 weeks after antiviral treatment completion and ended on date of death or 31 December 2019.

**Main outcome measures:**

Crude and age-sex standardised mortality rates, and standardised mortality ratio comparing the number of deaths with that of the general population, adjusting for age, sex, and year. Poisson regression was used to identify factors associated with all cause mortality rates.

**Results:**

1572 (7%) participants died during follow-up. The leading causes of death were drug related mortality (n=383, 24%), liver failure (n=286, 18%), and liver cancer (n=250, 16%). Crude all cause mortality rates (deaths per 1000 person years) were 31.4 (95% confidence interval 29.3 to 33.7), 22.7 (20.7 to 25.0), and 39.6 (35.4 to 44.3) for cohorts from British Columbia, Scotland, and England, respectively. All cause mortality was considerably higher than the rate for the general population across all disease severity groups and settings; for example, all cause mortality was three times higher among people without cirrhosis in British Columbia (standardised mortality ratio 2.96, 95% confidence interval 2.71 to 3.23; P<0.001) and more than 10 times higher for patients with end stage liver disease in British Columbia (13.61, 11.94 to 15.49; P<0.001). In regression analyses, older age, recent substance misuse, alcohol misuse, and comorbidities were associated with higher mortality rates.

**Conclusion:**

Mortality rates among people successfully treated for hepatitis C in the era of interferon-free, direct acting antivirals are high compared with the general population. Drug and liver related causes of death were the main drivers of excess mortality. These findings highlight the need for continued support and follow-up after successful treatment for hepatitis C to maximise the impact of direct acting antivirals.

## Introduction

Interferon-free, direct acting antiviral regimens have transformed the clinical management and epidemiology of hepatitis C virus (HCV) infection.^1^ Treatment used to be long, arduous, and ineffective.^2^ ^3^ ^4^ ^5^ Interferon-free, direct acting antiviral regimens are short, tolerable, and lead to a virological cure in >95% of patients.^6^ Therefore, the number of people who have been successfully treated for HCV has increased dramatically since these new treatments became available in 2014.^7^ The rise has been most pronounced in patients with cirrhosis in whom previous regimens were least effective or were contraindicated.^4^ ^5^ For example, in Scotland, the number of people with cirrhosis who have received successful HCV treatment increased sixfold between 2014 and 2019 (from approximately 300 to 1800).^7^ This upward trajectory will continue for the foreseeable future as countries strive to eliminate HCV in alignment with the World Health Organization global strategy for viral hepatitis.^9^ ^10^ ^11^

However, it is important to understand the overall prognosis for people who have been successfully treated for HCV. Most observational studies have focused on quantifying the relative benefits of an HCV cure. These benefits include a lower mortality risk compared with untreated patients with chronic HCV infection and those in whom treatment has failed.^12^ ^13^ ^14^ Yet, the prognosis for people who have been successfully treated for HCV remains debatable, particularly in the era of interferon-free, direct acting antiviral regimens. Several studies suggest that people with cirrhosis who have been successfully treated for HCV have low mortality rates, which are comparable to the general population after adjustment for age, sex, and calendar year.^15^ ^16^ ^17^ ^18^ However, in our view, data from larger and more representative cohorts that encompass patients with a broad spectrum of liver disease severity are needed to form a reliable picture of prognosis for people who have been successfully treated for HCV. Therefore, we obtained and analysed data from three population based cohorts consisting of people who have been successfully treated for HCV in the era of interferon-free antivirals (from 2014 onwards). Our goal was to quantify mortality rates and assess how these rates compare with those of the general population.

## Methods

### Data cohorts

*BC hepatitis testers cohort (British Columbia)*—This cohort includes people tested for HCV in British Columbia since 1990.^19^ ^20^ Data for people who were tested are linked routinely to information on outpatient and emergency department visits (through the Medical Services Plan and the National Ambulatory Care Reporting System); hospital admissions (through the Discharge Abstracts Dataset); cancers (through the BC Cancer Registry); and prescriptions (through PharmaNet). Prescription data cover all prescriptions for HCV antiviral treatment dispensed in British Columbia. Linked mortality data for this cohort were obtained through record linkage with the BC Vital Statistics Agency Death Registry.^19^ ^20^ The creation of this cohort and integration of data were performed under the auspices of the BC Centre for Disease Control’s public health mandate, which was reviewed and approved by the Behavioural Research Ethics Board at the University of British Columbia (H14-01649).

*Scottish HCV clinical database (Scotland)*—This database contains clinical follow-up data for patients receiving HCV treatment in Scotland (in hospital, prisons, or community settings). Data fields available include start and end date of HCV treatment, treatment regimen, response to treatment, and date of diagnosis of cirrhosis or hepatocellular carcinoma.^21^ ^22^ Mortality data for the HCV patient population—specifically date and cause of death—were obtained by record linkage to the Scottish mortality register. Approval to link these registries and perform data analysis was granted by the Privacy Public Benefit Panel for Health and Social Care in NHS Scotland (application No 1516-0457).

*HCV Research UK (England; HCVRUK)*—This cohort comprises >10 000 patients with HCV recruited between 2012 and 2016 from more than 50 UK liver centres.^23^ Participant characteristics included clinical, epidemiological, virological, and treatment related factors determined through clinical notes or direct self-report at study enrolment. The study was approved by the East Midlands Research Ethics Committee (application reference 11/EM/0314). Informed consent was obtained from all participants.

Recently, HCVRUK participants from England with a cirrhosis diagnosis have been linked to national health registries held by NHS Digital (application No NIC-72626). These registries include hospital episodes statistics data (eg, admitted patient care database, diagnostic imaging dataset, and outpatient hospital admissions), mortality registrations, and the National Cancer Registration and Analysis Service.^24^ ^25^ ^26^ HCVRUK participants without cirrhosis have not yet been linked to these data registries, and so were not included in this study.

### Data sources

Data for the number of deaths in the general population for England, Scotland, and British Columbia were provided by the Office for National Statistics, Public Health Scotland, and the BC Vital Statistics Agency, respectively. For each setting or country, deaths were categorised by age group, sex, year of death, and underlying cause of death. These data were then merged with mid-year population estimates and cause specific mortality rates were derived (tables S1-S8).

### Successfully treated HCV

The optimal outcome of HCV treatment is a sustained viral response (SVR), defined as remaining HCV RNA negative for at least 10-12 weeks after treatment completion.^27^ SVR is a robust marker of permanent viral clearance and is considered equivalent to an HCV cure.^28^ We therefore use the terms successfully treated HCV, HCV cure, and SVR interchangeably hereafter. In Scotland, SVR status is recorded for each antiviral treatment episode through the national HCV clinical database. Similarly, in HCVRUK, SVR status was provided for every treatment episode by examining patients’ medical records. In British Columbia, SVR was established electronically using individual level HCV RNA testing data after treatment. Specifically, an undetectable serum HCV RNA test obtained at ≥10 weeks after treatment completion was defined as an SVR.

### Inclusion and exclusion criteria, and follow-up

All adults successfully treated for HCV between 1 January 2014 and 30 December 2019 from the BC hepatitis testers cohort (British Columbia), HCVRUK (England), and Scottish HCV clinical database (Scotland) were eligible for inclusion in this study. Patients who could not be linked to national demographic databases—a prerequisite for record linkage—were excluded from further analysis. Further details are provided in appendix A. For each patient, follow-up started 12 weeks after antiviral treatment completion and ended on date of death or 31 December 2019. Follow-up was censored at 31 December 2019 to ensure our findings were not influenced by the covid-19 pandemic.

### Liver disease severity and cause specific mortality data

Because mortality rates are likely to vary by liver disease severity at SVR, patients were divided into three distinct disease severity groups: patients without cirrhosis (pre-cirrhosis); patients with compensated cirrhosis who had not had hepatocellular carcinoma before SVR; and patients with end stage liver disease, defined as having had any decompensation episode (ascites, bleeding varices, or hepatic encephalopathy) or hepatocellular carcinoma before SVR. Liver disease severity was inferred using a combination of information extracted from patient medical records or national data registries (table S9).

Seven causes of death were examined: primary liver cancer, liver failure, drug related causes, external causes (referring mainly to accidents, homicides, and suicides), extrahepatic cancer, diseases of the circulatory system, and death from any other cause. The international classification of diseases (ICD) code present in the underlying cause of death field was used to categorise deaths into these mortality categories (table S10).

### Study covariates

Hospital admissions occurring before SVR were used to determine alcohol and substance misuse. Three severity levels were considered: no previous admission, non-recent admission (defined as more than three years before SVR), and recent admission (defined as less than three years before SVR). Table S11 provides the ICD codes used to identify these events.

Similarly, hospital admissions in the five years before SVR were used to determine each patient’s Charlson comorbidity index.^29^ For each patient, a score of 1-6 was assigned for each comorbidity present, with a higher score (also known as weight) denoting greater severity (6=most severe; 1=least severe). The individual scores were added together to give the patient’s overall Charlson comorbidity index. Each single comorbidity was defined using the ICD codes previously described by Quan and colleagues^30^; the weightings assigned to each comorbidity were taken from the 2011 study by Quan and colleagues.^31^ Liver disease was removed from the Charlson comorbidity index algorithm to prevent overlap with other variables in the analyses. Other covariates considered were sex, age at SVR, and year of SVR. No data were missing for these variables.

### Statistical analysis

*Measures of absolute mortality*—Cause specific and all cause mortality rates were calculated and stratified by disease stage. We determined crude mortality rates by dividing the number of deaths by total person years of follow-up. Mortality rates standardised for age at cure and sex were also calculated using the British Columbia cohort as a standard population (table S12). These standardised mortality rates effectively describe the crude mortality rate that would be observed in England or Scotland if their age-sex distribution was identical to the British Columbia cohort. This method enabled comparison of mortality rates between settings after eliminating differences in age and sex. We opted to use British Columbia as the standard population rather than Scotland or England because it was the largest cohort and therefore it had the most precisely defined age-sex distribution. We avoided using a generic standard population (eg, the European 2013 standard population), which assumes an equal gender split that does not reflect the demographic composition of people with HCV. Age groups used in direct standardisation were <20, 20-29, 30-39, 40-44, 45-49, 50-54, 55-59, 60-64, 65-69, 70-74, 75-79, and ≥80 years. All mortality rates indicate the number of deaths per 1000 person years of follow-up.

*Mortality relative to general population*—The standardised mortality ratio was calculated to compare mortality rates with those of the general population. This value represents the number of observed deaths divided by the number of expected deaths. In this analysis, the number of expected deaths refers to how many deaths would have occurred if each cohort had the same age, sex, and calendar year specific mortality rates as the corresponding general population. Specifically, by corresponding general population, we mean the British Columbia general population for patients successfully treated for HCV from the BC hepatitis testers cohort, the Scotland general population for patients successfully treated for HCV from the Scottish HCV clinical database, and the England general population for patients successfully treated for HCV from HCVRUK. Tables S1-S8 show the mortality rates used to determine the number of expected deaths in each cohort. Lexis expansion was performed to account for people progressing through different age groups and calendar year over time (see appendix B).

We calculated cause specific standardised mortality ratios and their associated 95% confidence intervals using Poisson regression (by fitting a null model with number of observed deaths as the outcome and number of expected deaths as an offset). Robust standard errors were used to account for the clustered data structure induced by lexis expansion. Excess mortality was defined as a standardised mortality ratio >1; that is, where the number of observed deaths exceeded the number of expected deaths.

*Cause specific contributions to excess mortality*—We calculated the contribution of each cause of death to excess mortality by dividing the number of excess deaths for that cause by the total number of excess deaths. For example, if there were 100 excess deaths from all cause mortality and 20 excess deaths from liver cancer, then the contribution of liver cancer to the overall excess would be 20% (20/100).

*Factors associated with mortality rate and standardised mortality ratio*—We used Poisson regression to model the all cause mortality rate and the all cause standardised mortality ratio. Our goal was to provide insight into how mortality varies according to individual level factors. Table S13 indicates how models were specified. In particular, to model the standardised mortality ratio, the logarithm of the number of expected deaths was used as an offset term; conversely, to model the mortality rate, the offset was person years of follow-up. Separate models were fitted for each setting and disease stage group. Independent variables considered were year of successful treatment, age, sex, alcohol misuse, substance misuse, and Charlson comorbidity index. We fitted univariable models plus a single multivariable model combining all predictors regardless of statistical significance. Age, Charlson comorbidity index, and year were modelled as continuous variables. We used the multivariable fractional polynomial procedure to identify the best fitting functional form for these variables (linear or nonlinear) at a P<0.01 significance level.^32^

### Sensitivity analyses

We adjusted Scottish standardised mortality ratios for the Scottish multiple index of deprivation score in addition to age, sex, and calendar year. We were unable to perform this adjustment in our British Columbia or England cohorts because equivalent area based deprivation data were not available. Additionally, the inclusion of hepatocellular carcinoma within our end stage liver disease group could contribute to heterogeneous mortality rates between settings; therefore, we also performed a sensitivity analysis which excluded patients with hepatocellular carcinoma from the end stage liver disease group.

### Patient and public involvement

Patients and the public were not directly involved in the design, conduct, or reporting of this specific study because of a combination of funding, time, and training constraints. Nevertheless, the questions posed by this study were shaped by conversations with patients and third sector organisations held over a number of years.

## Results

### Patient characteristics

**Fig 1:**
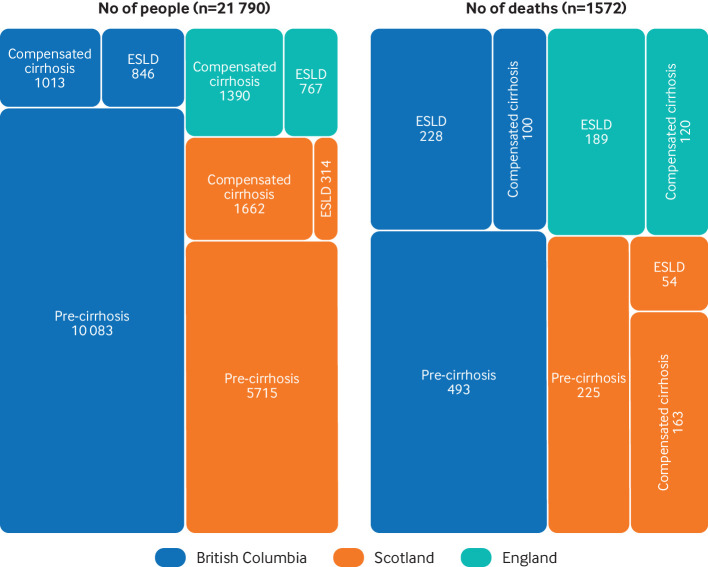
Number of people successfully treated for hepatitis C virus and number of deaths by setting and liver disease severity. ESLD=end stage liver disease

A total of 21 790 people who were successfully treated for HCV were included in the analysis: 11 942 (56.3%) were from British Columbia, 7691 (33.6%) were from Scotland, and 2157 (10.2%) were from England (fig 1). Most people did not have cirrhosis at the time of successful HCV treatment—that is, 74% in Scotland and 84% in British Columbia (fig 1). People with cirrhosis and end stage liver disease were successfully treated for HCV earlier than those without cirrhosis and were also much older (figs S1-S2). Of note, people from Scotland who were successfully treated for HCV were >10 years younger than those from British Columbia. For example, the mean age of people without cirrhosis in Scotland was 44.4 years versus 56.1 years in British Columbia (table 1). Men outnumbered women across all cohorts and disease severity groups (65-75%). The proportion of patients with hospital admission for alcohol misuse before successful HCV treatment increased considerably with disease severity. Between two fifths and one half of participants had a previous hospital admission for substance misuse (table 1 and fig S3).

**Table 1:** Baseline characteristics of people successfully treated for hepatitis C infection by setting and liver disease severity

Characteristic	British Columbia (n=11 942)				Scotland (n=7691)				England (n=2157)	
	Pre-cirrhosis (n=10 083)	Cirrhosis (n=1013)	ESLD (n=846)		Pre-cirrhosis (n=5715)	Cirrhosis (n=1662)	ESLD (n=314)		Cirrhosis (n=1390)	ESLD (n=767)
Year of SVR (median)	2018	2016	2016		2017	2016	2016		2016	2015
Age (years), mean (SD)	56.1 (10.6)	60.3 (7.8)	60.3 (7.7)		44.4 (9.8)	50.6 (8.9)	52.5 (9.5)		55.7 (9.4)	57.1 (8.5)
Sex										
Male	6556 (65)	703 (69)	549 (65)		4095 (72)	1247 (75)	213 (68)		1028 (74)	570 (74)
Female	3527 (35)	310 (31)	297 (35)		1620 (28)	415 (25)	101 (32)		362 (26)	197 (26)
Hospital admission for alcohol misuse										
No	8664 (86)	797 (79)	520 (61)		4377 (77)	1143 (69)	141 (45)		1098 (79)	410 (53)
Yes (not recent)	892 (9)	99 (10)	111 (13)		837 (15)	234 (14)	46 (15)		121 (9)	104 (14)
Yes (recent)	527 (5)	117 (12)	215 (25)		501 (9)	285 (17)	127 (40)		171 (12)	253 (33)
Hospital admission for substance misuse										
No	7437 (74)	819 (81)	632 (75)		3009 (53)	934 (56)	176 (56)		941 (68)	419 (55)
Yes (not recent)	1418 (14)	115 (11)	93 (11)		1432 (25)	394 (24)	61 (19)		141 (10)	71 (9)
Yes (recent)	1228 (12)	79 (8)	121 (14)		1274 (22)	334 (20)	77 (25)		308 (22)	277 (36)
Charlson comorbidity index										
0	8667 (86)	825 (81)	457 (54)		5219 (91)	1448 (87)	205 (65)		1017 (80)	387 (51)
1	629 (6)	69 (7)	101 (12)		329 (6)	138 (8)	37 (12)		200 (14)	136 (18)
2	410 (4)	57 (6)	183 (22)		74 (1)	46 (3)	54 (17)		54 (4)	162 (21)
≥3	377 (4)	62 (6)	105 (12)		93 (2)	30 (2)	18 (6)		29 (2)	82 (11)

Each cell provides the number of participants (corresponding column percentage), unless indicated otherwise. All column percentages have been rounded to the nearest integer. All data relate to baseline time point (date of successful hepatitis C treatment). ESLD=end stage liver disease; SD=standard deviation; SVR=sustained viral response.

### Follow-up and observed mortality

The total duration of follow-up across the cohorts was 53 370 person years. At an individual patient level, the mean follow-up time per patient was 2.2 years (British Columbia), 2.5 years (Scotland), and 3.9 years (England). There were 1572 observed deaths in total. The leading causes of death were drug related mortality (n=383, 24.4%), liver failure (n=286, 18.2%), liver cancer (n=250, 15.9%), and extrahepatic cancer (n=181, 11.5%; table S14).

**Fig 2:**
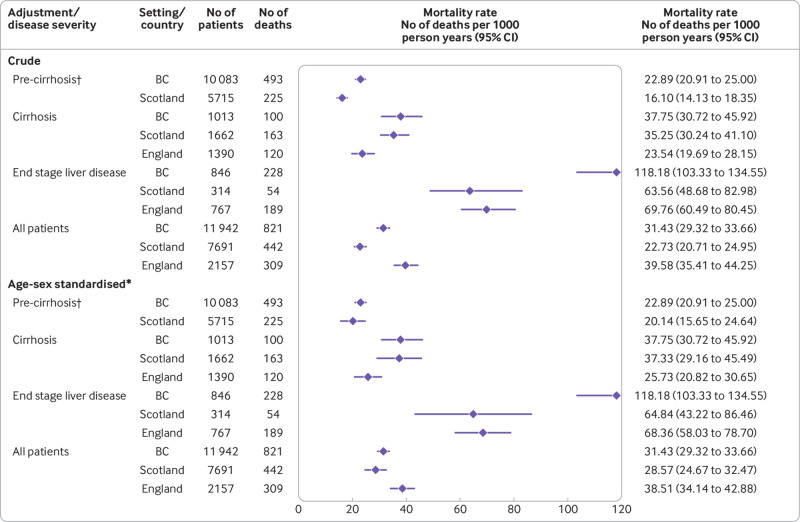
Crude and standardised all cause mortality rates by setting and liver disease severity. *Mortality rates are standardised for age and sex using patients from British Columbia (BC) as standard population; therefore, crude and standardised mortality rates for British Columbia are equal (see table S12 for further details). †Mortality data for people without cirrhosis (pre-cirrhosis) were not available for the England cohort. 95% CI=95% confidence interval

*Mortality rates*—The crude mortality rate (deaths per 1000 person years) was 31.43 (95% confidence interval 29.32 to 33.66), 22.73 (20.71 to 24.95), and 39.58 (35.41 to 44.25) in British Columbia, Scotland, and England, respectively (fig 2). Mortality rates increased considerably with liver disease severity. For example, in Scotland the crude mortality rate was 16.10 (14.13 to 18.35), 35.25 (30.24 to 41.10), and 63.56 (48.68 to 82.98) in people without cirrhosis, those with compensated cirrhosis, and those with end stage liver disease, respectively.

Standardised mortality rates were more comparable between England, Scotland, and British Columbia than the equivalent crude rates (fig 2). However, key differences remained even after standardisation. In particular, the mortality rate for patients with end stage liver disease was considerably higher in British Columbia (118.2 deaths per 1000 person years) than for Scotland (64.8) and England (68.4). This difference persisted in a sensitivity analysis excluding patients with liver cancer at cure (figs S4-S5).

Figures S6-S11 show cause specific mortality. In people without cirrhosis, the mortality rate for drug misuse was considerably higher than for other causes of death (7.0-9.3 deaths per 1000 person years; fig S6). In patients with compensated cirrhosis, the rate of drug related mortality was comparable to people without cirrhosis, but death from liver cancer (5.5-7.2 deaths per 1000 person years) and liver failure (4.1-7.5) were more prominent (fig S7). Conversely, in patients with end stage liver disease, the rate of liver failure (22.4-45.6) and liver cancer mortality (20.3-31.1) far exceeded other causes of death (fig S8).

**Fig 3:**
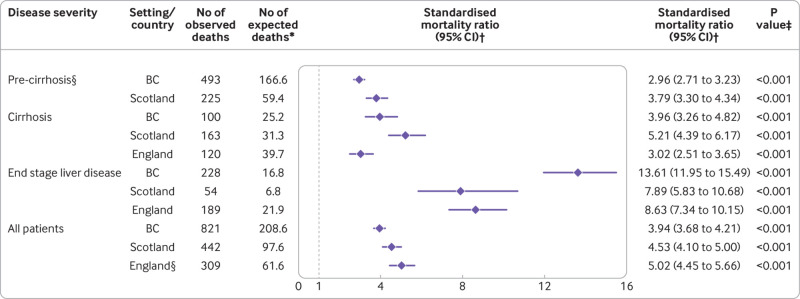
Standardised mortality ratio for all cause mortality by setting and liver disease severity. *Expected deaths indicates the number of deaths that would have occurred if people who had been successfully treated for hepatitis C had the same age-sex-year specific mortality rates as the corresponding general population. †Standardised mortality ratio is the ratio of observed to expected deaths; values >1 indicate excess mortality (number of observed deaths exceed number of expected deaths). ‡Null hypothesis is that standardised mortality ratio=1 (dashed line). §Mortality data for patients without cirrhosis (pre-cirrhosis) were not available for England cohort. 95% CI=95% confidence interval; BC=British Columbia

*Mortality relative to general population*—Mortality rates were considerably higher than those for the general population for all cohorts and disease stage groups (fig 3). In Scotland, the all cause mortality rate for all patients was 4.5 times greater than the general population, with 442 observed deaths versus 98 expected (standardised mortality ratio 4.53, 95% confidence interval 4.10 to 5.00; P<0.001). In British Columbia, mortality rates were 3.9 times greater, with 821 observed deaths versus 209 expected (3.94, 3.68 to 4.21; P<0.001). In England, the total number of observed deaths (309) was five times higher than the number expected (62; 5.02, 4.45 to 5.66; P<0.001); because this cohort did not include people without cirrhosis, this estimate cannot be directly compared with estimates for British Columbia and Scotland. Standardised mortality ratios increased appreciably with liver disease severity; for example, from 2.96 in people without cirrhosis from British Columbia (2.71 to 3.23; P<0.001) to 13.61 for patients with end stage liver disease in British Columbia (11.94 to 15.49; P<0.001; fig 3).

**Fig 4:**
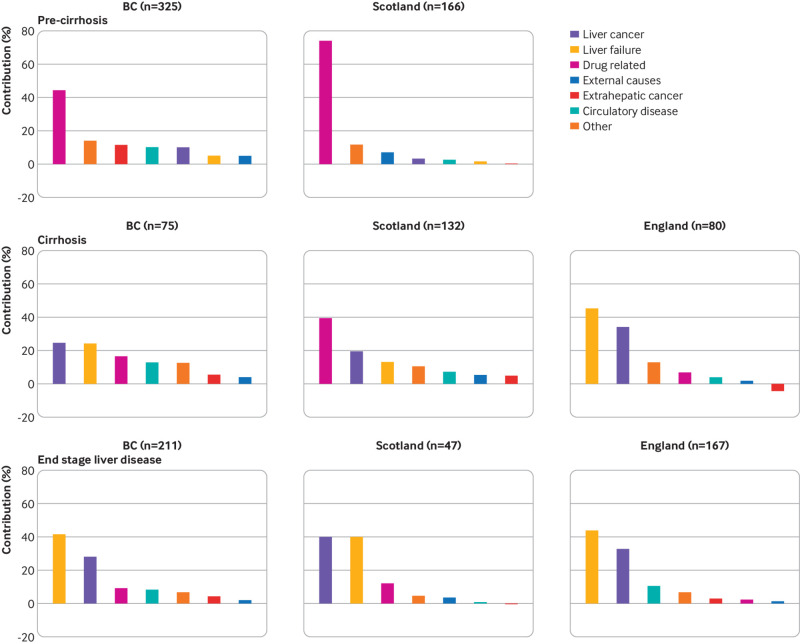
Cause specific contribution (%) to excess mortality by setting and liver disease severity. Excess mortality defined as number of observed deaths minus number of expected deaths (expected if the age-sex-year specific mortality rates in each cohort were identical to the corresponding general population). Total number of excess deaths for each setting and disease stage is given in brackets. Mortality data for patients without cirrhosis (pre-cirrhosis) were not available for England cohort. BC=British Columbia

Figures S12-S14 outline cause specific standardised mortality ratios. For patients without cirrhosis, the leading contributor to excess mortality was drug related death, accounting for 74% and 44% of all excess deaths in Scotland and British Columbia, respectively. Conversely, in patients with cirrhosis, the two leading drivers were liver cancer and liver failure; together, these causes accounted for up to 80% of excess deaths (fig 4). Standardised mortality ratios were attenuated when adjusting for area based deprivation in Scotland, but still indicated considerable excess mortality compared with the general population. For example, the standardised mortality ratio for all patients in Scotland was 3.35 (95% confidence interval 3.04 to 3.68; P<0.001) when adjusting for deprivation, age, sex, and year versus 4.53 (4.10 to 5.00; P<0.001) when adjusting for age, sex, and year alone (fig S15).

*Factors associated with mortality rate and standardised mortality ratio*—Across all disease stages and settings, older age was consistently associated with higher mortality rates in multivariable regression (table 2 and table S15). For example, in people without cirrhosis from Scotland, a 10-year increase in age was associated with a 30% increase in the mortality rate for all causes (mortality rate ratio 1.30, 95% confidence interval 1.14 to 1.49; P<0.001). Hospital admissions for alcohol and substance misuse before HCV cure were also associated with higher mortality. In general, the increase was greatest for patients with a recent admission versus a non-recent admission. For example, for people without cirrhosis from British Columbia, a recent substance misuse hospital admission was associated with almost a trebling in the mortality rate versus patients without a previous admission (mortality rate ratio 2.90, 95% confidence interval 2.17 to 3.88; P<0.001), whereas a non-recent admission was associated with a 2.2-fold increase (2.17, 1.67 to 2.83; P<0.001). Higher Charlson comorbidity index was also associated with greater mortality; these associations were nonlinear in some subgroups (fig S16).

**Table 2:** Factors associated with all cause mortality rate in multivariable analysis by setting and liver disease severity. Values are mortality rate ratios (95% confidence intervals); P values

**Characteristic**	British Columbia (n=11 942)				Scotland (n=7691)				England (n=2157)	
	Pre-cirrhosis	Cirrhosis	ESLD		Pre-cirrhosis	Cirrhosis	ESLD		Cirrhosis	ESLD
Year of SVR	1.05 (0.97 to 1.14); 0.19	0.85 (0.72 to 1.01); 0.06	0.78 (0.70 to 0.88); <0.001		0.99 (0.91 to 1.07); 0.75	0.97 (0.86 to 1.10); 0.67	1.00 (0.80 to 1.24); 0.97		1.02 (0.88 to 1.19); 0.79	0.93 (0.83 to 1.03); 0.15
Age per 10 year increase	1.38 (1.24 to 1.53); <0.001	1.46 (1.12 to 1.92); 0.006	1.47 (1.16 to 1.86); 0.002		1.30 (1.14 to 1.49); <0.001	1.25 (1.07 to 1.47); 0.006	1.31 (0.97 to 1.76); 0.08		1.43 (1.16 to 1.78); 0.001	1.30 (1.05 to 1.61); 0.02
Sex										
Male	Reference (1.00)	Reference (1.00)	Reference (1.00)		Reference (1.00)	Reference (1.00)	Reference (1.00)		Reference (1.00)	Reference (1.00)
Female	0.76 (0.62 to 0.93); 0.007	0.54 (0.33 to 0.87); 0.01	0.80 (0.59 to 1.08); 0.14		0.76 (0.55 to 1.05); 0.09	0.86 (0.59 to 1.25); 0.44	0.88 (0.48 to 1.61); 0.67		0.88 (0.56 to 1.39); 0.59	1.09 (0.78 to 1.53); 0.60
Hospital admission for alcohol misuse										
No	Reference (1.00)	Reference (1.00)	Reference (1.00)		Reference (1.00)	Reference (1.00)	Reference (1.00)		Reference (1.00)	Reference (1.00)
Yes (not recent)	1.04 (0.75 to 1.44); 0.83	1.49 (0.76 to 2.93); 0.25	1.28 (0.85 to 1.93); 0.24		1.16 (0.80 to 1.67); 0.44	1.19 (0.75 to 1.91); 0.46	0.72 (0.27 to 1.91); 0.51		1.28 (0.69 to 2.35); 0.43	0.72 (0.40 to 1.28); 0.26
Yes (recent)	1.95 (1.43 to 2.66); <0.001	2.32 (1.30 to 4.12); 0.004	1.45 (1.01 to 2.07); 0.04		1.73 (1.14 to 2.60); 0.009	1.51 (1.03 to 2.21); 0.04	1.49 (0.83 to 2.69); 0.18		1.61 (0.97 to 2.66); 0.07	1.63 (1.16 to 2.30); 0.005
Hospital admission for substance misuse										
No	Reference (1.00)	Reference (1.00)	Reference (1.00)		Reference (1.00)	Reference (1.00)	Reference (1.00)		Reference (1.00)	Reference (1.00)
Yes (not recent)	2.17 (1.67 to 2.83); <0.001	1.09 (0.57 to 2.08); 0.80	1.33 (0.84 to 2.07); 0.22		1.91 (1.36 to 2.68); <0.001	1.57 (1.05 to 2.35); 0.03	1.45 (0.66 to 3.19); 0.36		2.01 (1.12 to 3.59); 0.02	1.18 (0.64 to 2.18); 0.59
Yes (recent)	2.90 (2.17 to 3.88); <0.001	1.54 (0.70 to 3.38); 0.28	1.74 (1.11 to 2.72); 0.02		2.87 (2.00 to 4.12); <0.001	2.53 (1.69 to 3.78); <0.001	1.95 (1.00 to 3.80); 0.05		1.72 (1.08 to 2.74); 0.02	1.72 (1.22 to 2.43); 0.002
Charlson comorbidity index	0.99 (0.98 to 0.99)*; <0.001	1.22 (1.06 to 1.39); 0.005	1.22 (1.14 to 1.31); <0.001		1.24 (1.09 to 1.40); 0.001	1.35 (1.19 to 1.52); <0.001	1.13 (0.92 to 1.40); 0.25		1.18 (1.06 to 1.32); 0.004	1.02 (0.91 to 1.15); 0.69

ESLD=end stage liver disease; SVR=sustained viral response.

* First degree fractional polynomial: power=−2 (see figure S16).

We modelled the all cause standardised mortality ratio in addition to the all cause mortality rate (tables S16-S17). In general, each covariate’s association with the standardised mortality ratio mirrored its association with the mortality rate. However, a notable exception was older age, which was associated with a lower standardised mortality ratio, but a higher mortality rate. For example, for patients with compensated cirrhosis in Scotland, a 10 year increase in age was associated with a 43% reduction in standardised mortality ratio (0.57, 95% confidence interval 0.50 to 0.66; P<0.001), but also a 25% increase in mortality rate (rate ratio 1.25, 95% confidence interval 1.07 to 1.47; P=0.006).

## Discussion

### Principal findings

We used national data registries to present mortality rates in more than 20 000 people successfully treated for HCV with an interferon-free antiviral regimen. Our results indicate that people successfully treated for HCV show high rates of drug and liver related mortality, and that overall, mortality rates are considerably greater than the general population, even for patients without cirrhosis at the time of successful treatment. In Scotland, for example, we observed 442 deaths overall, whereas we would have expected only 98 deaths to have occurred had our cohort shown the same age-sex-year specific mortality rates as the general Scottish population. Standardised mortality ratios remained high even when adjusting for area based deprivation; therefore, the high mortality rates observed cannot be explained by generic health inequalities. Predictors of a higher mortality rate included recent hospital admission for alcohol and substance misuse and a greater comorbidity burden. Older age was associated with higher mortality, but standardised mortality ratios were greatest in younger patients.

### Policy implications

Our findings bring into focus the importance of establishing robust care and harm reduction pathways after successful HCV treatment. As we move towards HCV elimination, treatment programmes must strike the right balance between treating HCV and treating the patient. For example, patients with cirrhosis who have received successful HCV treatment benefit from liver cancer surveillance, yet these surveillance programmes are poorly implemented in the UK and other countries.^33^ Additionally, our data suggest patients need more support to reduce drug and alcohol misuse after HCV cure. Combining HCV treatment with wider intervention and wraparound services should be considered, especially because there is evidence that successful HCV treatment could be used as an opportunity to encourage changes in behaviour.^14^ ^34^ ^35^ Potential initiatives range from optimising delivery of established interventions (eg, referral pathways to addiction services, prescription of opioid agonist treatment,^36^ and drugs for alcohol dependence^37^) to more innovative approaches such as housing support interventions.^38^ Population level action—for example, prescribed safer supply of drugs and drug decriminalisation policies recently implemented in British Columbia—will also be crucial to improve mortality in people successfully treated for HCV.^39^ ^40^ Our results also have implications for public health surveillance of HCV. At present, the current emphasis (eg, in the UK 41) is on monitoring progress towards WHO mortality targets,^11^ which focus narrowly on deaths from viral hepatitis alone. In contrast, our study suggests a much wider lens is needed to understand the population impact of interferon-free treatments, and respond or adapt to the evolving landscape. New indicators should be introduced to convey the broader epidemiological context.

### Comparison with other studies

Our findings are consistent with previous data suggesting liver disease and excess mortality in the HCV population is a compound problem that can be mitigated, but not completely solved, with antiviral therapy.^42^ ^43^ ^44^ In a previous study from the United States, we estimated that half the excess mortality among people with chronic HCV can be attributed to health risk behaviours.^42^ The substantially increased risk of liver disease and mortality observed among people who are infected with HCV but clear the virus naturally in less than six months is also concordant with our findings.^43^ ^44^ Notably, our current analysis reports much higher levels of mortality than our previous population based study from Scotland, which was composed entirely of patients successfully treated for HCV using interferon based treatment regimens (2000-13).^17^ In this study, the rate of all cause mortality was only 7.1 deaths per 1000 person years, with a standardised mortality ratio of 1.86 (95% confidence interval 1.49 to 2.32). The main reason why the standardised mortality ratios in this study were at least two times higher is because the case mix of participants successfully treated for HCV has shifted considerably since interferon-free treatments became available. In particular, the present study included considerably more patients with cirrhosis or end stage liver disease, and with alcohol and substance misuse problems compared with our earlier Scottish study (table S18). Interferon-free treatment regimens have enabled more clinically challenging patients to be treated for HCV who are more representative of the general infected population than was possible with previous regimens. Therefore, while direct acting antivirals bring new opportunities, they also bring new challenges, for example, minimising competing risk events to maximise long term treatment benefit. Finally, in contrast to our findings, a recent study by D’Ambrosio and colleagues found no significant difference between the mortality of the general population and 480 patients with cirrhosis achieving SVR with an interferon-free treatment regimen, adjusting for age, sex, and year.^18^ Their results might have been subject to selection bias because patients were recruited from only a single liver centre. Additionally, some previous studies undertaken before interferon-free treatments were available, which were also based on patients attending select liver centres, have reported comparable mortality to the general population after adjusting for age, sex, and year.^15^ ^16^

### Limitations of this study

This study has several limitations which merit discussion. Our standardised mortality ratios were adjusted for high level variables only—age, sex, and calendar year. In our view, standardised mortality ratio adjustment for age, sex, and year is appropriate for describing the total burden of residual disease and ill health after cure. However, we were not able to adjust our standardised mortality ratios for more detailed clinical variables, such as alcohol and drug misuse, smoking, and so forth. While we did perform a sensitivity analysis incorporating additional adjustment for area based deprivation, it was only possible to perform this analysis for people in our Scottish cohort. Additionally, when interferon-free treatments first became available, the initial high cost, coupled with the large number of patients, led to health systems prioritising treatment in patients with advanced fibrosis.^45^ Therefore, in British Columbia, only patients with moderate to severe fibrosis (Metavir score F2-F4) were eligible for interferon-free treatments up until 2018.^46^ Similarly, in Scotland, patients with F4, and then F2-F4, were initially prioritised.^47^ In England, interferon-free treatments were first provided through the early access programme to patients with end stage liver disease before being expanded soon after to all patients with compensated cirrhosis.^48^ Although broadly similar, variability in prioritisation criteria between settings and how these were implemented might have led to artificial differences between our study settings. The eligibility restrictions described also imply that patients with minimal levels of fibrosis (Metavir F0-F2) will be under represented in cohorts of people without cirrhosis.

Another limitation is that alcohol and substance misuse before successful HCV treatment was inferred by hospital admissions; this approach does not capture milder levels of alcohol or substance misuse that do not lead to hospital admission but that might be relevant for prognosis. Also, the ICD codes used to infer ongoing alcohol and substance misuse (table S11) could have led to misclassification for some participants. In a similar vein, the ICD code recorded for underlying cause of death was used to infer the cause of death; however, it is possible that coding errors could have led to misclassification in some instances. We were unable to perform random effects meta-analysis due to the limited number of cohorts included in this study (n=3). Individual patient data meta-analysis was also not feasible because to conform with information governance requirements the three cohorts were accessed through separate trusted research environments. Therefore, it was not possible to analyse the data from one central place, which is a prerequisite for individual patient data meta-analysis.

Our study was composed of patients from high income countries where HCV transmission has been driven by injecting drug use; our results might not be generalisable to settings where the epidemiology differs. In the future, it would be useful to expand this study to include more diverse populations. The challenge, however, is that few populations have the necessary health registries and data linkage infrastructure in place to replicate our analysis. Additionally, we did not have recourse to data on liver blood tests, such as the fibrosis 4 index or the aspartate platelet ratio index, which are likely to be important prognostic factors for prognosis after HCV cure. Finally, our analysis did not directly account for HCV reinfection after a cure, which has increased considerably since interferon-free treatment regimens became available.^49^ Previous data suggest reinfection is less common in patients on opioid agonist treatment and among those who engage in mental health counselling.^50^ These findings further support the rationale for a holistic approach—that is, antiviral treatment alongside broader health interventions.

### Conclusions

In summary, we have performed a large study of mortality rates in more than 20 000 people who have been successfully treated for HCV. Our results show that these people continue to face substantial mortality rates, driven by liver and drug related causes. These findings highlight the importance of establishing robust follow-up pathways after successful HCV treatment as we move towards HCV elimination.

**What is already known on this topic**

Interferon-free antivirals have transformed the treatment of chronic hepatitis C infection; treatment is successful in more than 95% of patients
Patients who are successfully treated show better health outcomes than untreated patients (eg, liver disease progression, diseases outside the liver, and all cause mortality)
Countries are moving towards hepatitis C elimination, but the prognosis after successful treatment remains questionable

**What this study adds**

People who have received successful hepatitis C treatment show high mortality rates that are considerably greater than the general population (between 3 and 14 times higher depending on liver disease stage)
Excess mortality is largely driven by drug related causes, liver failure, and liver cancer; recent hospital admissions for alcohol and substance misuse were predictors of higher mortality rates and standardised mortality ratios
With substantial drug and liver related mortality after successful hepatitis C treatment, services and interventions to prevent drug and alcohol related harms are needed

## Data Availability

The Scottish data used in this study are not publicly available, but can be acquired through successful application to the Public Benefit and Privacy Panel for Health and Social Care (https://www.informationgovernance.scot.nhs.uk/pbpphsc/home/for-applicants/ ). HCVRUK participant data are accessible through application to the HCVRUK Tissue Data Access Committee. Contact Professor William Irving (will.irving@nottingham.ac.uk) for more information. Linked registry data cannot be shared with other research groups. However, linked data can be accessed independently following successful application to NHS digital Data Access Request Service (DARS; for more information, see https://digital.nhs.uk/services/data-access-request-service-dars). Data from the British Columbia hepatitis testers cohort are not publicly available. Access to the data might be provided through the British Columbia Centre for Disease Control Institutional Data Access process to researchers who meet the criteria for accessing confidential data. Information on data access is available at BC Centre for Disease Control at the following link: http://www.bccdc.ca/about/accountability/data-access-requests

## Web extra

Extra material supplied by authors

Web appendix: Supplementary tables, figures, and appendices

## Notes

Contributors: VH, SW, SH, EB, ING, WLI, NZJ, and HI contributed to study conception (lead contributor was HI). VH, SW, JB, NZJ, and HI contributed equally to data curation, including activities to clean and maintain research data. Formal analysis was performed by VH (Scotland data), SW (British Columbia data), and HI (Scotland and England data). SH, EB, ING, WLI, NZJ, and HI contributed to funding acquisition (lead contributor was HI). All authors contributed to the research and investigation process, including the collection of data. VH, SW, NZJ, and HI contributed equally to the methods and study design. VH, SW, JB, NZJ, and HI contributed equally to project administration (lead contributor was VH). SH, EB, ING, WLI, NZJ, and HI contributed to supervision, including leadership responsibility (lead contributor was HI). VH, SW, NZJ, and HI wrote the initial manuscript (lead contributors were VH and HI). All authors contributed to subsequent editing and revising of the study manuscript. VH and SW have contributed equally and are joint first authors. HI is the study guarantor and attests that all listed authors meet authorship criteria and that no others meeting the criteria have been omitted.

Patient consent: Informed consent was not obtained for people in the British Columbia hepatitis testers cohort or the Scottish viral hepatitis national databases, as these are deidentified secondary use data, collected for public health surveillance and routine healthcare. Written informed consent was obtained for all people in the HCVRUK England cohort as HCVRUK constitutes a research study rather than a public health surveillance system.

The senior author (the manuscript’s guarantor) affirms that the manuscript is an honest, accurate, and transparent account of the study being reported; that no important aspects of the study have been omitted; and that any discrepancies from the study as planned (and, if relevant, registered) have been explained.

Dissemination to participants and related patient and public communities: We will disseminate our findings to the hepatitis C patient community and the broader public via press releases, social media posts on Twitter and via prominent third sector organisations in the UK and British Columbia. The results will also be shared with key policy and public health groups across the UK, such as the National Strategic Group for Viral hepatitis (NSGVH), in addition to contacts at the World Health Organization.

Provenance and peer review: Not commissioned; externally peer reviewed.

## References

[1] Pawlotsky JM Feld JJ Zeuzem S Hoofnagle JH . From non-A, non-B hepatitis to hepatitis C virus cure. J Hepatol 2015;62(Suppl):S87-99. 10.1016/j.jhep.2015.02.006 25920094

[2] Fried MW . Side effects of therapy of hepatitis C and their management. Hepatology 2002; 36(S1):S237-44. 12407599 10.1053/jhep.2002.36810

[3] Hopwood M Treloar C Resull L . Experiences of hepatitis C treatment and its management: what some patients and health professionals say (Monograph 4/2006). National Centre in HIV Social Research, The University of New South Wales, 2006.

[4] Thomson BJ Kwong G Ratib S Trent HCV Study Group . Response rates to combination therapy for chronic HCV infection in a clinical setting and derivation of probability tables for individual patient management. J Viral Hepat 2008;15:271-8. 10.1111/j.1365-2893.2007.00941.x 18086181

[5] Innes HA Hutchinson SJ Allen S Hepatitis C Clinical Database Monitoring Committee . Ranking predictors of a sustained viral response for patients with chronic hepatitis C treated with pegylated interferon and ribavirin in Scotland. Eur J Gastroenterol Hepatol 2012;24:646-55. 10.1097/MEG.0b013e32835201a4 22433796

[6] Drysdale K Ntuli Y Bestwick J . English hepatitis C registry data show high response rates to directly acting anti-virals, even if treatment is not completed. Aliment Pharmacol Ther 2020;52:168-81. 10.1111/apt.15780 32441382

[7] Innes H McDonald SA Hamill V . Declining incidence of hepatitis C related hepatocellular carcinoma in the era of interferon-free therapies: A population-based cohort study. Liver Int 2022;42:561-74. 10.1111/liv.15143 34951109

[8] European Association for the Study of the Liver . EASL recommendations on treatment of hepatitis C 2014. J Hepatol 2014;61:373-95. 10.1016/j.jhep.2014.05.001 24818984

[9] Saab S Le L Saggi S Sundaram V Tong MJ . Toward the elimination of hepatitis C in the United States. Hepatology 2018;67:2449-59. 10.1002/hep.29685 29181853

[10] Gamkrelidze I Pawlotsky JM Lazarus JV . Progress towards hepatitis C virus elimination in high-income countries: An updated analysis. Liver Int 2021;41:456-63. 10.1111/liv.14779 33389788

[11] World Health Organization. Global health sector strategy on viral hepatitis 2016-2021. Towards ending viral hepatitis. Switzerland: World Health Organization; 2016. Available from: https://apps.who.int/iris/bitstream/handle/10665/246177/WHO-HIV-2016.06-eng.pdf

[12] Janjua NZ Wong S Abdia Y . Impact of direct-acting antivirals for HCV on mortality in a large population-based cohort study. J Hepatol 2021;75:1049-57. 10.1016/j.jhep.2021.05.028 34097994

[13] Carrat F Fontaine H Dorival C French ANRS CO22 Hepather cohort . Clinical outcomes in patients with chronic hepatitis C after direct-acting antiviral treatment: a prospective cohort study. Lancet 2019;393:1453-64. 10.1016/S0140-6736(18)32111-1 30765123

[14] Innes HA McDonald SA Dillon JF . Toward a more complete understanding of the association between a hepatitis C sustained viral response and cause-specific outcomes. Hepatology 2015;62:355-64. 10.1002/hep.27766 25716707

[15] van der Meer AJ Wedemeyer H Feld JJ . Life expectancy in patients with chronic HCV infection and cirrhosis compared with a general population. JAMA 2014;312:1927-8. 10.1001/jama.2014.12627 25387192

[16] Bruno S Di Marco V Iavarone M . Survival of patients with HCV cirrhosis and sustained virologic response is similar to the general population. J Hepatol 2016;64:1217-23. 10.1016/j.jhep.2016.01.034 27059129

[17] Innes H McDonald S Hayes P . Mortality in hepatitis C patients who achieve a sustained viral response compared to the general population. J Hepatol 2017;66:19-27. 10.1016/j.jhep.2016.08.004 27545496

[18] D’Ambrosio R Degasperi E Anolli MP . Incidence of liver- and non-liver-related outcomes in patients with HCV-cirrhosis after SVR. J Hepatol 2022;76:302-10. 10.1016/j.jhep.2021.09.013 34592366

[19] Janjua NZ Yu A Kuo M . Twin epidemics of new and prevalent hepatitis C infections in Canada: BC Hepatitis Testers Cohort. BMC Infect Dis 2016;16:334. 10.1186/s12879-016-1683-z 27436414 PMC4952323

[20] Janjua NZ Kuo M Chong M . Assessing hepatitis C burden and treatment effectiveness through the British Columbia Hepatitis Testers Cohort (BC-HTC): design and characteristics of linked and unlinked participants. PLoS One 2016;11:e0150176. 10.1371/journal.pone.0150176 26954020 PMC4783072

[21] McDonald SA Hutchinson SJ Innes HA . Attendance at specialist hepatitis clinics and initiation of antiviral treatment among persons chronically infected with hepatitis C: examining the early impact of Scotland’s Hepatitis C Action Plan. J Viral Hepat 2014;21:366-76. 10.1111/jvh.12153 24716639

[22] McLeod A Glancy M Went A . Surveillance of hepatitis C testing, diagnosis and treatment in Scotland, 2019 update. Scotland. NHS, 2019.

[23] McLauchlan J Innes H Dillon JF HCV Research UK Steering Committee . Cohort profile: the hepatitis C virus (HCV) research UK clinical database and biobank. Int J Epidemiol 2017;46:1391-1391h. 10.1093/ije/dyw362 28338838 PMC5837619

[24] Herbert A Wijlaars L Zylbersztejn A Cromwell D Hardelid P . Data resource profile: hospital episode statistics admitted patient care (HES APC). Int J Epidemiol 2017;46:1093-1093i. 10.1093/ije/dyx015 28338941 PMC5837677

[25] Diagnostic Imaging Dataset NHS . 2012. Available from: https://www.england.nhs.uk/statistics/statistical-work-areas/diagnostic-imaging-dataset/

[26] Henson KE Elliss-Brookes L Coupland VH . Data resource profile: national cancer registration dataset in England. Int J Epidemiol 2020;49:16-16h. 10.1093/ije/dyz076 31120104 PMC7124503

[27] Yoshida EM Sulkowski MS Gane EJ . Concordance of sustained virological response 4, 12, and 24 weeks post-treatment with sofosbuvir-containing regimens for hepatitis C virus. Hepatology 2015;61:41-5. 10.1002/hep.27366 25314116

[28] Pearlman BL Traub N . Sustained virologic response to antiviral therapy for chronic hepatitis C virus infection: a cure and so much more. Clin Infect Dis 2011;52:889-900. 10.1093/cid/cir076 21427396

[29] Charlson ME Pompei P Ales KL MacKenzie CR . A new method of classifying prognostic comorbidity in longitudinal studies: development and validation. J Chronic Dis 1987;40:373-83. 10.1016/0021-9681(87)90171-8 3558716

[30] Quan H Sundararajan V Halfon P . Coding algorithms for defining comorbidities in ICD-9-CM and ICD-10 administrative data. Med Care 2005;43:1130-9. 10.1097/01.mlr.0000182534.19832.83 16224307

[31] Quan H Li B Couris CM . Updating and validating the Charlson comorbidity index and score for risk adjustment in hospital discharge abstracts using data from 6 countries. Am J Epidemiol 2011;173:676-82. 10.1093/aje/kwq433 21330339

[32] Royston P Ambler G Sauerbrei W . The use of fractional polynomials to model continuous risk variables in epidemiology. Int J Epidemiol 1999;28:964-74. 10.1093/ije/28.5.964 10597998

[33] Hamill V Gelson W MacDonald D . Delivery of biannual ultrasound surveillance for individuals with cirrhosis and cured hepatitis C in the UK. Liver Int 2023;43:917-27. 10.1111/liv.15528 36708150 PMC10946603

[34] Martin M Roth PJ Niu J . Changes in alcohol use during hepatitis C treatment in persons who inject drugs. J Viral Hepat 2022;29:1004-14. 10.1111/jvh.13737 35997620 PMC9826277

[35] Donaldson SR Radley A Dillon JF . Transformation of identity in substance use as a pathway to recovery and the potential of treatment for hepatitis C: a systematic review. BMJ Open 2022;12:e049713. 10.1136/bmjopen-2021-049713 35131816 PMC8823084

[36] Sordo L Barrio G Bravo MJ . Mortality risk during and after opioid substitution treatment: systematic review and meta-analysis of cohort studies. BMJ 2017;357:j1550. 10.1136/bmj.j1550 28446428 PMC5421454

[37] Goh ET Morgan MY . Review article: pharmacotherapy for alcohol dependence—the why, the what and the wherefore. Aliment Pharmacol Ther 2017;45:865-82. 10.1111/apt.13965 28220511

[38] Miller-Archie SA Walters SC Bocour A . The impact of supportive housing on liver-related outcomes among persons with hepatitis C virus infection. J Infect Dis 2022;226(Suppl 3):S363-71. 10.1093/infdis/jiac292 36208165 PMC9547527

[39] BC Gov News. BC introduces new prescribed safer supply policy, a Canadian first. 2021. Available from: https://news.gov.bc.ca/releases/2021MMHA0035-001375

[40] BC Gov News. BC receives exemption to decriminalize possession of some illegal drugs for personal use. 2022. Available from: https://news.gov.bc.ca/releases/2022MMHA0029-000850

[41] UK Health Security Agency. Hepatitis C in England 2022. Working to eliminate hepatitis C as a public health problem. Full report. England (UK): UK Health Security Agency; 2022. Available from: https://assets.publishing.service.gov.uk/government/uploads/system/uploads/attachment_data/file/1057271/HCV-in-England-2022-full-report.pdf

[42] Innes H McAuley A Alavi M Valerio H Goldberg D Hutchinson SJ . The contribution of health risk behaviors to excess mortality in American adults with chronic hepatitis C: A population cohort-study. Hepatology 2018;67:97-107. 10.1002/hep.29419 28777874

[43] Innes H Hutchinson SJ Obel N . Liver mortality attributable to chronic hepatitis C virus infection in Denmark and Scotland--using spontaneous resolvers as the benchmark comparator. Hepatology 2016;63:1506-16. 10.1002/hep.28458 26773546

[44] Omland LH Osler M Jepsen P . Socioeconomic status in HCV infected patients—risk and prognosis. Clin Epidemiol 2013;5:163-72. 10.2147/CLEP.S43926 23766659 PMC3678712

[45] Marshall AD Pawlotsky JM Lazarus JV Aghemo A Dore GJ Grebely J . The removal of DAA restrictions in Europe—one step closer to eliminating HCV as a major public health threat. J Hepatol 2018;69:1188-96. 10.1016/j.jhep.2018.06.016 29959953

[46] Bartlett SR Yu A Chapinal N . The population level care cascade for hepatitis C in British Columbia, Canada as of 2018: impact of direct acting antivirals. Liver Int 2019;39:2261-72. 10.1111/liv.14227 31444846

[47] Health Protection Scotland and the Scottish Government. The Scottish Government hepatitis C treatment and therapies group report. Scotland (UK): The Scottish Government; 2017. Available from: https://www.gcu.ac.uk/__data/assets/pdf_file/0020/28901/hepc_treatment_report_feb2017.pdf

[48] NHS England Clinical Reference Group for Infectious Diseases. Clinical commissioning policy statement: treatment of chronci hepatitis C in patients with cirrhosis. England (UK): NHS England; 2015. Available from: https://www.england.nhs.uk/commissioning/wp-content/uploads/sites/12/2015/06/hep-c-cirrhosis-polcy-statmnt-0615.pdf

[49] Yeung A Palmateer NE Dillon JF . Population-level estimates of hepatitis C reinfection post scale-up of direct-acting antivirals among people who inject drugs. J Hepatol 2022;76:549-57. 10.1016/j.jhep.2021.09.038 34634387 PMC8852744

[50] Islam N Krajden M Shoveller J British Columbia Hepatitis Testers Cohort (BC-HTC) team . Incidence, risk factors, and prevention of hepatitis C reinfection: a population-based cohort study. Lancet Gastroenterol Hepatol 2017;2:200-10. 10.1016/S2468-1253(16)30182-0 28404135

