# Supplementary material for: Mortality rates among patients successfully treated for hepatitis C in the era of interferon-free antivirals: population based cohort study
Source: BMJ. 2023 Aug 2;382:e074001. doi: 10.1136/bmj-2022-074001 (PMC10394680; doi:10.1136/bmj-2022-074001)
Supplement: Supplementary file 1 — Web appendix: Supplementary tables, figures, and appendices [file hamv074001.ww.pdf]

Mortality rates among patients successfully treated for hepatitis C in the era of interferon-free antivirals: population based cohort study

AUTHORS: Hamill V, Wong S, Benselin J, Krajden M, Hayes PC, Mutimer D, Yu A, Dillon JF, Gelson W, Velásquez García HA, Yeung A, Johnson P, Barclay ST, Alvarez M, Toyoda H, Agarwal K, Fraser A, Bartlett S, Aldersley M, Bathgate A, Binka M, Richardson P, Morling JR, Ryder SD, MacDonald D, Hutchinson S, Barnes E, Guha IN, Irving W, Janjua NZ, Innes H.

Table of contents

Supplementary Figure 1……………………………………………………………..3

Supplementary Figure 2……………………………………………………………..4

Supplementary Figure 3……………………………………………………………..5

Supplementary Figure 4……………………………………………………………..6

Supplementary Figure 5……………………………………………………………..7

Supplementary Figure 6……………………………………………………………..8

Supplementary Figure 7……………………………………………………………..9

Supplementary Figure 8……………………………………………………………10

Supplementary Figure 9……………………………………………………………11

Supplementary Figure 10...…………………………………………………………12

Supplementary Figure 11...…………………………………………………………13

Supplementary Figure 12...…………………………………………………………14

Supplementary Figure 13...…………………………………………………………15

Supplementary Figure 14...…………………………………………………………16

Supplementary Figure 15...…………………………………………………………17

Supplementary Figure 16...…………………………………………………………18

Supplementary Table 1……………………………………………………………19

Supplementary Table 2……………………………………………………………20

Supplementary Table 3……………………………………………………………21

Supplementary Table 4……………………………………………………………22

Supplementary Table 5……………………………………………………………23

Supplementary Table 6……………………………………………………………24

Supplementary Table 7……………………………………………………………25

Supplementary Table 8……………………………………………………………26

Supplementary Table 9……………………………………………………………27

Supplementary Table 10…………………………………………………………28

Supplementary Table 11…………………………………………………………29

Supplementary Table 12…………………………………………………………30

Supplementary Table 13…………………………………………………………31

Supplementary Table 14…………………………………………………………32

Supplementary Table 15…………………………………………………………33

Supplementary Table 16…………………………………………………………33

Supplementary Table 17…………………………………………………………34

Supplementary Table 18…………………………………………………………35

Appendix A…………………………………………………………………...36-37

Appendix B…………………………………………………………………...37-38

Figure S1. Age at hepatitis C cure: distribution according to setting and liver disease severity

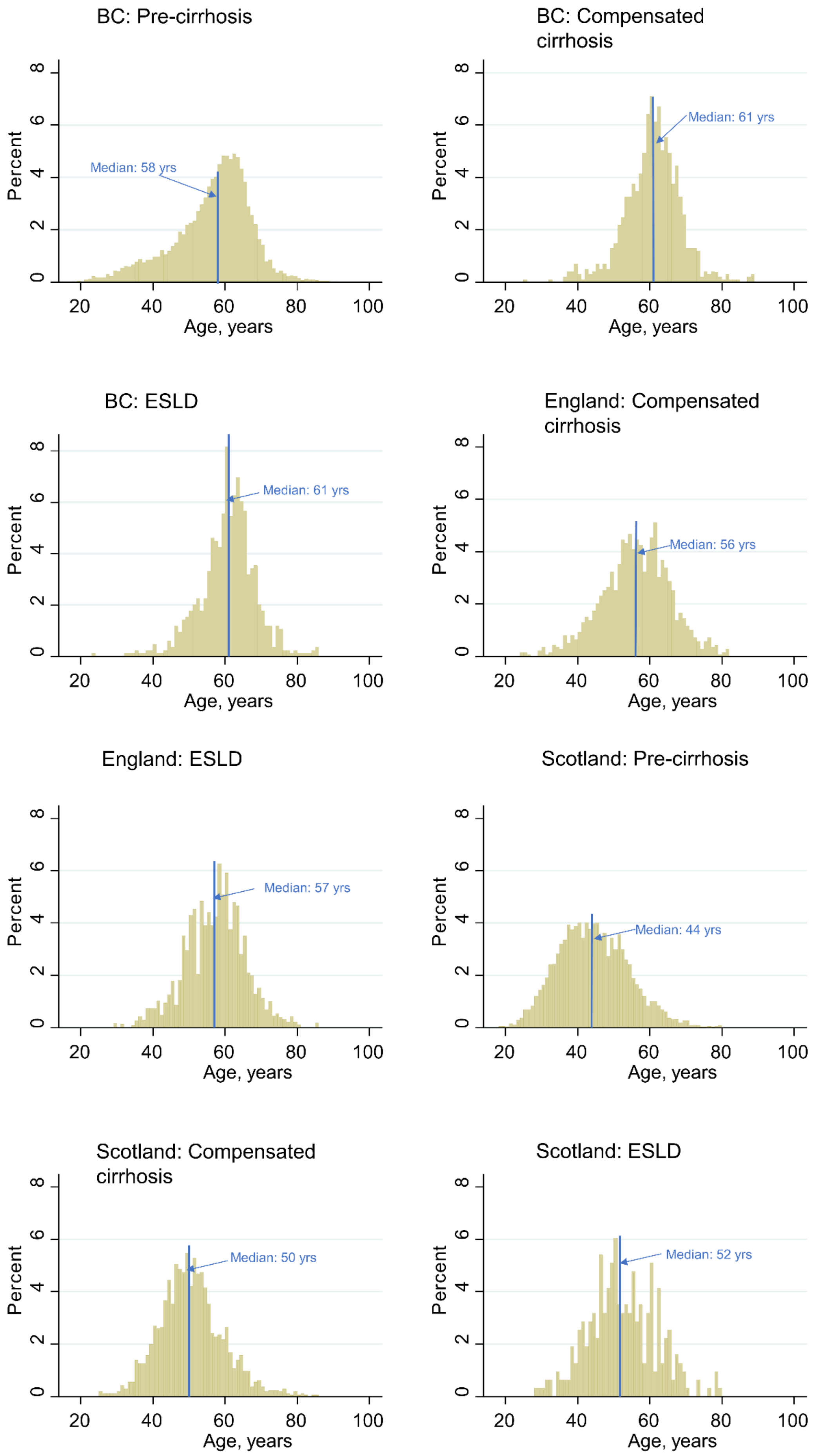

Figure S2. Date of hepatitis C cure: distribution according to setting and liver disease severity

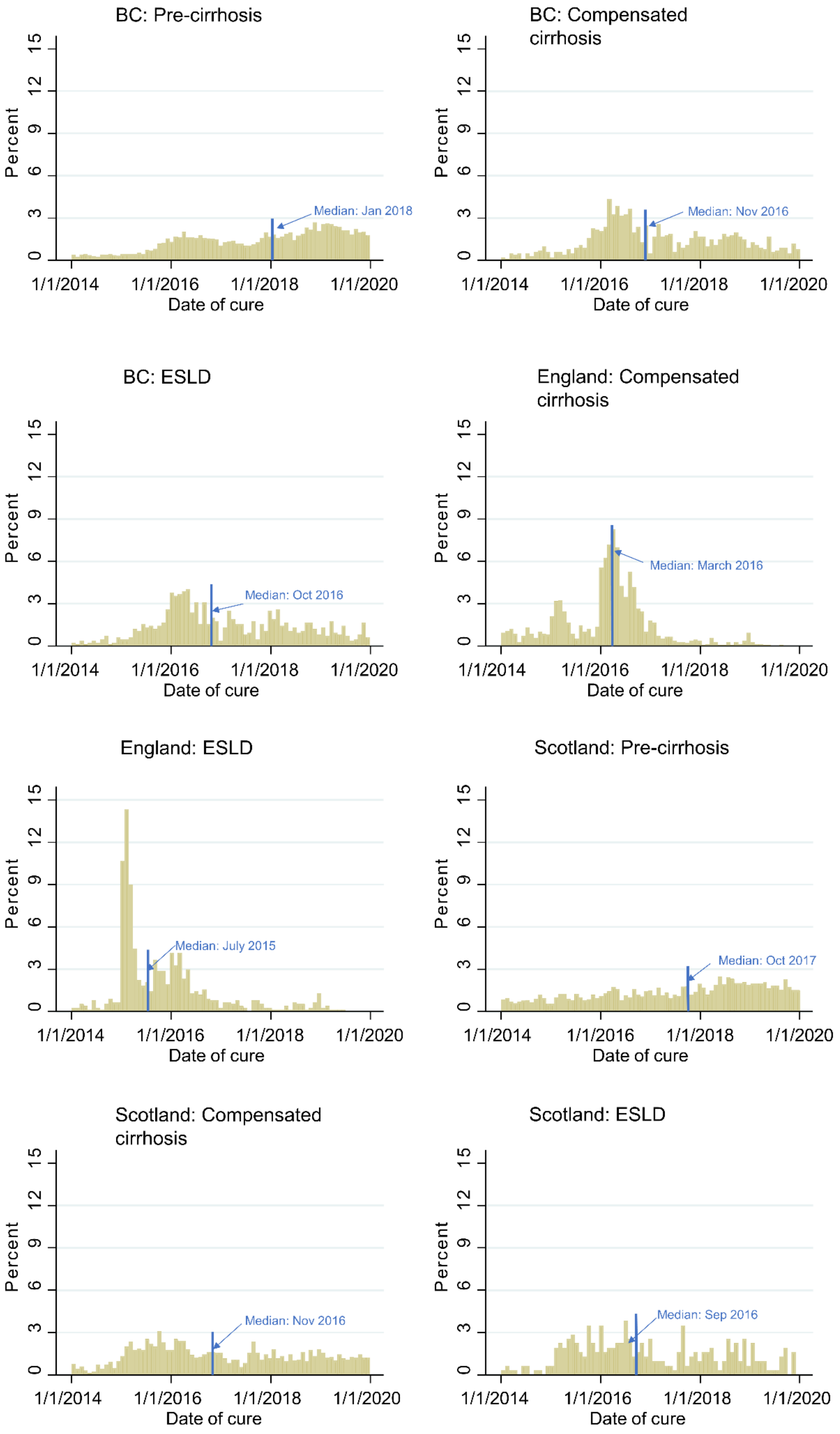

Figure S3. Proportion of individuals with a pre-cure alcohol and substance use hospital admission, by setting and liver disease severity

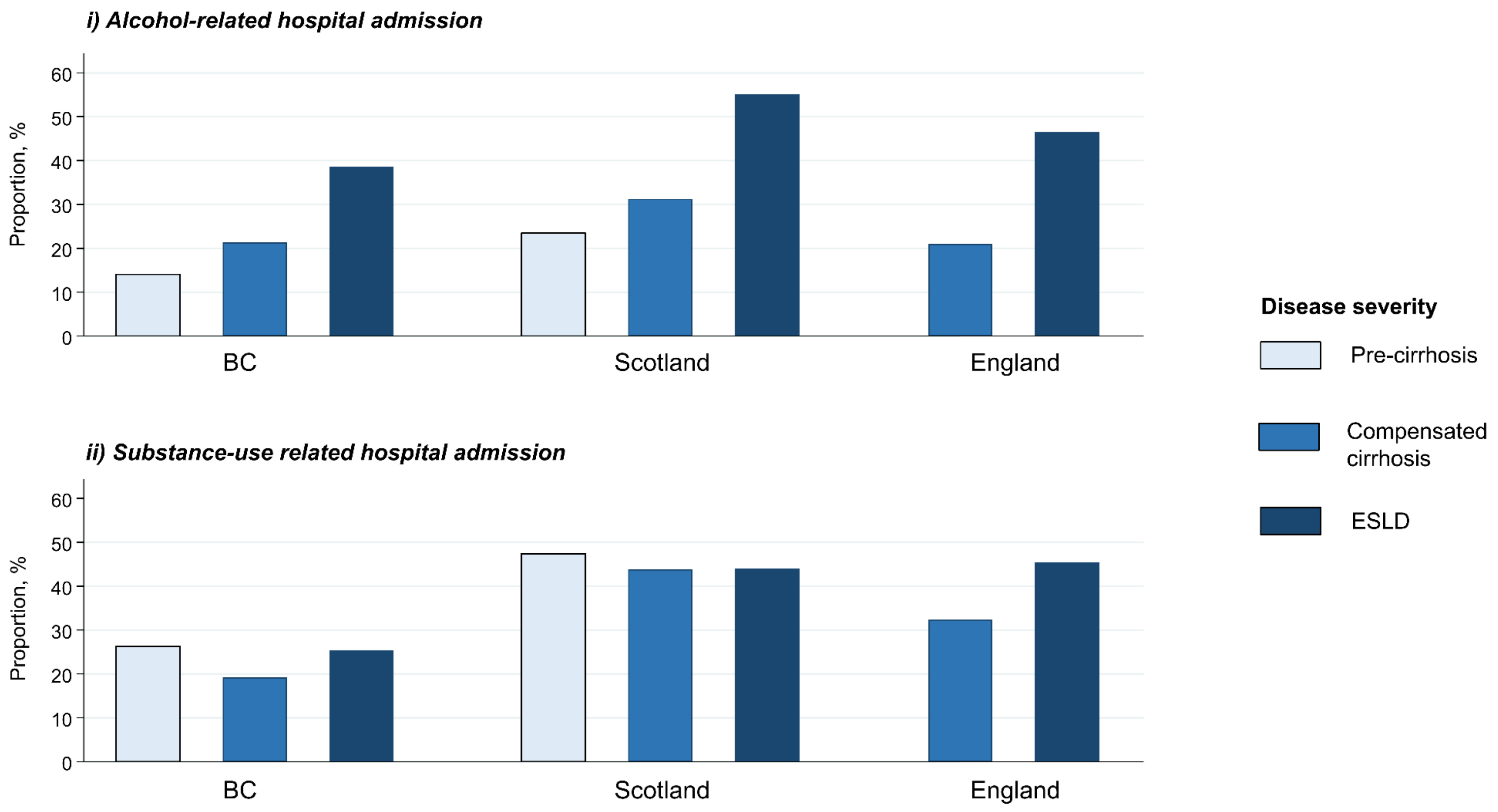

N.B. All proportions combine recent and historical admissions. The ICD codes used to define these events are outlined in Table S11.

Figure S4. Crude mortality rates for individuals with ESLD (excluding HCC), by cause of death and setting

Cause of death	Setting/ Country	No. Individuals	**Mortality rate:** No. deaths per 1000 PYs (95% CI)
All causes	BC	667	108.26 (92.46 to 125.98)
	Scotland	228	63.33 (45.89 to 87.41)
	England	509	72.33 (61.14 to 85.56)
Liver cancer	BC	667	19.45 (13.12 to 27.76)
	Scotland	228	10.27 (4.61 to 22.86)
	England	509	13.30 (8.98 to 19.68)
Liver failure	BC	667	46.03 (35.95 to 58.05)
	Scotland	228	29.10 (18.09 to 46.81)
	England	509	34.04 (26.64 to 43.49)
Drug-related	BC	667	12.32 (7.42 to 19.23)
	Scotland	228	8.56 (3.56 to 20.56)
	England	509	2.13 (0.80 to 5.67)
External causes	BC	667	2.59 (0.71 to 6.64)
	Scotland	228	3.42 (0.86 to 13.69)
	England	509	1.06 (0.27 to 4.25)
Extrahepatic cancer	BC	667	8.43 (4.49 to 14.41)
	Scotland	228	3.42 (0.86 to 13.69)
	England	509	3.19 (1.43 to 7.10)
Circulatory disease	BC	667	11.02 (6.42 to 17.64)
	Scotland	228	3.42 (0.86 to 13.69)
	England	509	10.64 (6.86 to 16.49)
Other	BC	667	8.43 (4.49 to 14.41)
	Scotland	228	5.13 (1.66 to 15.92)
	England	509	7.98 (4.81 to 13.23)

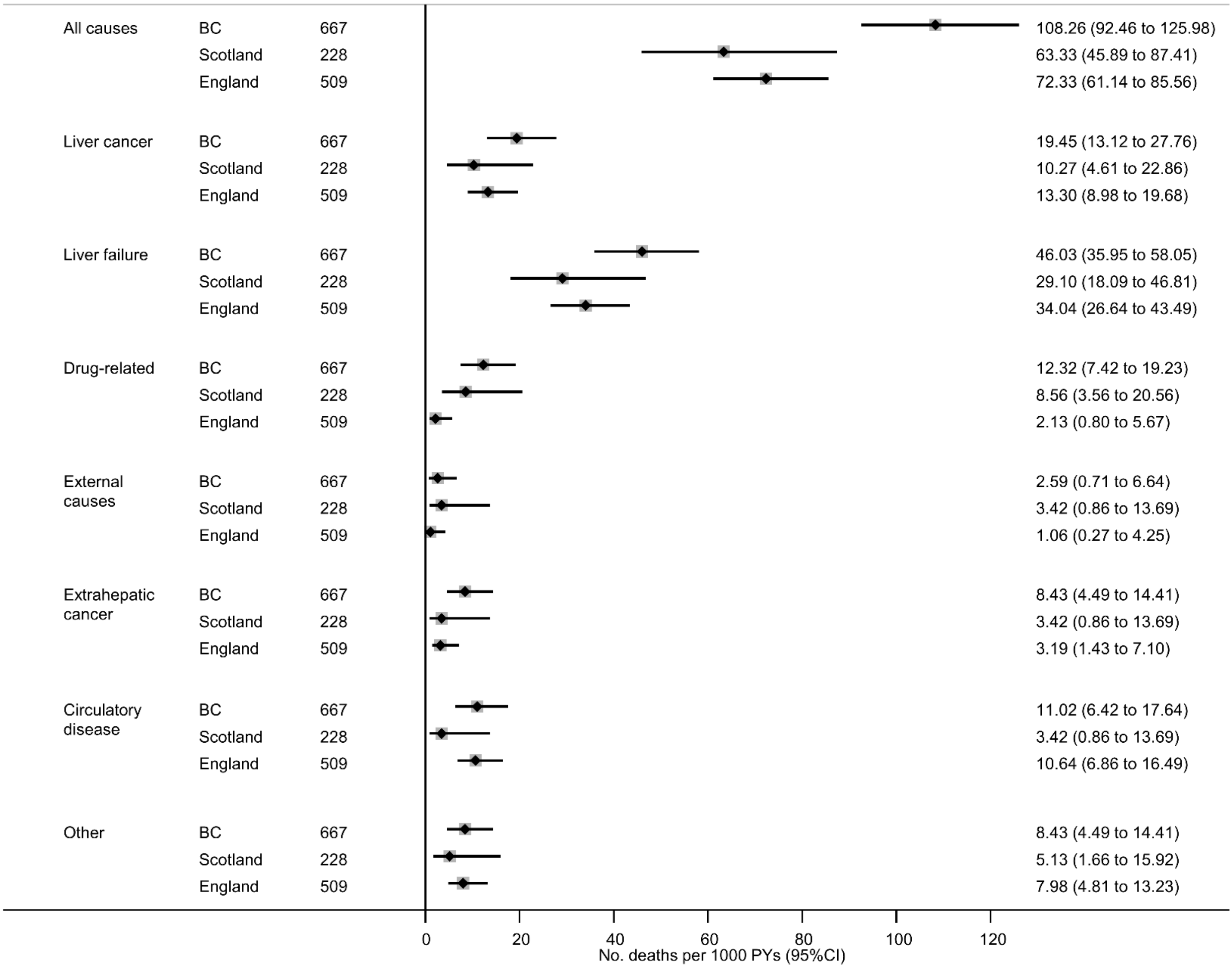

ESLD refers to End stage liver disease; HCC = hepatocellular carcinoma; PYs= person years

Figure S5. Standardised mortality rates for individuals with ESLD (excluding HCC), by cause of death and setting

Cause of death	Setting/ Country	No. Individuals	**Mortality rate:** No. deaths per 1000 PYs (95% CI)
All causes	BC	667	108.26 (92.46 to 125.98)
	Scotland	228	50.02 (27.66 to 72.38)
	England	509	74.22 (60.60 to 87.85)
Liver cancer	BC	667	19.45 (13.12 to 27.76)
	Scotland	228	13.97 (0.09 to 27.85)
	England	509	12.85 (7.24 to 18.46)
Liver failure	BC	667	46.03 (35.95 to 58.05)
	Scotland	228	15.56 (5.23 to 25.89)
	England	509	33.64 (24.46 to 42.81)
Drug-related	BC	667	12.32 (7.42 to 19.23)
	Scotland	228	4.69 (0.00 to 10.50)
	England	509	1.19 (0.00 to 2.64)
External causes	BC	667	2.59 (0.71 to 6.64)
	Scotland	228	3.40 (0.00 to 9.02)
	England	509	0.70 (0.00 to 1.71)
Extrahepatic cancer	BC	667	8.43 (4.49 to 14.41)
	Scotland	228	7.15 (0.00 to 18.65)
	England	509	3.98 (0.53 to 7.43)
Circulatory disease	BC	667	11.02 (6.42 to 17.64)
	Scotland	228	3.96 (0.00 to 9.71)
	England	509	14.04 (7.43 to 20.66)
Other	BC	667	8.43 (4.49 to 14.41)
	Scotland	228	1.30 (0.00 to 2.78)
	England	509	7.84 (3.22 to 12.46)

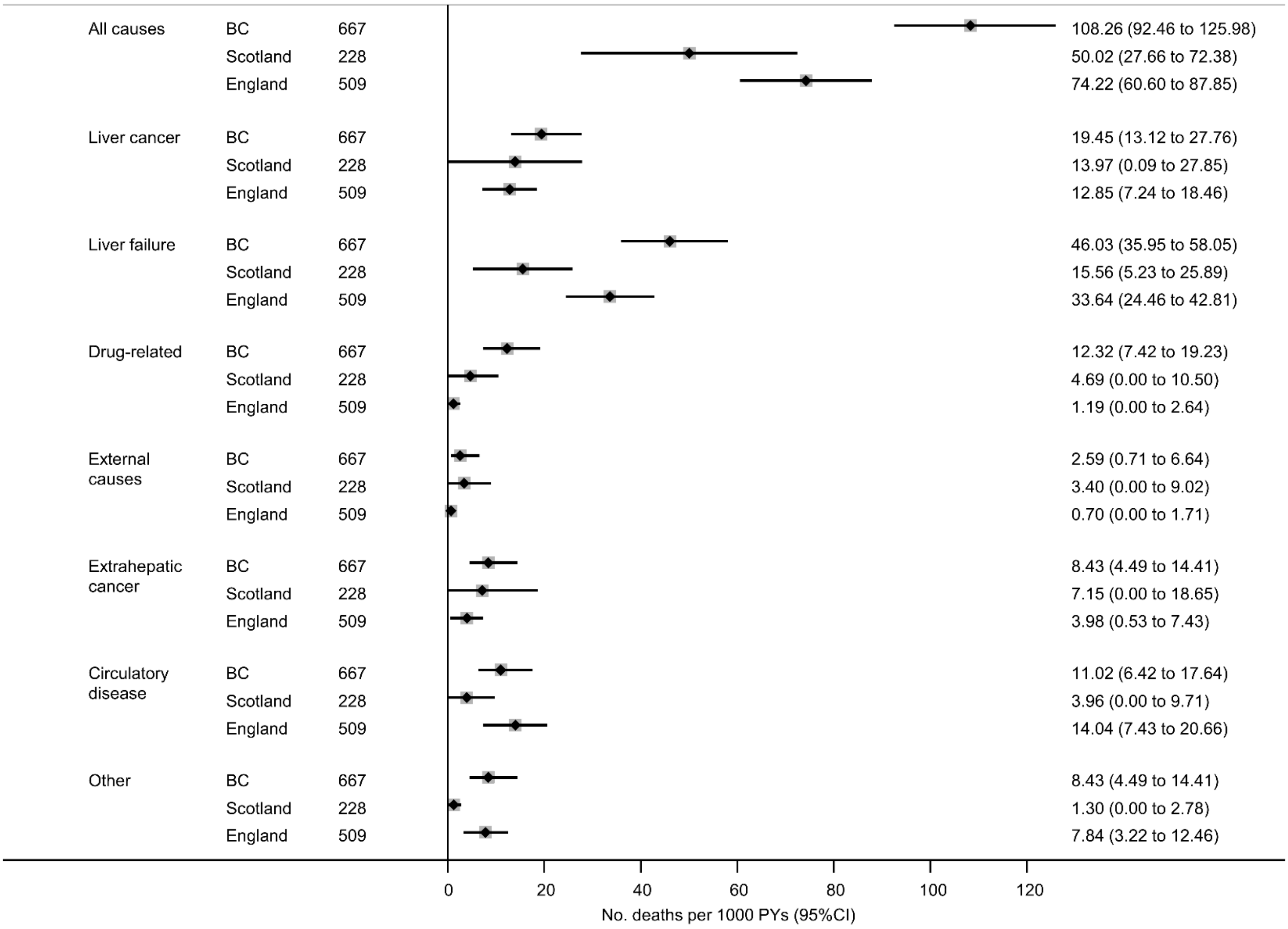

Mortality rates are standardized for age and sex, using ESLD patients from BC as the standard population. See Table S12 for further details.
ESLD refers to End stage liver disease. HCC refers to hepatocellular carcinoma; PYs refers to person years

Figure S6. Crude mortality rates for pre-cirrhosis individuals, by cause of death and setting

Cause of death	Setting/ Country	No. deaths	**Mortality rate:** No. deaths per 1000 PYs (95% CI)
All causes	BC	493	22.89 (20.91 to 25.00)
	Scotland	225	16.10 (14.13 to 18.35)
Liver cancer	BC	37	1.72 (1.21 to 2.37)
	Scotland	6	0.43 (0.19 to 0.96)
Liver failure	BC	23	1.07 (0.68 to 1.60)
	Scotland	6	0.43 (0.19 to 0.96)
Drug-related	BC	150	6.96 (5.89 to 8.17)
	Scotland	130	9.30 (7.84 to 11.05)
External causes	BC	24	1.11 (0.71 to 1.66)
	Scotland	17	1.22 (0.76 to 1.96)
Extrahepatic cancer	BC	95	4.41 (3.57 to 5.39)
	Scotland	16	1.15 (0.70 to 1.87)
Circulatory disease	BC	68	3.16 (2.45 to 4.00)
	Scotland	17	1.22 (0.76 to 1.96)
Other	BC	96	4.46 (3.61 to 5.44)
	Scotland	33	2.36 (1.68 to 3.32)

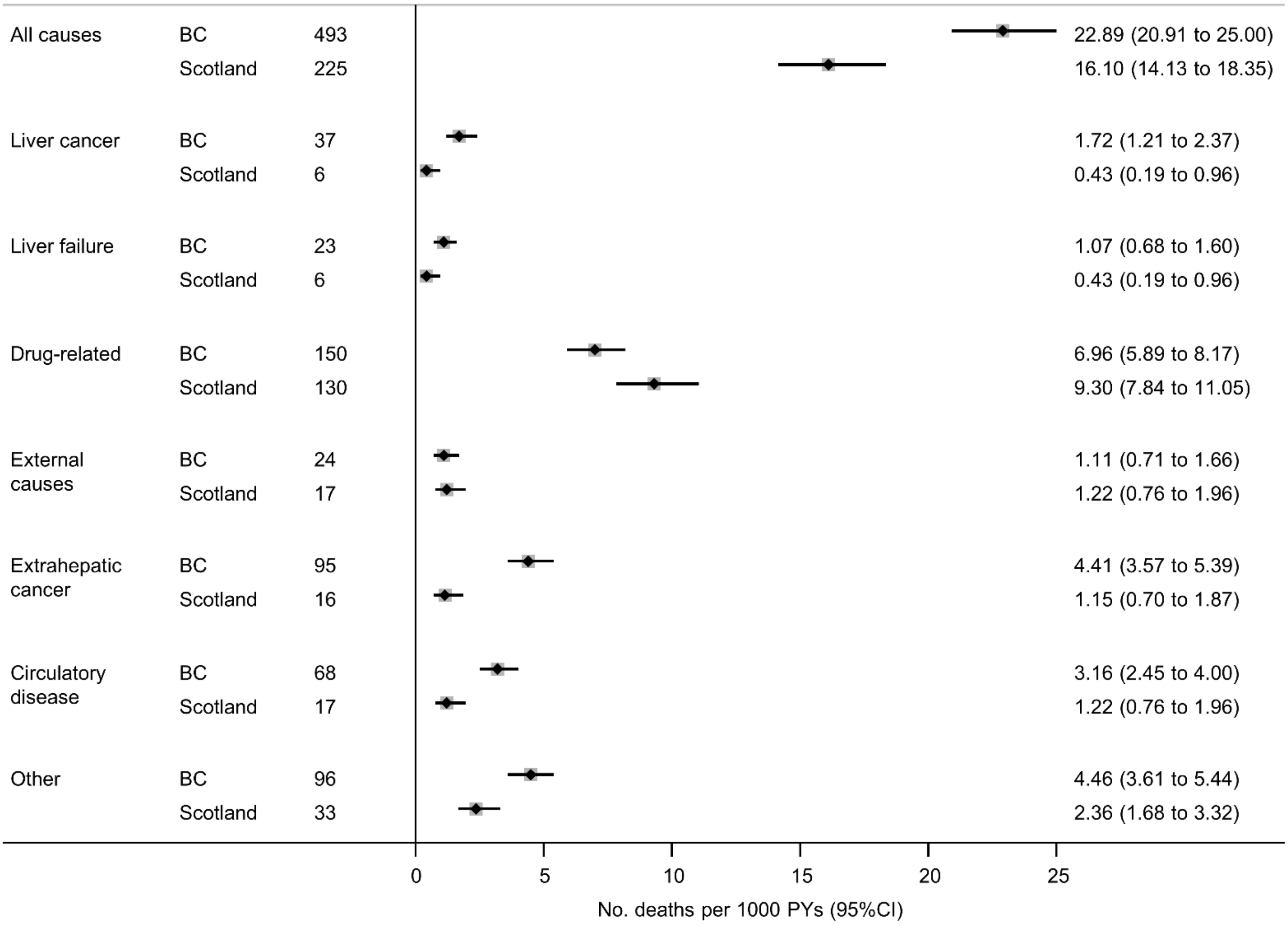

N.B. mortality data for pre-cirrhosis patients were not available in our England cohort.
PYs = person years

Figure S7. Crude mortality rates for individuals with compensated cirrhosis, by cause of death and setting

Cause of death	Setting/ Country	No. deaths	**Mortality rate:** No. deaths per 1000 PYs (95% CI)
All causes	BC	100	37.75 (30.72 to 45.92)
	Scotland	163	35.25 (30.24 to 41.10)
	England	120	23.54 (19.69 to 28.15)
Liver cancer	BC	19	7.17 (4.32 to 11.20)
	Scotland	26	5.62 (3.83 to 8.26)
	England	28	5.49 (3.79 to 7.96)
Liver failure	BC	19	7.17 (4.32 to 11.20)
	Scotland	19	4.11 (2.62 to 6.44)
	England	38	7.46 (5.42 to 10.25)
Drug-related	BC	13	4.91 (2.61 to 8.39)
	Scotland	54	11.68 (8.95 to 15.25)
	England	6	1.18 (0.53 to 2.62)
External causes	BC	4	1.51 (0.41 to 3.87)
	Scotland	9	1.95 (1.01 to 3.74)
	England	3	0.59 (0.19 to 1.82)
Extrahepatic cancer	BC	13	4.91 (2.61 to 8.39)
	Scotland	16	3.46 (2.12 to 5.65)
	England	11	2.16 (1.20 to 3.90)
Circulatory disease	BC	15	5.66 (3.17 to 9.34)
	Scotland	17	3.68 (2.29 to 5.91)
	England	13	2.55 (1.48 to 4.39)
Other	BC	17	6.42 (3.74 to 10.28)
	Scotland	22	4.76 (3.13 to 7.23)
	England	21	4.12 (2.69 to 6.32)

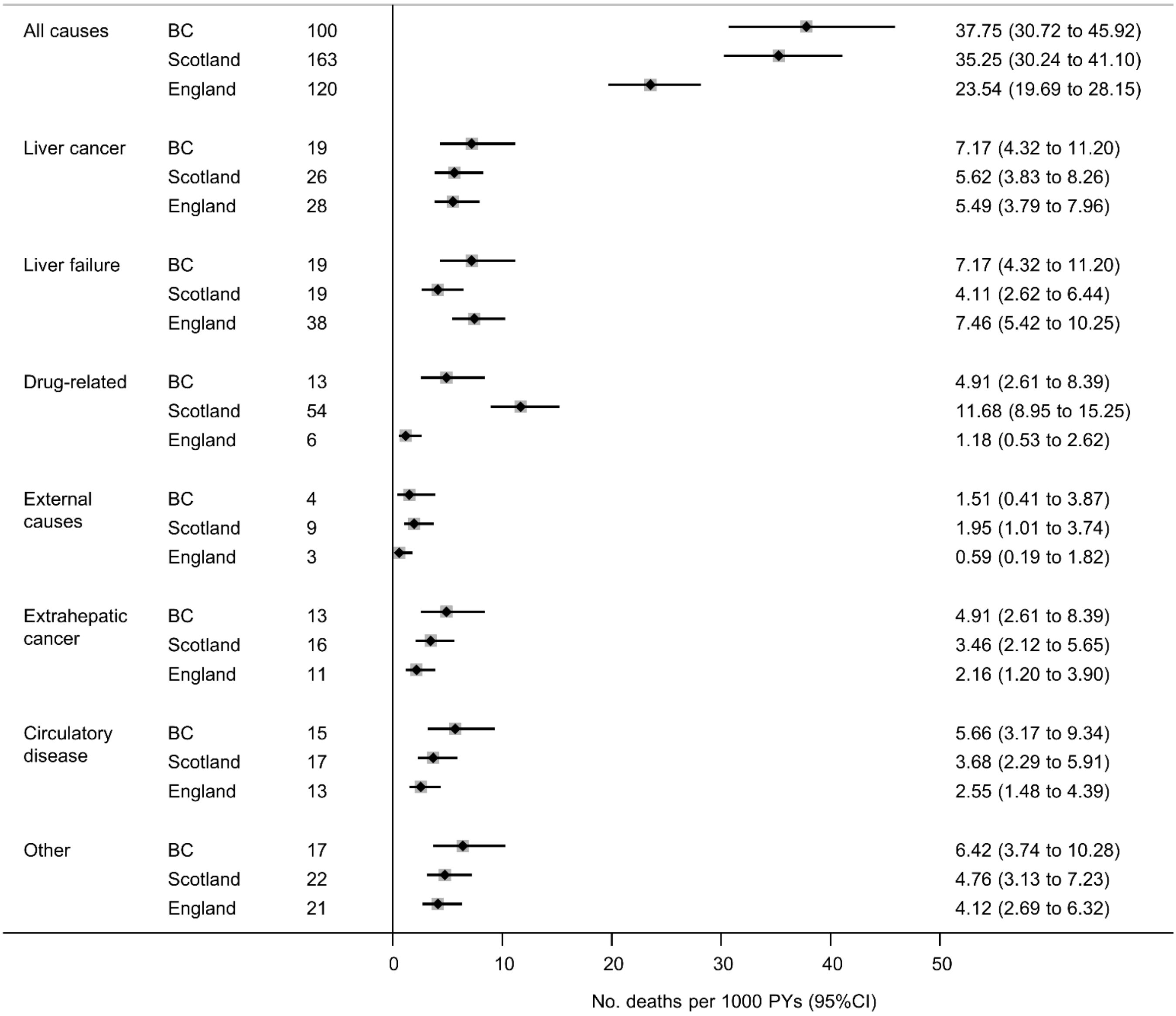

PYs = person years

Figure S8. Crude mortality rates for individuals with end stage liver disease, by cause of death and setting

Cause of death	Setting/ Country	No. deaths	**Mortality rate:** No. deaths per 1000 PYs (95% CI)
All causes	BC	228	118.18 (103.33 to 134.55)
	Scotland	54	63.56 (48.68 to 82.98)
	England	189	69.76 (60.49 to 80.45)
Liver cancer	BC	60	31.10 (23.73 to 40.03)
	Scotland	19	22.36 (14.26 to 35.06)
	England	55	20.30 (15.59 to 26.44)
Liver failure	BC	88	45.61 (36.58 to 56.19)
	Scotland	19	22.36 (14.26 to 35.06)
	England	74	27.31 (21.75 to 34.30)
Drug-related	BC	20	10.37 (6.33 to 16.01)
	Scotland	6	7.06 (3.17 to 15.72)
	England	4	1.48 (0.55 to 3.93)
External causes	BC	5	2.59 (0.84 to 6.05)
	Scotland	2	2.35 (0.59 to 9.41)
	England	3	1.11 (0.36 to 3.43)
Extrahepatic cancer	BC	15	7.77 (4.35 to 12.82)
	Scotland	2	2.35 (0.59 to 9.41)
	England	13	4.80 (2.79 to 8.26)
Circulatory disease	BC	21	10.88 (6.74 to 16.64)
	Scotland	2	2.35 (0.59 to 9.41)
	England	23	8.49 (5.64 to 12.78)
Other	BC	19	9.85 (5.93 to 15.38)
	Scotland	4	4.71 (1.77 to 12.54)
	England	17	6.27 (3.90 to 10.09)

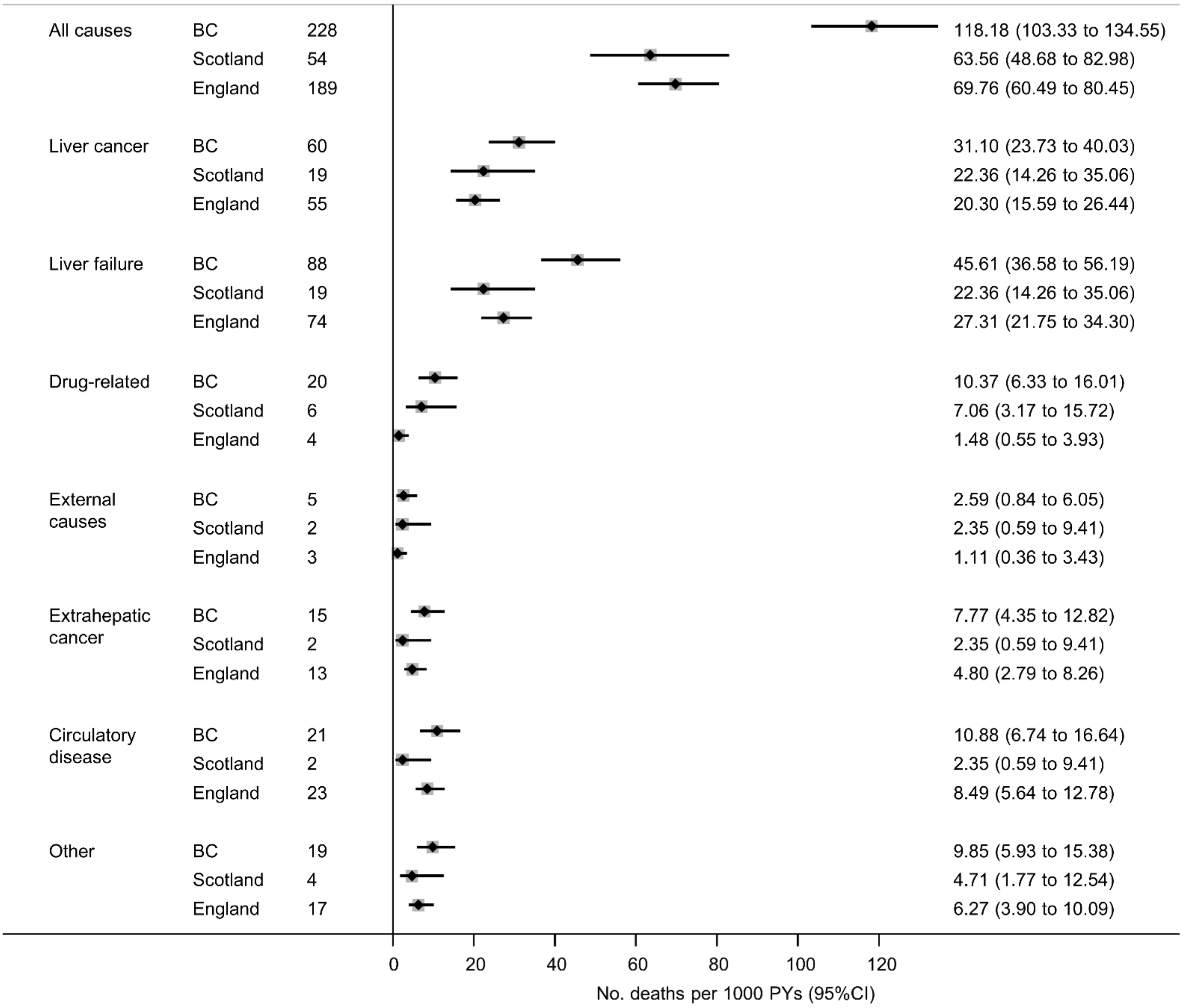

PYs = person years

Figure S9. Standardised mortality rates for pre-cirrhosis individuals, by cause of death and setting

Cause of death	Setting/ Country	No. deaths	**Mortality rate:** No. deaths per 1000 PYs (95% CI)
All causes	BC	493	22.89 (20.91 to 25.00)
	Scotland	225	20.14 (15.65 to 24.64)
Liver cancer	BC	37	1.72 (1.21 to 2.37)
	Scotland	6	1.78 (0.14 to 3.41)
Liver failure	BC	23	1.07 (0.68 to 1.60)
	Scotland	6	0.59 (0.00 to 1.36)
Drug-related	BC	150	6.96 (5.89 to 8.17)
	Scotland	130	6.28 (4.24 to 8.32)
External causes	BC	24	1.11 (0.71 to 1.66)
	Scotland	17	0.67 (0.01 to 1.32)
Extrahepatic cancer	BC	95	4.41 (3.57 to 5.39)
	Scotland	16	2.98 (1.00 to 4.96)
Circulatory disease	BC	68	3.16 (2.45 to 4.00)
	Scotland	17	1.92 (0.65 to 3.18)
Other	BC	96	4.46 (3.61 to 5.44)
	Scotland	33	5.94 (3.22 to 8.65)

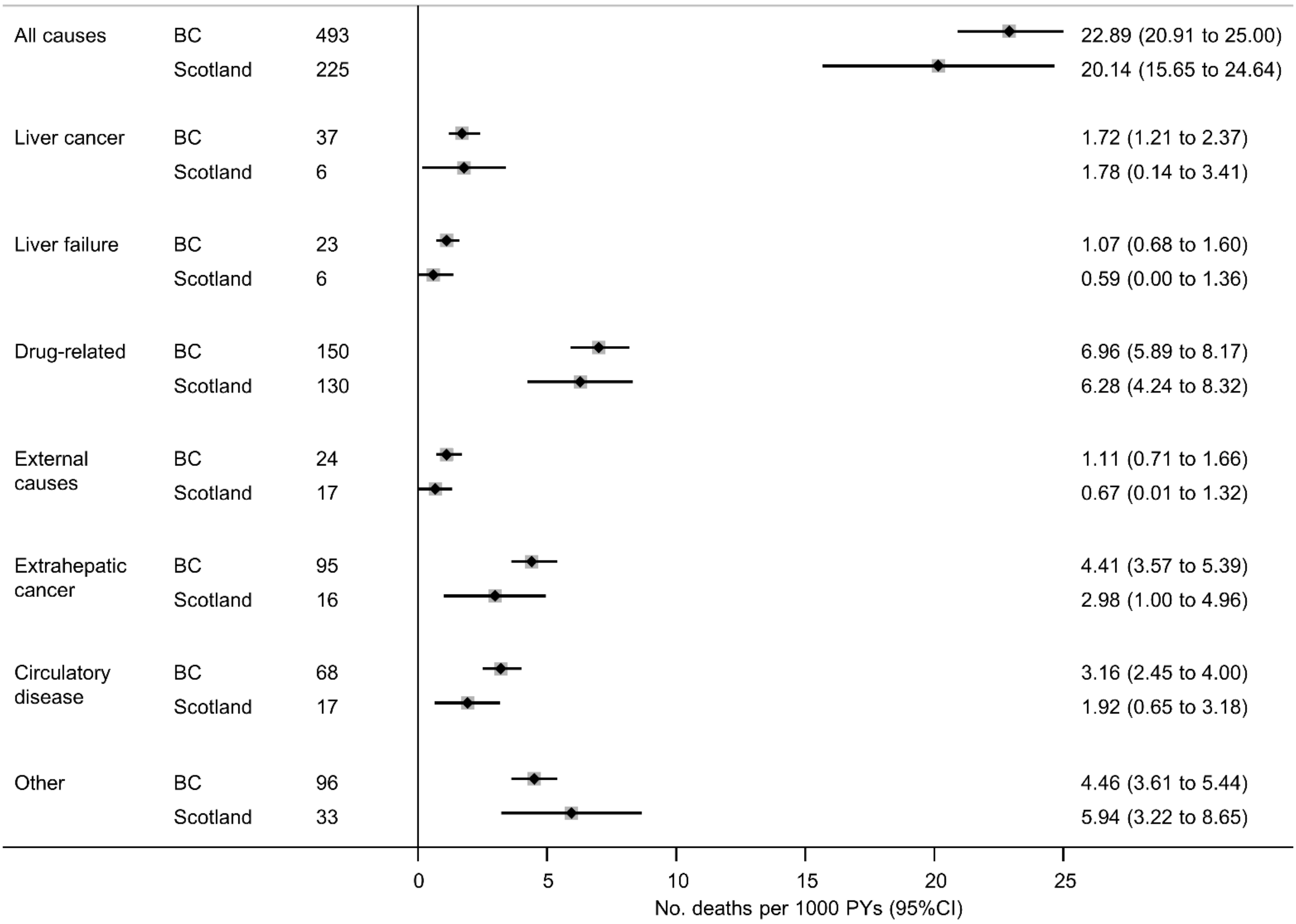

Mortality rates are standardized for age and sex, using pre-cirrhosis patients from BC as the standard population. See Table S12 for further details.

N.B. mortality data for pre-cirrhosis patients were not available in our England cohort.

PYs=person years

Figure S10. Standardised mortality rates for individuals with compensated cirrhosis, by cause of death and setting

Cause of death	Setting/ Country	No. deaths	**Mortality rate:** No. deaths per 1000 PYs (95% CI)
All causes	BC	100	37.75 (30.72 to 45.92)
	Scotland	163	37.33 (29.16 to 45.49)
	England	120	25.73 (20.82 to 30.65)
Liver cancer	BC	19	7.17 (4.32 to 11.20)
	Scotland	26	9.20 (4.78 to 13.62)
	England	28	6.35 (3.88 to 8.83)
Liver failure	BC	19	7.17 (4.32 to 11.20)
	Scotland	19	4.96 (1.90 to 8.02)
	England	38	7.93 (5.19 to 10.67)
Drug-related	BC	13	4.91 (2.61 to 8.39)
	Scotland	54	4.85 (2.95 to 6.75)
	England	6	0.87 (0.08 to 1.67)
External causes	BC	4	1.51 (0.41 to 3.87)
	Scotland	9	0.92 (0.12 to 1.73)
	England	3	0.52 (0.00 to 1.22)
Extrahepatic cancer	BC	13	4.91 (2.61 to 8.39)
	Scotland	16	6.32 (2.68 to 9.95)
	England	11	2.89 (1.12 to 4.66)
Circulatory disease	BC	15	5.66 (3.17 to 9.34)
	Scotland	17	4.47 (1.52 to 7.43)
	England	13	2.82 (1.15 to 4.50)
Other	BC	17	6.42 (3.74 to 10.28)
	Scotland	22	6.60 (2.90 to 10.31)
	England	21	4.34 (2.35 to 6.33)

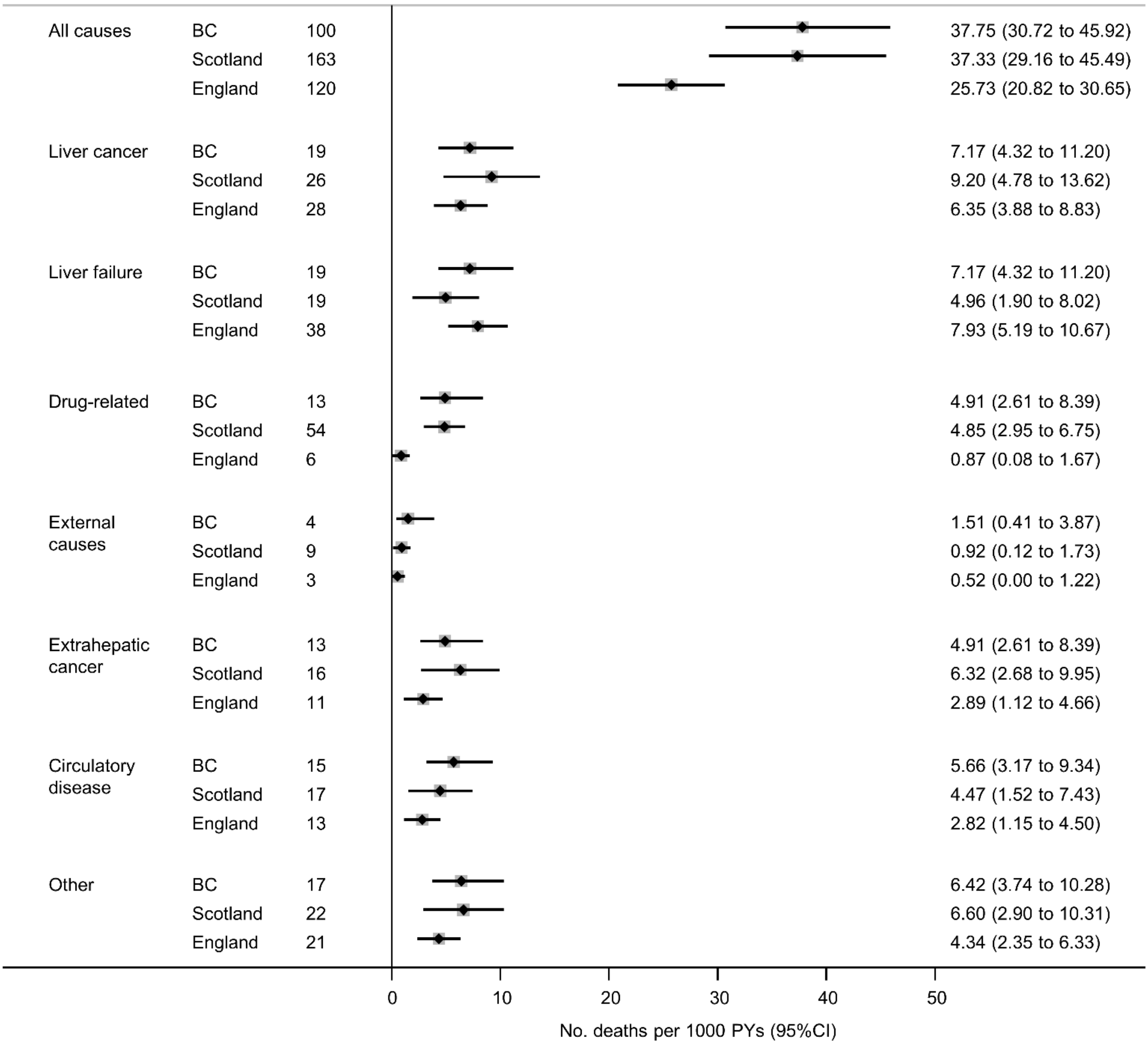

Mortality rates are standardized for age and sex, using compensated cirrhosis patients from BC as the standard population. See Table S12 for further details.

PYs = person years

Figure S11. Standardised mortality rates for individuals with ESLD, by cause of death and setting

Cause of death	Setting/ Country	No. deaths	**Mortality rate:** No. deaths per 1000 PYs (95% CI)
All causes	BC	228	118.18 (103.33 to 134.55)
	Scotland	54	64.84 (43.22 to 86.46)
	England	189	68.36 (58.03 to 78.70)
Liver cancer	BC	60	31.10 (23.73 to 40.03)
	Scotland	19	32.95 (15.60 to 50.31)
	England	55	19.84 (14.08 to 25.59)
Liver failure	BC	88	45.61 (36.58 to 56.19)
	Scotland	19	15.71 (6.24 to 25.17)
	England	74	27.10 (20.50 to 33.69)
Drug-related	BC	20	10.37 (6.33 to 16.01)
	Scotland	6	5.19 (0.00 to 10.48)
	England	4	0.96 (0.00 to 2.13)
External causes	BC	5	2.59 (0.84 to 6.05)
	Scotland	2	1.96 (0.00 to 5.05)
	England	3	0.94 (0.00 to 2.03)
Extrahepatic cancer	BC	15	7.77 (4.35 to 12.82)
	Scotland	2	2.88 (0.00 to 7.24)
	England	13	5.37 (2.19 to 8.55)
Circulatory disease	BC	21	10.88 (6.74 to 16.64)
	Scotland	2	2.93 (0.00 to 7.37)
	England	23	8.41 (4.65 to 12.18)
Other	BC	19	9.85 (5.93 to 15.38)
	Scotland	4	3.22 (0.00 to 7.52)
	England	17	5.75 (2.73 to 8.76)

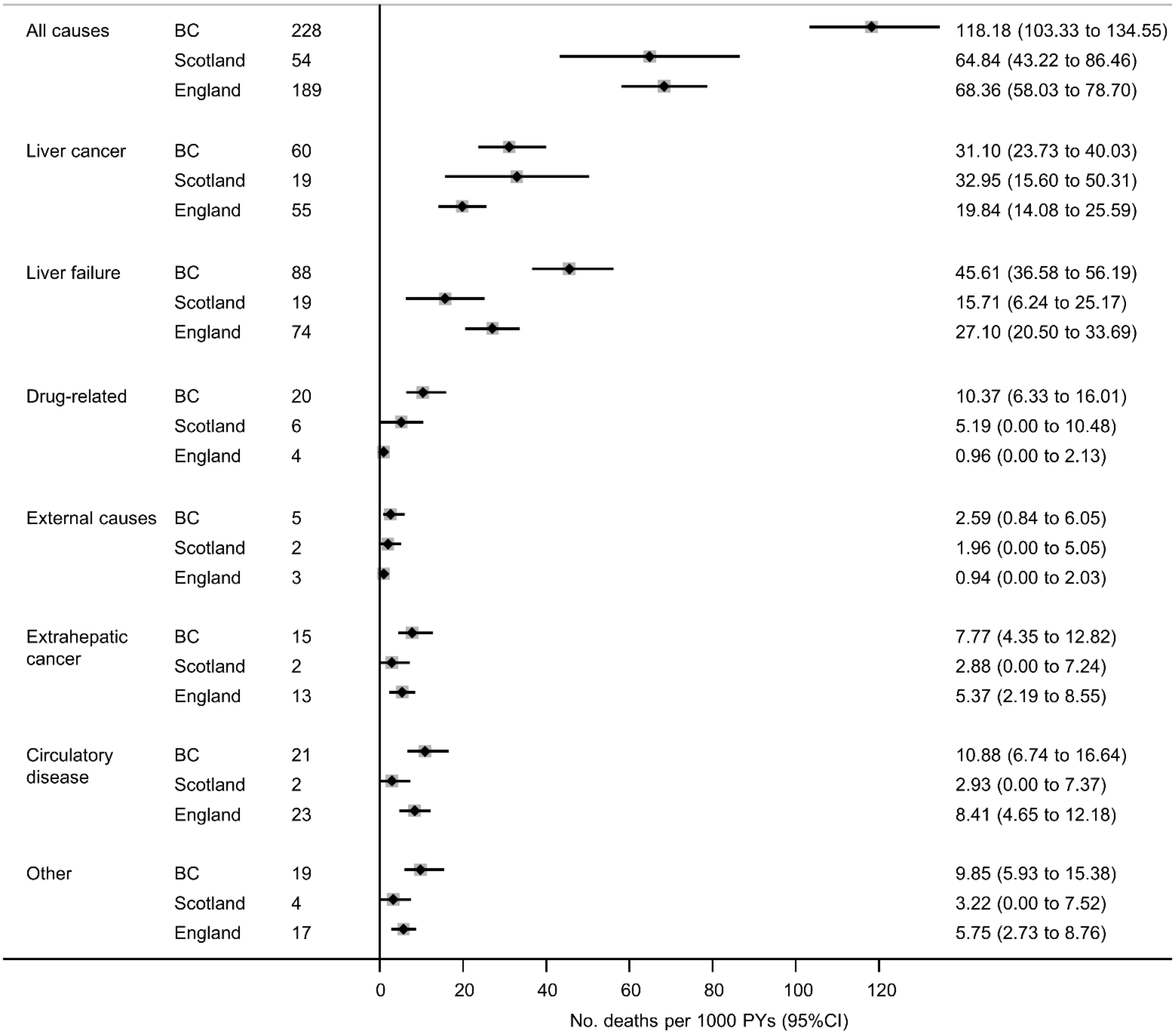

Mortality rates are standardized for age and sex, using ESLD patients from BC as the standard population. See Table S12 for further details.

ESLD refers to End stage liver disease; PYs = person years

Figure S12. Standardised mortality ratio (SMR) for pre-cirrhosis individuals, by cause of death and setting

Setting/ Country	Cause of death	Observed No. deaths	Expected No. deaths*	SMR (95% CI) †
BC	All causes	493	166.6	2.96 (2.71 to 3.23)
	Liver cancer	37	4.2	8.92 (6.46 to 12.31)
	Liver failure	23	6.5	3.52 (2.34 to 5.29)
	Drug-related	150	5.3	28.48 (24.27 to 33.42)
	External causes	24	7.8	3.07 (2.06 to 4.59)
	Extrahepatic cancer	95	57.5	1.65 (1.35 to 2.02)
	Circulatory disease	68	34.9	1.95 (1.53 to 2.47)
	Other	96	50.4	1.90 (1.56 to 2.33)
Scotland	All causes	225	59.4	3.79 (3.30 to 4.34)
	Liver cancer	6	0.7	8.89 (4.00 to 19.79)
	Liver failure	6	3.4	1.78 (0.80 to 3.97)
	Drug-related	130	7.4	17.50 (14.73 to 20.78)
	External causes	17	5.2	3.24 (2.01 to 5.21)
	Extrahepatic cancer	16	16.2	0.99 (0.60 to 1.61)
	Circulatory disease	17	12.7	1.34 (0.83 to 2.16)
	Other	33	13.8	2.40 (1.69 to 3.40)

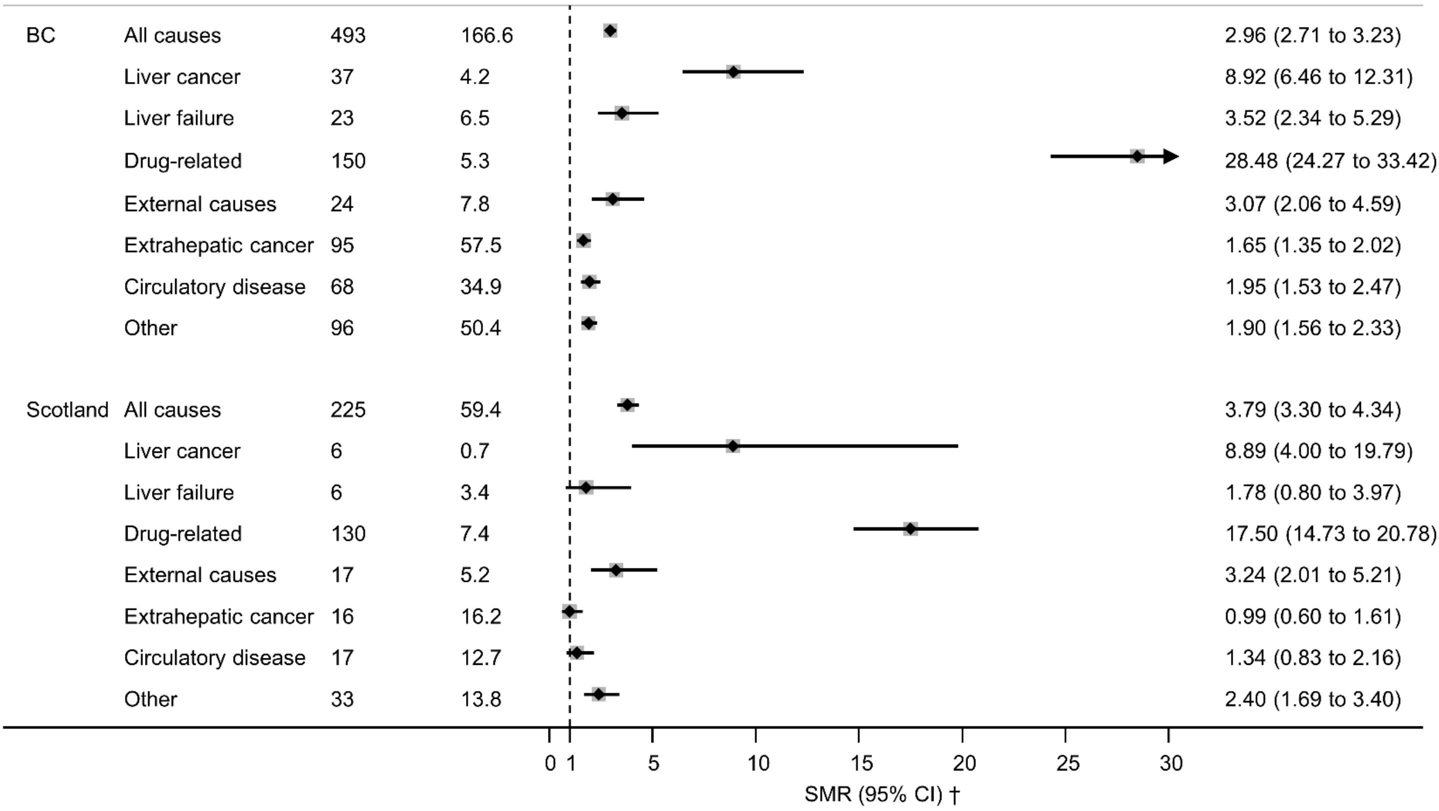

*Expected deaths indicates the number of deaths that would have occurred if cured patients exhibited the same age/sex/year specific mortality rates as the corresponding general population.

† SMR is the ratio of observed to expected deaths. Values above 1 indicate excess mortality (i.e. no. deaths observed exceed no. deaths expected).

N.B. mortality data for pre-cirrhosis patients were not available in the England cohort.

Figure S13. Standardised mortality ratio (SMR) for individuals with compensated cirrhosis, by cause of death and setting.

Setting/ Country	Cause of death	Observed No. deaths	Expected No. deaths*	SMR (95% CI) †
BC	All causes	100	25.2	3.96 (3.26 to 4.82)
	Liver cancer	19	0.6	30.30 (19.33 to 47.50)
	Liver failure	19	0.9	20.33 (12.97 to 31.87)
	Drug-related	13	0.6	21.09 (12.24 to 36.32)
	External causes	4	1.1	3.80 (1.43 to 10.13)
	Extrahepatic cancer	13	8.9	1.46 (0.85 to 2.51)
	Circulatory disease	15	5.4	2.76 (1.67 to 4.58)
	Other	17	7.6	2.22 (1.38 to 3.58)
Scotland	All causes	163	31.3	5.21 (4.39 to 6.17)
	Liver cancer	26	0.4	60.96 (41.47 to 89.62)
	Liver failure	19	1.6	11.75 (7.49 to 18.43)
	Drug-related	54	2.1	26.32 (20.14 to 34.38)
	External causes	9	1.9	4.67 (2.43 to 9.00)
	Extrahepatic cancer	16	9.7	1.65 (1.01 to 2.70)
	Circulatory disease	17	7.5	2.28 (1.41 to 3.70)
	Other	22	8.1	2.71 (1.75 to 4.18)
England	All causes	120	39.7	3.02 (2.51 to 3.65)
	Liver cancer	28	0.6	44.91 (30.99 to 65.10)
	Liver failure	38	1.6	23.29 (16.96 to 32.00)
	Drug-related	6	0.6	10.90 (4.89 to 24.29)
	External causes	3	1.7	1.80 (0.58 to 5.58)
	Extrahepatic cancer	11	14.6	0.75 (0.41 to 1.36)
	Circulatory disease	13	9.8	1.32 (0.77 to 2.28)
	Other	21	10.7	1.96 (1.26 to 3.03)

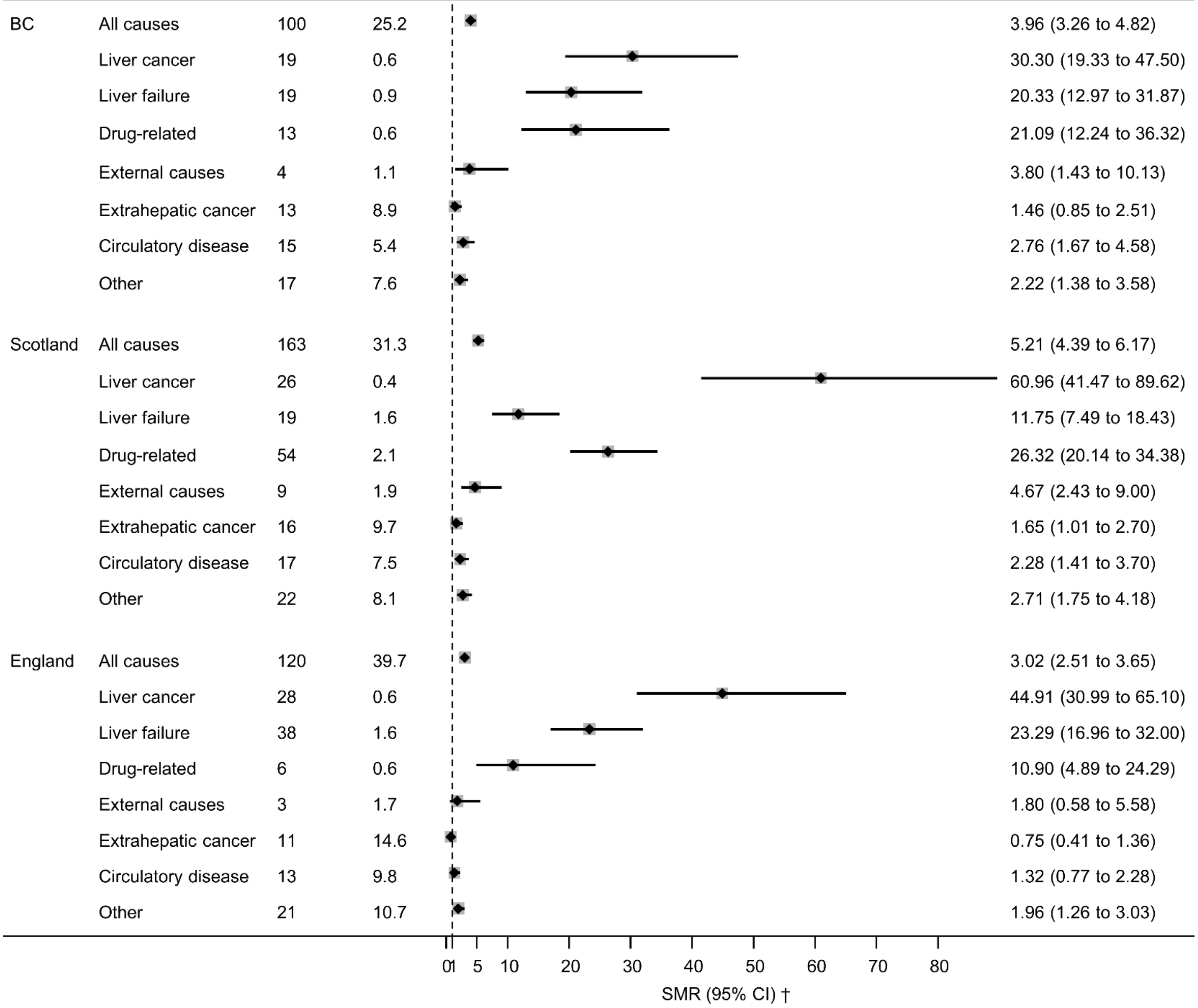

*Expected deaths indicates the number of deaths that would have occurred if cured patients exhibited the same age/sex/year specific mortality rates as the corresponding general population.

† SMR is the ratio of observed to expected deaths. Values above 1 indicate excess mortality (i.e. no. deaths observed exceed no. deaths expected).

Figure S14. Standardised mortality ratio (SMR) for individuals with end-stage-liver-disease, by cause of death and setting

Setting/ Country	Cause of death	Observed No. deaths	Expected No. deaths*	SMR (95% CI) †
BC	All causes	228	16.8	13.61 (11.95 to 15.49)
	Liver cancer	60	0.5	134.16 (104.17 to 172.78)
	Liver failure	88	0.7	130.92 (106.23 to 161.34)
	Drug-related	20	0.4	45.60 (29.42 to 70.68)
	External causes	5	0.7	7.00 (2.91 to 16.82)
	Extrahepatic cancer	15	6.1	2.45 (1.48 to 4.06)
	Circulatory disease	21	3.5	6.06 (3.95 to 9.30)
	Other	19	4.9	3.88 (2.47 to 6.08)
Scotland	All causes	54	6.8	7.89 (5.83 to 10.68)
	Liver cancer	19	0.1	195.07 (123.84 to 210.00)
	Liver failure	19	0.3	60.88 (38.71 to 95.74)
	Drug-related	6	0.3	19.38 (8.71 to 43.10)
	External causes	2	0.3	5.89 (1.47 to 23.55)
	Extrahepatic cancer	2	2.3	0.89 (0.22 to 3.56)
	Circulatory disease	2	1.7	1.20 (0.29 to 4.91)
	Other	4	1.9	2.14 (0.78 to 5.91)
England	All causes	189	21.9	8.63 (7.34 to 10.15)
	Liver cancer	55	0.4	156.21 (118.97 to 205.10)
	Liver failure	74	0.9	81.02 (64.23 to 102.20)
	Drug-related	4	0.3	15.07 (5.65 to 40.18)
	External causes	3	0.9	3.39 (1.09 to 10.53)
	Extrahepatic cancer	13	8.2	1.58 (0.92 to 2.73)
	Circulatory disease	23	5.4	4.22 (2.80 to 6.37)
	Other	17	5.8	2.92 (1.78 to 4.78)

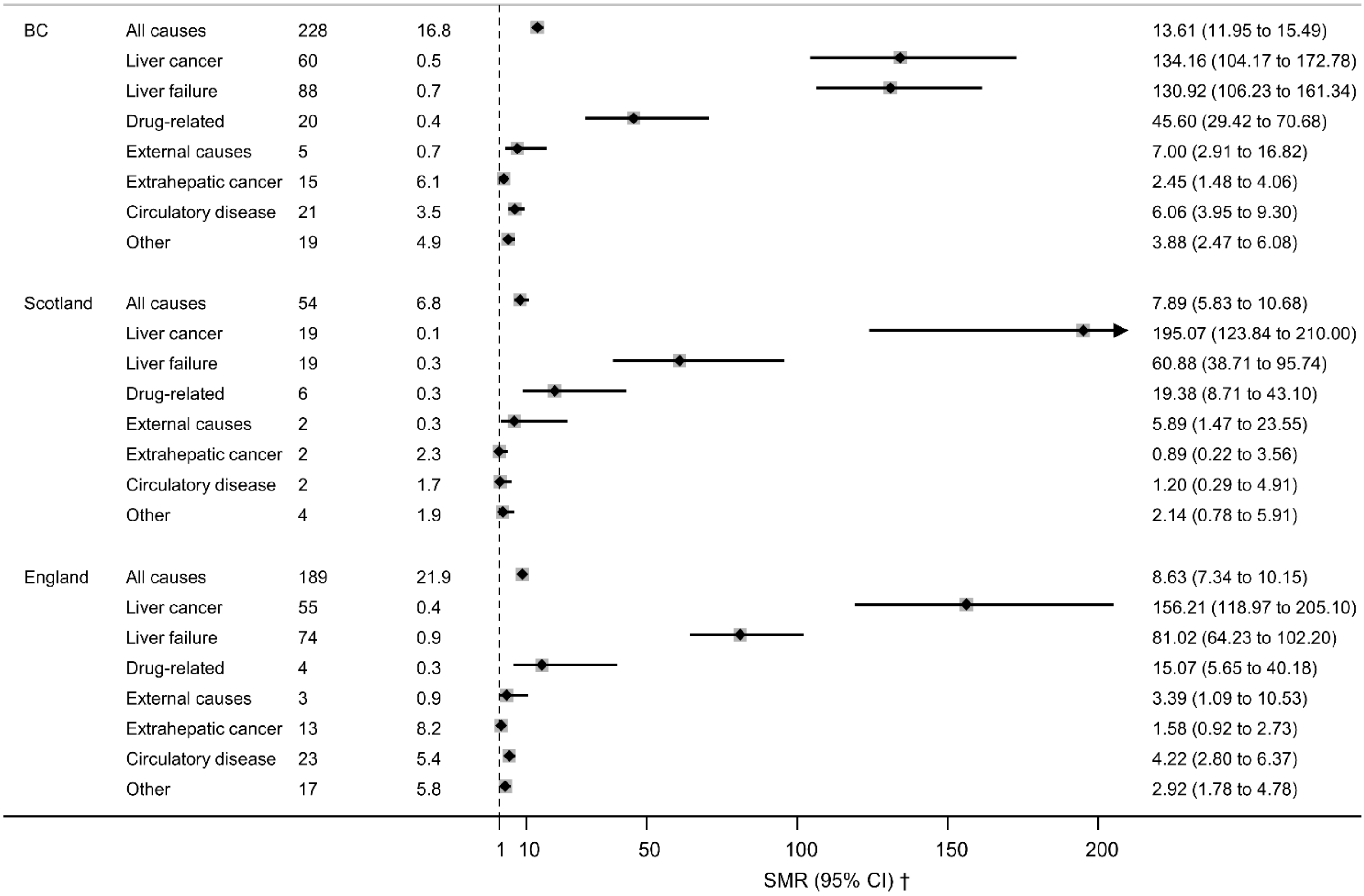

*Expected deaths indicates the number of deaths that would have occurred if cured patients exhibited the same age/sex/year specific mortality rates as the corresponding general population.

† SMR is the ratio of observed to expected deaths. Values above 1 indicate excess mortality (i.e. no. deaths observed exceed no. deaths expected). .

Figure S15. Standardised mortality ratios (SMR) for all-cause mortality in Scotland, with and without adjustment for area-based deprivation

Disease severity	Adjustment	Observed No. deaths	Expected No. deaths	SMR (95% CI)†	P-value ‡
Pre-cirrhosis	Age, sex, year	225	59.4	3.79 (3.30 to 4.34)	<0.001
	+SIMD*	224	81.7	2.74 (2.40 to 3.13)	<0.001
Cirrhosis	Age, sex, year	163	31.3	5.21 (4.39 to 6.17)	<0.001
	+SIMD*	163	41.8	3.90 (3.32 to 4.59)	<0.001
ESLD	Age, sex, year	54	6.8	7.89 (5.83 to 10.68)	<0.001
	+SIMD*	54	8.3	6.52 (4.91 to 8.65)	<0.001
All patients	Age, sex, year	442	97.6	4.53 (4.10 to 5.00)	<0.001
	+SIMD*	441	131.8	3.35 (3.04 to 3.68)	<0.001

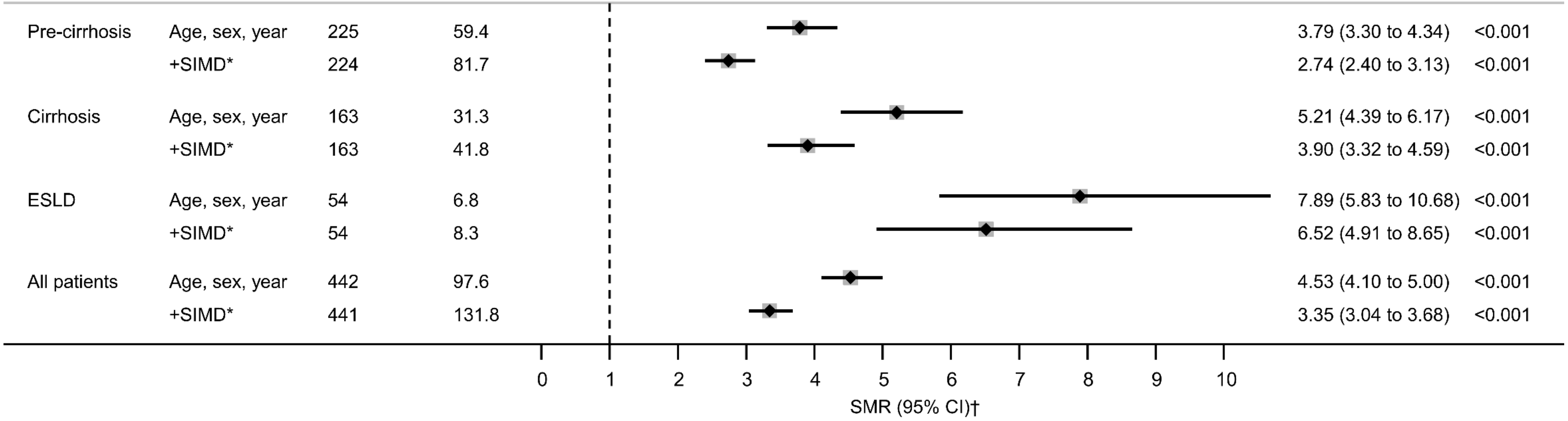

*SIMD = Scottish index multiple deprivation. A small fraction of individuals with missing SIMD data were excluded from the SIMD adjusted estimate.

† SMR is the ratio of observed to expected deaths. Values above 1 indicate excess mortality (i.e. no. deaths observed exceed no. deaths expected).

‡ Null hypothesis is that SMR =1 (represented by the dashed vertical line).

N.B. It was not possible to perform the equivalent adjustment in our BC or English cohort.

Figure S16. Non-linear relationship observed between Charlson Comorbidity Index (CCI) and mortality among pre-cirrhosis patients in British Columbia.

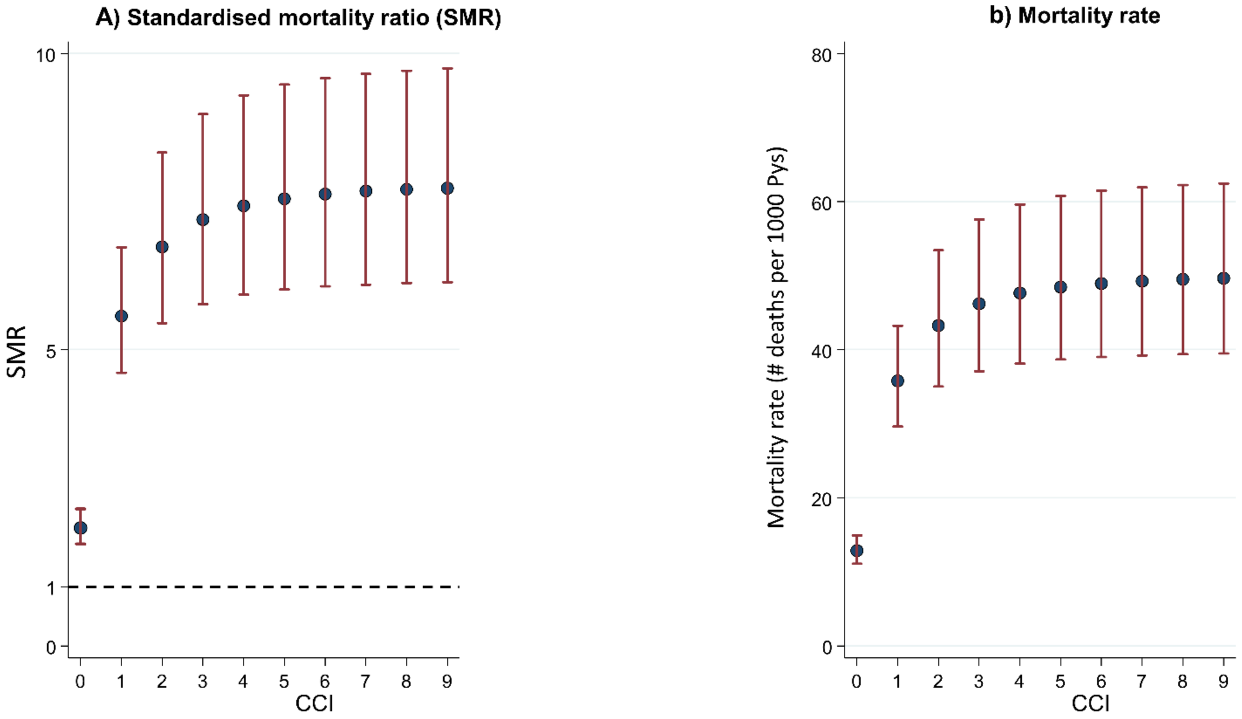

The modelled mortality rate and SMR in this figure are derived from the multivariate regression models shown in Table 2 and Table S17, respectively. The non-linear relationship represented by a first order fractional polynomial power term of -2.
Predicted estimates relate specifically to males, without a previous hospital admission for alcohol or substance use, with mean age and mean year of SVR achievement.

Table S1. All-cause mortality rate (deaths per 100,000) in the general population, by age group, year, sex and setting.

British Columbia

Year - Sex	Age group, years											
	10-19	20-29	30-39	40-44	45-49	50-54	55-59	60-64	65-69	70-74	75-79	80+
2014 M	30.0	80.9	104.5	144.3	230.4	341.6	586.4	879.9	1224.0	2142.9	3320.1	9152.8
2014 F	12.7	32.6	55.1	99.2	133.0	221.6	303.5	492.8	825.5	1292.9	2228.4	8213.9
2015 M	29.8	94.4	120.9	147.0	230.1	346.9	569.0	840.0	1220.6	1989.9	3213.4	9590.4
2015 F	15.2	32.9	46.9	84.3	117.7	196.9	337.6	537.3	783.2	1372.1	2294.4	8463.2
2016 M	32.0	114.7	149.0	196.1	254.1	361.1	542.2	865.3	1393.4	1931.3	3326.5	9505.9
2016 F	19.5	42.8	58.8	80.9	138.9	234.0	363.9	520.1	779.5	1301.2	2277.9	8333.1
2017 M	33.1	132.8	183.1	205.1	298.9	398.3	580.1	887.7	1325.4	2008.7	3312.4	9766.0
2017 F	22.0	42.7	66.5	94.4	154.7	229.3	327.3	480.3	773.7	1319.4	2241.4	8600.0
2018 M	38.0	127.3	180.3	223.3	272.5	412.3	608.6	900.6	1264.0	1961.5	3190.7	9281.6
2018 F	16.9	48.9	73.9	90.6	132.1	216.7	323.6	517.9	740.1	1204.1	2154.6	8359.2
2019 M	26.3	103.0	147.0	176.7	254.0	381.3	547.7	810.3	1275.2	1900.2	3106.0	9223.1
2019 F	21.1	42.4	66.7	98.3	163.4	199.8	320.7	489.0	729.8	1191.2	2223.2	8269.6

Scotland

Year - Sex	Age group, years											
	10-19	20-29	30-39	40-44	45-49	50-54	55-59	60-64	65-69	70-74	75-79	80+
2014 M	23.9	71.9	145.4	271.0	336.5	465.0	724.1	1146.6	1771.9	2980.6	4794.9	11620.0
2014 F	14.8	32.5	69.1	149.8	209.8	308.0	505.5	750.8	1176.4	1941.7	3482.9	10361.4
2015 M	24.4	70.3	160.0	260.9	355.5	481.7	743.8	1163.7	1862.2	3150.4	4901.9	12209.2
2015 F	11.0	29.7	87.8	143.0	229.1	326.3	484.0	719.5	1217.0	2100.4	3475.9	11099.2
2016 M	28.0	84.8	160.1	327.4	381.7	517.7	761.9	1149.6	1846.7	2915.9	4686.4	11769.3
2016 F	16.1	34.8	87.8	167.3	218.4	319.7	489.3	779.3	1170.9	2026.0	3486.0	10512.2
2017 M	24.7	73.9	175.8	279.0	400.1	503.6	714.5	1128.6	1779.2	2827.1	4975.4	12040.4
2017 F	16.5	27.9	90.0	161.3	205.4	332.0	497.2	764.8	1209.3	1955.9	3610.1	10841.6
2018 M	31.1	90.0	170.4	308.5	407.1	497.3	733.3	1118.0	1775.7	2668.4	4808.3	11680.3
2018 F	17.5	34.2	88.8	164.3	216.3	344.8	483.4	762.2	1204.6	1951.5	3431.0	10474.2
2019 M	26.5	87.4	171.4	333.6	408.4	508.9	682.0	1113.6	1714.0	2651.5	4588.9	11388.1
2019 F	16.7	38.3	88.1	186.3	233.6	316.2	492.7	777.7	1209.8	1861.2	3320.2	10321.3

England

Year - Sex	Age group, years											
	10-19	20-29	30-39	40-44	45-49	50-54	55-59	60-64	65-69	70-74	75-79	80+
2014 M	19.6	51.3	89.7	163.8	241.7	353.0	568.9	928.6	1400.3	2349.6	3930.0	10825.3
2014 F	11.5	23.7	53.3	105.4	155.3	241.1	376.1	603.3	909.6	1565.7	2709.4	9660.4
2015 M	19.9	54.2	93.6	168.7	247.3	369.8	577.1	940.8	1420.5	2337.2	4012.9	11242.4
2015 F	12.5	23.9	52.4	101.2	160.1	245.5	377.0	603.0	932.8	1560.3	2769.7	10282.3
2016 M	19.4	53.6	96.3	172.1	253.8	367.4	577.2	935.4	1449.6	2324.8	4038.7	11000.5
2016 F	12.2	25.3	58.3	107.1	164.5	252.3	394.3	614.8	948.3	1577.1	2780.1	9918.9
2017 M	21.3	54.4	97.7	175.0	260.7	375.2	569.9	919.1	1434.2	2268.0	3979.0	11002.7
2017 F	12.4	23.2	53.5	104.6	159.9	245.6	366.8	599.5	935.3	1503.3	2770.7	9996.9
2018 M	19.4	57.3	99.1	170.7	257.0	383.3	570.4	896.7	1435.1	2220.2	3862.7	10779.2
2018 F	11.9	26.0	55.9	101.5	169.5	241.7	383.5	592.8	922.3	1491.3	2692.4	9799.4
2019 M	19.6	54.1	100.1	166.1	260.7	380.9	563.5	874.5	1413.2	2174.9	3758.1	10534.5
2019 F	10.6	24.1	55.6	100.9	162.7	245.4	367.9	583.2	896.1	1464.6	2583.0	9397.2

Table S2. Mortality rate for primary liver cancer (deaths per 100,000) in the general population, by age group, year, sex and setting.

British Columbia

Year - Sex	Age group, years											
	10-19	20-29	30-39	40-44	45-49	50-54	55-59	60-64	65-69	70-74	75-79	80+
2014 M	0.0	0.3	0.3	0.0	0.0	5.4	26.7	38.9	32.3	45.7	66.0	68.1
2014 F	0.0	0.0	0.0	0.0	1.2	2.6	5.6	7.7	11.4	20.1	22.8	49.0
2015 M	0.0	0.3	0.9	2.0	5.4	8.1	20.5	43.1	39.4	50.3	24.4	70.8
2015 F	0.0	0.0	0.0	0.0	1.7	2.6	3.8	8.0	12.9	30.4	34.6	37.7
2016 M	0.0	0.3	0.9	1.3	4.2	7.7	13.9	40.5	46.3	41.8	38.3	74.4
2016 F	0.0	0.0	0.0	1.3	1.1	4.8	3.7	5.9	10.9	21.3	21.3	38.6
2017 M	0.0	0.6	1.2	0.7	2.4	7.9	15.4	34.7	40.8	45.1	70.3	74.4
2017 F	0.0	0.0	0.3	1.3	0.0	2.7	5.8	10.3	10.0	16.8	14.1	57.8
2018 M	0.0	0.0	0.3	1.3	1.8	5.2	14.2	35.6	43.7	39.7	55.8	66.8
2018 F	0.0	0.0	0.3	0.6	1.2	2.8	6.8	10.0	14.3	17.4	23.1	44.2
2019 M	0.0	0.0	0.6	1.3	1.3	4.2	15.2	30.3	48.9	39.4	49.0	66.6
2019 F	0.0	0.0	0.6	1.9	1.2	3.4	6.2	11.5	15.8	15.7	27.7	45.6

Scotland

Year - Sex	Age group, years											
	10-19	20-29	30-39	40-44	45-49	50-54	55-59	60-64	65-69	70-74	75-79	80+
2014 M	0.0	0.0	0.6	0.6	2.0	3.1	15.0	20.1	41.3	56.5	61.0	106.4
2014 F	0.0	0.3	0.0	1.6	2.9	3.4	5.0	5.6	12.7	21.9	36.4	47.6
2015 M	0.0	0.6	0.3	1.2	4.2	10.1	12.4	17.5	39.6	51.0	60.5	113.3
2015 F	0.0	0.0	0.0	0.6	1.0	2.9	8.1	8.6	19.2	23.3	38.1	54.3
2016 M	0.0	0.0	0.3	1.2	3.7	8.1	17.7	23.0	40.4	51.0	76.4	99.5
2016 F	0.0	0.3	0.0	0.6	0.5	5.7	5.3	10.3	13.4	23.6	44.1	45.9
2017 M	0.0	0.3	0.6	0.0	2.7	7.6	14.1	18.8	36.6	45.8	89.7	116.3
2017 F	0.0	0.0	0.0	0.6	3.5	2.9	6.7	12.4	19.7	27.0	44.0	57.2
2018 M	0.3	0.3	0.3	0.6	5.0	4.6	11.2	24.5	36.6	54.5	71.7	111.4
2018 F	0.0	0.0	0.0	1.2	1.0	1.9	5.0	10.4	17.4	18.9	25.5	46.9
2019 M	0.0	0.0	0.3	0.0	2.9	6.2	10.5	26.4	33.9	53.7	74.0	107.9
2019 F	0.0	0.3	0.0	0.6	2.1	2.9	6.9	8.4	11.6	19.1	35.2	50.9

England

Year - Sex	Age group, years											
	10-19	20-29	30-39	40-44	45-49	50-54	55-59	60-64	65-69	70-74	75-79	80+
2014 M	0.1	0.1	0.4	1.2	2.6	4.9	10.8	16.5	25.7	33.7	47.8	74.5
2014 F	0.0	0.1	0.2	0.6	1.0	2.1	5.0	7.7	13.1	19.8	28.7	44.3
2015 M	0..0	0.1	0.4	0.9	2.0	5.2	9.0	18.2	24.9	37.1	51.3	69.3
2015 F	0.1	0.1	0.2	0.7	1.4	2.7	4.5	6.8	11.5	18.1	27.9	46.5
2016 M	0.1	0.2	0.3	1.1	2.4	5.7	10.5	18.0	27.0	35.8	53.9	72.8
2016 F	0.0	0.1	0.3	0.4	0.9	3.1	4.4	8.9	12.6	21.5	27.9	43.5
2017 M	0.1	0.1	0.5	1.4	2.4	5.7	11.3	18.2	27.4	38.5	56.4	76.5
2017 F	0.0	0.1	0.3	0.8	1.3	2.7	4.9	10.5	13.5	18.0	32.1	43.6
2018 M	0.0	0.1	0.3	1.2	2.2	5.5	9.8	18.7	29.3	38.6	55.8	78.4
2018 F	0.0	0.1	0.3	0.5	1.5	2.1	4.5	8.9	14.2	19.9	28.9	42.9
2019 M	0.1	0.1	0.5	0.9	2.2	5.1	10.4	19.3	28.6	41.8	53.7	76.3
2019 F	0.0	0.1	0.5	0.9	1.7	2.6	5.5	7.6	14.7	21.9	28.9	47.9

Table S3. Mortality rate for liver failure (deaths per 100,000) in the general population, by age group, year, sex and setting.

British Columbia

Year - Sex	Age group, years											
	10-19	20-29	30-39	40-44	45-49	50-54	55-59	60-64	65-69	70-74	75-79	80+
2014 M	0.0	0.9	1.9	5.8	10.8	25.6	42.4	60.7	57.6	42.3	45.6	33.4
2014 F	0.0	0.3	2.2	4.3	8.7	15.3	24.6	19.2	24.4	32.8	38.4	25.7
2015 M	0.0	1.2	3.2	8.5	15.1	28.1	32.4	60.4	49.1	57.7	41.1	46.4
2015 F	0.0	0.0	2.2	5.6	8.7	11.0	21.2	27.0	25.0	29.4	34.6	21.3
2016 M	0.0	0.3	3.7	5.9	12.0	27.9	29.0	43.0	62.0	45.9	33.9	42.8
2016 F	0.0	0.3	3.0	5.0	8.5	16.9	27.6	27.1	23.1	38.6	21.3	26.6
2017 M	0.0	0.6	2.1	7.3	8.5	24.1	27.6	42.6	58.4	46.0	33.7	47.0
2017 F	0.0	0.9	3.9	3.2	8.0	16.2	15.8	18.9	25.3	28.4	29.5	27.7
2018 M	0.0	1.1	3.2	9.9	13.5	27.8	32.7	53.5	60.3	45.0	41.2	48.8
2018 F	0.0	0.9	2.9	6.3	6.9	11.6	19.8	24.0	25.4	21.5	31.7	25.6
2019 M	0.0	0.3	2.8	10.3	16.8	22.6	29.8	39.7	54.4	54.5	47.8	46.1
2019 F	0.4	1.2	3.9	3.7	12.9	14.6	15.5	20.8	27.2	32.9	24.2	29.6

Scotland

Year - Sex	Age group, years											
	10-19	20-29	30-39	40-44	45-49	50-54	55-59	60-64	65-69	70-74	75-79	80+
2014 M	0.0	1.1	10.9	23.4	31.2	39.1	56.6	55.2	65.7	61.2	35.4	43.9
2014 F	0.0	0.6	5.7	13.5	20.2	20.6	31.9	26.6	25.3	18.6	32.6	27.0
2015 M	0.3	0.6	7.9	18.2	39.7	41.1	49.7	64.8	60.7	48.2	52.0	33.1
2015 F	0.0	0.5	5.9	12.3	22.0	23.6	35.5	25.2	32.2	37.8	29.6	27.5
2016 M	0.0	1.4	7.2	23.7	30.6	48.1	64.2	65.2	60.0	66.2	54.5	48.7
2016 F	0.0	0.8	5.8	13.9	17.8	28.2	26.3	29.7	31.7	29.1	43.1	27.1
2017 M	0.0	0.8	7.9	17.6	39.9	46.1	54.7	63.2	69.9	59.7	47.8	53.6
2017 F	0.0	0.8	4.2	11.5	18.5	22.4	34.5	26.6	36.2	40.1	23.9	26.7
2018 M	0.0	0.3	5.5	25.7	33.2	36.3	53.2	45.3	72.4	67.0	47.0	51.3
2018 F	0.0	0.8	5.0	11.1	17.6	22.5	31.7	34.7	29.0	35.1	33.9	33.3
2019 M	0.0	0.8	5.4	19.1	27.5	35.6	44.0	49.7	65.8	44.6	48.9	49.7
2019 F	0.0	0.3	3.8	12.9	14.5	22.2	36.1	40.5	37.4	33.4	30.5	34.5

England

Year - Sex	Age group, years											
	10-19	20-29	30-39	40-44	45-49	50-54	55-59	60-64	65-69	70-74	75-79	80+
2014 M	0.0	0.6	6.7	16.3	26.4	32.7	38.8	46.3	41.7	36.7	37.9	34.3
2014 F	0.0	0.4	4.3	9.8	14.2	17.5	20.7	22.0	20.1	23.0	25.6	20.9
2015 M	0.2	0.5	6.5	17.5	27.1	34.6	41.4	45.1	44.2	37.3	36.2	33.7
2015 F	0.0	0.4	4.7	10.4	14.1	17.2	21.9	25.2	22.9	23.6	25.4	22.7
2016 M	0.1	0.8	6.8	16.6	28.4	34.1	41.8	44.1	45.7	39.3	40.0	38.3
2016 F	0.0	0.7	3.9	10.3	15.6	17.6	20.9	24.4	21.5	24.0	26.4	23.2
2017 M	0.0	0.9	5.6	18.3	27.3	32.9	41.5	47.2	47.2	37.3	42.7	36.7
2017 F	0.0	0.5	4.5	11.2	14.2	17.6	22.5	24.8	23.0	20.9	26.5	23.3
2018 M	0.1	0.7	5.8	15.2	25.4	33.8	39.8	42.2	44.1	42.2	36.1	40.0
2018 F	0.0	0.5	3.9	8.3	14.1	17.2	19.7	25.9	23.8	23.7	25.4	22.5
2019 M	0.1	0.5	4.8	14.4	25.8	35.1	42.1	46.0	43.5	39.9	42.3	34.9
2019 F	0.0	0.4	3.8	9.7	15.2	18.8	21.3	23.7	24.5	24.4	23.8	24.1

Table S4. Drug-related mortality rate (deaths per 100,000) in the general population, by age group, year, sex and setting.

British Columbia

Year - Sex	Age group, years											
	10-19	20-29	30-39	40-44	45-49	50-54	55-59	60-64	65-69	70-74	75-79	80+
2014 M	2.3	19.3	27.3	23.0	28.9	27.2	23.8	20.5	10.3	2.2	9.4	7.2
2014 F	0.4	8.9	13.0	10.4	15.7	10.6	15.1	9.0	7.6	4.2	4.3	7.5
2015 M	3.4	23.9	37.2	33.8	32.6	31.4	35.8	26.4	11.2	4.3	6.1	11.6
2015 F	1.2	11.4	10.6	15.5	14.4	18.4	9.2	8.0	4.3	4.1	2.8	5.7
2016 M	4.6	46.4	68.2	70.7	64.3	63.9	49.0	21.2	18.5	3.1	1.5	10.1
2016 F	5.7	14.5	16.7	14.4	19.9	23.2	20.7	13.5	6.1	2.9	5.3	1.6
2017 M	7.2	63.9	102.8	82.7	109.5	90.9	60.1	54.8	16.9	8.5	5.6	3.3
2017 F	6.8	14.7	21.1	22.7	25.1	18.9	18.4	14.3	6.0	2.7	2.6	4.8
2018 M	7.5	41.6	62.8	55.2	58.2	71.3	54.0	31.5	16.0	8.8	6.6	3.2
2018 F	2.4	15.0	18.5	12.0	20.8	18.2	15.1	11.2	3.3	2.5	7.3	3.1
2019 M	1.1	9.2	18.0	14.8	14.9	16.1	10.3	12.8	10.2	4.2	2.5	4.1
2019 F	4.3	4.8	7.0	8.1	7.6	3.9	10.3	7.1	2.5	3.1	3.5	1.5

Scotland

Year - Sex	Age group, years											
	10-19	20-29	30-39	40-44	45-49	50-54	55-59	60-64	65-69	70-74	75-79	80+
2014 M	3.4	24.7	50.5	58.8	36.8	32.9	10.4	5.2	4.7	5.7	2.4	0.0
2014 F	1.8	6.8	17.9	17.2	14.0	10.8	8.8	5.0	3.8	2.4	1.9	0.6
2015 M	1.7	18.1	51.6	52.9	42.8	26.9	16.4	7.8	7.9	3.7	6.1	1.1
2015 F	0.7	4.4	24.1	26.7	21.5	17.3	11.3	11.1	2.5	0.8	5.7	1.9
2016 M	3.8	22.6	61.2	91.0	51.2	27.9	15.5	10.2	9.1	0.9	1.2	4.1
2016 F	2.9	8.9	22.8	34.2	26.6	16.7	11.1	8.5	3.1	6.3	3.8	3.8
2017 M	2.7	22.5	64.3	83.4	67.4	41.0	17.3	10.6	8.1	4.9	3.6	1.0
2017 F	2.2	5.7	29.1	41.1	24.6	20.0	15.4	5.9	5.1	5.8	2.9	1.2
2018 M	6.2	32.1	72.0	113.8	98.0	55.7	22.3	13.5	7.6	3.1	4.7	3.0
2018 F	2.2	8.2	30.5	43.2	31.0	22.5	12.6	9.8	8.4	1.4	0.9	1.9
2019 M	4.8	30.0	67.3	119.9	107.0	63.9	26.7	15.0	4.2	3.8	5.7	2.9
2019 F	3.6	12.1	30.0	56.0	40.2	26.6	15.8	9.0	7.1	2.1	1.9	0.0

England

Year - Sex	Age group, years											
	10-19	20-29	30-39	40-44	45-49	50-54	55-59	60-64	65-69	70-74	75-79	80+
2014 M	0.8	7.5	16.9	19.0	15.6	10.8	7.4	5.9	3.3	2.8	3.0	3.6
2014 F	0.5	2.5	5.8	7.3	6.1	6.9	4.8	4.3	3.3	2.5	1.9	2.5
2015 M	0.8	7.9	19.1	22.8	19.1	13.2	9.2	5.9	3.8	3.0	1.9	3.7
2015 F	0.5	2.4	6.1	8.8	7.8	7.1	5.1	4.1	2.3	2.3	2.7	2.9
2016 M	0.8	8.2	19.3	23.5	18.9	12.3	9.6	7.3	3.0	2.7	2.4	3.7
2016 F	0.8	3.4	6.8	9.0	9.1	7.0	6.8	5.2	4.0	2.3	3.1	3.1
2017 M	1.0	8.2	17.9	25.5	21.7	16.0	9.8	6.2	4.8	2.2	2.2	3.9
2017 F	0.8	3.0	6.5	9.3	9.2	7.6	4.4	4.7	3.5	2.9	2.6	3.0
2018 M	1.1	10.0	20.3	26.6	24.7	16.8	10.8	7.6	4.4	2.5	2.8	4.9
2018 F	0.6	3.3	7.8	10.1	10.3	9.0	6.1	6.3	3.7	2.3	3.1	2.5
2019 M	1.2	8.6	19.3	27.1	29.0	19.9	13.1	7.1	4.7	2.6	2.4	4.3
2019 F	0.6	3.5	7.8	10.4	10.6	10.4	8.3	6.8	3.9	3.7	2.7	3.3

Table S5. Mortality rate for external causes (deaths per 100,000) in the general population, by age group, year, sex and setting.

British Columbia

Year - Sex	Age group, years											
	10-19	20-29	30-39	40-44	45-49	50-54	55-59	60-64	65-69	70-74	75-79	80+
2014 M	21.3	40.9	37.6	46.0	51.7	51.7	63.9	58.7	40.2	72.4	100.5	278.2
2014 F	6.6	11.4	9.8	11.6	8.1	15.8	8.9	19.2	19.8	38.1	61.2	224.0
2015 M	16.8	45.1	37.2	39.7	45.9	53.0	67.6	56.6	48.4	66.3	108.2	303.0
2015 F	6.1	10.5	7.8	9.3	11.5	9.5	20.1	10.4	15.7	24.3	52.6	243.2
2016 M	17.5	42.0	37.2	39.0	49.3	42.6	47.3	59.1	60.5	64.2	101.6	268.2
2016 F	7.3	15.1	10.0	11.3	12.0	12.2	13.8	14.1	18.3	32.8	52.0	195.5
2017 M	16.3	35.9	33.8	35.1	36.9	32.0	53.4	41.4	44.3	66.7	92.8	241.8
2017 F	5.6	9.7	9.8	12.0	12.6	13.5	7.9	14.3	14.0	25.7	39.7	188.3
2018 M	12.8	33.3	33.0	31.5	41.6	44.7	45.8	46.9	49.2	52.0	77.0	257.6
2018 F	7.1	11.6	12.1	6.9	7.5	16.6	13.0	17.3	14.3	18.2	39.0	203.9
2019 M	11.7	32.1	28.0	32.3	31.8	41.6	37.9	40.8	43.5	58.7	96.8	257.1
2019 F	8.2	10.5	5.9	5.6	11.8	9.6	8.8	11.5	16.4	17.2	41.5	210.4

Scotland

Year - Sex	Age group, years											
	10-19	20-29	30-39	40-44	45-49	50-54	55-59	60-64	65-69	70-74	75-79	80+
2014 M	11.8	28.9	32.2	47.4	43.9	38.6	42.8	44.8	46.7	55.6	86.6	281.8
2014 F	3.5	10.4	9.8	11.9	15.9	13.2	13.2	17.9	23.4	25.1	58.4	272.4
2015 M	12.9	30.2	38.2	47.0	43.3	42.6	44.1	41.5	52.2	67.6	90.7	284.3
2015 F	2.1	7.6	8.8	12.3	16.7	13.9	16.7	20.3	17.3	24.1	54.4	292.5
2016 M	10.2	36.7	38.4	58.2	48.0	46.1	44.3	43.5	45.6	71.5	116.3	340.9
2016 F	5.7	11.1	11.3	10.4	15.3	11.5	14.2	14.5	22.6	29.9	65.2	292.5
2017 M	11.3	33.6	42.9	47.7	49.1	48.6	49.3	44.4	40.7	60.5	116.0	322.6
2017 F	2.9	6.8	8.8	10.3	14.5	15.7	12.9	17.7	20.3	28.4	79.3	335.2
2018 M	14.4	35.1	41.6	41.1	54.8	48.5	44.1	41.7	50.3	49.9	97.6	326.3
2018 F	3.6	8.8	9.4	11.7	13.4	13.9	12.6	17.9	25.1	32.3	54.7	303.3
2019 M	11.9	38.4	43.4	56.8	53.2	43.3	50.9	43.8	51.2	65.7	111.5	335.1
2019 F	5.3	10.8	9.5	8.0	13.4	13.0	24.2	14.1	17.4	31.4	62.9	343.9

England

Year - Sex	Age group, years											
	10-19	20-29	30-39	40-44	45-49	50-54	55-59	60-64	65-69	70-74	75-79	80+
2014 M	8.1	24.0	23.8	33.0	35.1	32.1	32.1	34.6	30.7	45.1	73.6	233.2
2014 F	3.2	6.3	6.9	8.0	9.7	11.1	11.8	12.6	15.8	25.2	46.9	197.5
2015 M	8.3	25.3	26.0	34.0	34.1	34.3	33.8	35.4	35.3	46.1	83.8	267.7
2015 F	3.4	6.1	6.5	7.8	9.8	11.7	11.4	14.0	16.9	26.4	52.1	219.9
2016 M	8.7	24.2	26.9	32.9	34.6	33.9	34.6	34.7	38.5	47.8	83.0	263.0
2016 F	3.2	5.9	7.8	8.7	10.7	11.9	11.5	13.9	16.6	25.9	51.8	213.1
2017 M	9.5	25.2	29.8	32.1	36.2	35.7	33.9	36.2	38.6	50.4	85.4	278.1
2017 F	4.0	6.5	6.5	8.2	9.4	10.0	11.4	14.9	17.0	28.7	54.4	228.3
2018 M	8.7	26.6	28.2	31.8	37.6	37.3	34.5	37.9	38.3	50.7	89.3	282.1
2018 F	3.8	7.3	7.3	9.0	10.8	10.9	13.8	15.5	19.8	29.0	55.0	225.9
2019 M	7.8	24.4	29.7	32.1	37.6	35.9	32.4	36.5	38.2	49.7	82.6	272.9
2019 F	3.1	6.6	7.0	8.6	10.8	9.9	12.2	13.4	18.1	25.9	50.5	224.4

Table S6. Mortality rate for extrahepatic cancer (deaths per 100,000) in the general population, by age group, year, sex and setting.

British Columbia

Year - Sex	Age group, years											
	10-19	20-29	30-39	40-44	45-49	50-54	55-59	60-64	65-69	70-74	75-79	80+
2014 M	2.7	6.0	13.5	24.9	49.3	99.5	193.3	322.6	511.8	853.1	1168.5	2206.4
2014 F	2.5	5.4	14.3	44.7	61.5	110.3	151.5	265.6	447.4	605.2	878.5	1334.0
2015 M	3.8	4.8	12.6	18.2	45.9	90.4	179.6	298.9	475.6	791.3	1144.3	2104.8
2015 F	1.6	4.1	13.4	32.2	45.0	96.6	182.7	288.6	390.6	666.8	840.5	1374.7
2016 M	4.2	3.0	7.6	25.8	41.4	82.5	160.9	298.8	531.2	713.4	1211.9	2097.9
2016 F	1.6	3.2	12.7	27.6	52.9	109.9	170.3	279.5	391.1	574.8	851.2	1389.7
2017 M	2.3	9.6	10.1	21.2	42.4	88.6	169.1	315.8	512.2	767.6	1100.4	2130.0
2017 F	2.4	3.1	13.7	32.7	62.8	104.7	160.5	247.0	373.6	595.4	881.2	1363.2
2018 M	1.9	4.3	9.5	18.4	42.3	84.7	176.1	297.6	462.5	749.7	1128.2	2058.6
2018 F	1.2	4.0	9.8	30.8	46.7	81.3	167.8	248.6	349.6	527.5	784.4	1368.9
2019 M	4.9	5.9	11.4	20.0	45.5	92.2	168.5	278.3	480.3	704.7	1091.1	2070.0
2019 F	2.4	3.0	12.1	33.0	61.7	83.3	167.6	244.8	350.7	541.6	799.8	1345.6

Scotland

Year - Sex	Age group, years											
	10-19	20-29	30-39	40-44	45-49	50-54	55-59	60-64	65-69	70-74	75-79	80+
2014 M	2.7	2.8	12.4	37.7	69.4	127.6	253.1	471.1	730.8	1187.5	1697.1	2810.7
2014 F	3.5	4.1	16.4	53.9	78.0	144.0	242.3	361.2	540.1	861.1	1183.3	1829.3
2015 M	3.7	5.8	14.4	36.4	71.5	137.4	253.6	461.6	767.1	1206.4	1683.1	2774.4
2015 F	1.8	6.8	20.3	36.7	89.1	144.2	220.7	333.0	550.6	868.9	1139.6	1825.9
2016 M	2.1	6.0	13.2	41.8	86.1	127.6	232.9	444.4	740.2	1106.7	1594.9	2826.8
2016 F	2.5	5.1	21.4	56.2	75.9	134.3	227.3	330.0	517.5	818.7	1155.0	1817.5
2017 M	3.8	2.7	14.1	37.0	71.7	117.0	215.6	408.9	699.6	1114.8	1672.0	2942.8
2017 F	3.2	3.8	19.8	38.7	73.1	151.2	219.8	329.4	547.9	787.3	1248.9	1813.2
2018 M	4.1	3.8	14.3	32.8	67.6	119.0	243.0	427.0	687.5	1028.4	1699.2	2668.7
2018 F	2.5	3.6	21.1	45.1	69.2	144.6	227.6	350.2	540.5	825.3	1162.5	1771.9
2019 M	2.0	3.8	15.2	42.1	60.6	121.2	224.9	424.3	668.0	1019.4	1597.5	2764.0
2019 F	2.5	4.1	19.4	49.8	78.2	130.3	216.5	362.7	550.4	774.7	1188.2	1814.1

England

Year - Sex	Age group, years											
	10-19	20-29	30-39	40-44	45-49	50-54	55-59	60-64	65-69	70-74	75-79	80+
2014 M	3.0	5.3	12.2	30.0	57.2	111.5	219.0	391.4	617.0	967.2	1424.2	2639.7
2014 F	2.1	5.6	18.1	43.2	72.0	120.7	198.1	314.8	456.2	684.7	937.5	1602.9
2015 M	3.2	5.2	13.0	28.5	56.6	109.2	212.8	387.0	614.7	955.9	1415.7	2602.5
2015 F	2.2	5.1	17.8	39.4	72.2	120.4	195.5	307.4	458.0	666.5	955.6	1587.6
2016 M	2.8	5.1	13.0	29.2	57.3	111.6	206.4	377.2	613.1	931.9	1430.6	2585.6
2016 F	2.4	5.1	19.3	41.0	71.2	122.3	201.5	301.5	464.2	672.2	982.1	1579.3
2017 M	2.8	4.6	12.6	31.7	58.1	110.3	200.6	366.4	598.6	910.6	1399.5	2554.4
2017 F	2.4	4.0	18.0	39.5	69.0	118.3	183.1	293.9	449.5	652.6	945.9	1571.7
2018 M	3.2	4.9	13.4	27.9	56.4	109.6	198.4	346.2	587.4	870.0	1356.8	2487.6
2018 F	2.0	5.4	18.8	37.0	72.6	111.9	193.3	288.8	432.1	635.9	924.6	1531.9
2019 M	2.4	5.1	14.1	27.8	53.2	106.5	186.1	337.5	574.1	858.4	1325.6	2474.3
2019 F	1.8	4.2	17.6	37.2	69.4	116.9	178.0	281.7	413.9	629.1	898.5	1552.2

Table S7. Mortality rate for circulatory disease (deaths per 100,000) in the general population, by age group, year, sex and setting.

British Columbia

Year - Sex	Age group, years											
	10-19	20-29	30-39	40-44	45-49	50-54	55-59	60-64	65-69	70-74	75-79	80+
2014 M	0.4	1.5	6.4	16.0	47.5	59.8	106.2	171.5	250.0	476.7	871.6	2989.6
2014 F	1.2	1.0	4.4	10.4	16.3	24.8	33.0	67.2	121.2	202.1	522.6	3008.1
2015 M	0.0	3.9	6.9	14.3	39.9	64.4	115.4	174.2	265.0	426.6	748.1	3182.1
2015 F	0.8	1.9	4.0	3.7	11.0	17.3	34.8	66.9	123.5	229.7	508.2	3045.7
2016 M	0.0	5.3	8.2	23.1	30.6	67.2	121.9	183.7	306.9	439.3	795.2	3127.7
2016 F	0.8	1.9	3.6	5.6	17.1	21.7	40.8	58.3	113.9	228.0	504.9	3040.8
2017 M	0.4	2.6	8.6	19.9	39.9	72.9	117.9	194.1	296.9	449.1	805.3	3191.2
2017 F	0.4	2.5	3.3	6.9	16.0	25.9	47.4	62.9	107.9	233.0	509.8	3035.7
2018 M	1.1	2.9	12.3	22.3	30.6	58.6	120.5	199.0	276.0	482.4	773.8	2952.2
2018 F	0.0	0.9	4.9	11.3	11.5	24.9	29.7	64.7	110.0	223.6	468.9	2900.6
2019 M	0.0	3.1	8.6	19.4	34.9	61.3	111.6	169.2	262.2	416.1	711.5	2882.2
2019 F	0.4	3.3	3.4	10.6	16.5	22.5	27.4	63.0	112.5	204.3	473.7	2853.0

Scotland

Year - Sex	Age group, years											
	10-19	20-29	30-39	40-44	45-49	50-54	55-59	60-64	65-69	70-74	75-79	80+
2014 M	0.0	4.7	15.5	54.2	79.1	129.6	185.5	295.2	482.3	834.4	1429.9	3679.2
2014 F	0.4	1.6	6.9	22.1	31.3	48.5	88.1	120.6	240.6	418.0	911.4	3300.7
2015 M	0.7	2.8	21.7	52.9	79.9	122.7	194.3	318.3	493.1	894.2	1397.8	3823.9
2015 F	0.7	1.9	10.6	19.5	32.8	48.1	66.2	121.7	235.3	471.9	927.9	3430.9
2016 M	0.7	4.6	14.4	49.1	80.8	138.3	221.3	299.9	478.9	809.0	1367.0	3576.0
2016 F	0.4	1.9	11.0	23.7	28.1	44.9	76.8	138.7	231.0	444.4	871.3	3165.0
2017 M	1.0	4.3	19.4	43.9	89.0	125.1	209.1	322.6	505.5	756.8	1418.5	3439.4
2017 F	1.4	2.2	8.8	26.6	27.1	55.3	76.2	143.7	227.7	423.5	848.5	3139.4
2018 M	1.0	3.8	14.1	47.6	74.2	123.0	194.6	305.1	460.0	738.6	1299.4	3449.5
2018 F	0.7	2.2	5.0	18.5	26.8	56.0	67.5	124.8	220.7	387.1	837.3	2919.3
2019 M	0.3	2.7	15.8	45.9	76.1	137.7	187.1	288.9	456.2	700.5	1293.7	3303.4
2019 F	1.1	3.9	7.9	17.8	40.7	44.9	74.6	137.8	231.6	384.9	786.6	2896.7

England

Year - Sex	Age group, years											
	10-19	20-29	30-39	40-44	45-49	50-54	55-59	60-64	65-69	70-74	75-79	80+
2014 M	1.1	3.2	12.6	35.1	59.8	97.2	159.8	250.1	370.2	652.4	1128.4	3249.1
2014 F	0.7	2.0	5.8	15.4	23.0	35.8	58.7	97.3	162.4	328.8	684.7	2877.3
2015 M	1.3	4.0	11.2	32.4	60.7	101.1	158.7	257.5	377.3	631.1	1129.5	3234.8
2015 F	0.5	2.0	5.8	14.1	23.9	35.3	58.6	103.6	169.9	332.3	670.4	2880.4
2016 M	1.5	3.8	12.9	34.3	62.6	96.9	156.7	253.4	381.0	627.5	1091.9	3082.3
2016 F	0.7	2.0	6.4	13.7	24.3	38.2	60.4	104.7	166.6	317.2	643.8	2706.6
2017 M	1.2	4.1	12.7	34.2	60.0	101.6	160.9	247.7	368.4	593.8	1051.7	3032.9
2017 F	0.8	2.0	5.6	14.6	22.5	38.4	54.8	101.8	166.9	298.1	651.4	2633.6
2018 M	1.0	3.5	12.9	34.6	60.5	101.4	159.0	244.6	374.5	581.0	993.3	2893.9
2018 F	1.0	2.5	5.4	14.6	25.0	36.0	59.0	98.3	166.8	286.8	593.1	2486.2
2019 M	1.1	3.1	12.2	32.9	61.6	102.6	158.2	237.1	368.1	572.6	985.8	2808.0
2019 F	0.8	2.0	6.0	13.5	22.3	34.4	60.2	101.2	164.5	279.8	569.0	2398.2

Table S8. Mortality rate for other causes of death (deaths per 100,000) in the general population, by age group, year, sex and setting

British Columbia

Year - Sex	Age group, years											
	10-19	20-29	30-39	40-44	45-49	50-54	55-59	60-64	65-69	70-74	75-79	80+
2014 M	3.4	12.0	17.4	28.7	42.1	72.3	130.1	207.1	321.8	650.4	1058.5	3569.9
2014 F	2.1	5.7	11.4	17.8	21.5	42.2	64.8	105.0	193.6	390.4	700.6	3565.6
2015 M	5.7	15.2	23.0	30.6	45.3	71.4	117.7	180.6	331.9	593.5	1141.2	3871.7
2015 F	5.3	5.1	9.0	18.0	25.4	41.5	65.8	128.3	211.3	387.6	821.1	3735.1
2016 M	5.7	17.4	23.2	30.4	52.3	69.4	120.2	219.1	368.1	623.7	1144.2	3884.9
2016 F	4.1	7.9	12.7	15.7	27.3	45.4	87.0	121.8	216.2	402.8	821.9	3640.3
2017 M	6.8	19.6	24.6	38.4	59.3	81.9	136.6	204.4	356.0	625.7	1204.4	4078.4
2017 F	6.8	11.9	14.5	15.7	30.3	47.5	71.6	112.6	237.1	417.3	764.6	3922.6
2018 M	14.7	44.1	59.3	84.7	84.5	120.1	165.2	236.4	356.4	583.9	1108.3	3894.6
2018 F	6.3	16.5	25.4	22.6	37.5	61.4	71.4	142.2	223.3	393.4	800.2	3813.0
2019 M	8.6	52.5	77.8	78.7	109.0	143.4	174.4	239.2	375.7	622.5	1107.4	3897.2
2019 F	5.5	19.6	33.9	35.5	51.7	62.5	84.8	130.3	204.7	376.5	852.9	3784.0

Scotland

Year - Sex	Age group, years											
	10-19	20-29	30-39	40-44	45-49	50-54	55-59	60-64	65-69	70-74	75-79	80+
2014 M	6.1	9.7	23.2	49.1	74.0	94.1	160.7	255.0	400.3	779.8	1482.4	4698.0
2014 F	5.7	8.8	12.5	29.6	47.6	67.6	116.2	214.0	330.5	594.6	1259.0	4883.8
2015 M	5.1	12.4	26.0	52.3	74.1	100.9	173.4	252.2	441.6	879.3	1611.8	5179.1
2015 F	5.7	8.4	18.2	35.1	46.0	76.4	125.4	199.7	359.8	673.6	1280.7	5466.3
2016 M	11.3	13.6	25.5	62.5	81.3	121.6	166.0	263.4	472.4	810.8	1476.1	4873.3
2016 F	4.6	6.8	15.6	28.4	54.2	78.4	128.4	247.7	351.7	674.0	1303.5	5160.5
2017 M	5.8	9.7	26.7	49.5	80.3	118.1	154.4	260.1	418.7	784.6	1627.8	5164.6
2017 F	6.8	8.7	19.2	32.6	44.1	64.4	131.8	228.9	352.6	643.7	1362.6	5468.7
2018 M	5.1	14.7	22.7	46.9	74.2	110.3	164.9	261.0	461.3	726.9	1588.7	5070.2
2018 F	8.6	10.7	17.8	33.3	57.3	83.3	126.4	214.4	363.6	651.4	1316.2	5397.7
2019 M	7.5	11.7	24.2	49.8	81.2	101.1	137.9	265.5	434.7	764.0	1457.6	4825.2
2019 F	4.3	6.9	17.5	41.2	44.5	76.3	118.6	205.2	354.2	615.6	1215.0	5181.3

England

Year - Sex	Age group, years											
	10-19	20-29	30-39	40-44	45-49	50-54	55-59	60-64	65-69	70-74	75-79	80+
2014 M	6.6	10.5	17.3	29.3	45.1	63.8	101.1	183.9	311.8	611.6	1214.9	4590.9
2014 F	4.9	6.7	12.3	21.1	29.2	46.9	76.9	144.5	238.6	481.7	984.1	4914.9
2015 M	6.2	11.2	17.4	32.8	47.8	72.2	112.2	191.6	320.3	626.8	1294.5	5030.7
2015 F	5.8	7.6	11.4	20.0	31.0	51.0	80.0	141.8	251.4	491.2	1035.7	5522.3
2016 M	5.4	11.2	17.2	34.6	49.5	73.0	117.5	200.6	341.3	639.7	1337.0	4954.9
2016 F	5.1	8.1	13.9	24.1	32.7	52.2	88.8	156.3	262.8	514.0	1044.9	5350.0
2017 M	6.7	11.4	18.7	31.8	54.8	73.0	111.9	197.3	349.1	635.2	1341.2	5020.2
2017 F	4.4	7.1	12.1	21.0	34.3	51.0	85.6	148.8	261.8	482.0	1057.9	5493.4
2018 M	5.2	11.5	18.2	33.5	50.1	79.0	118.2	199.6	357.1	635.1	1328.5	4992.4
2018 F	4.4	7.0	12.5	21.9	35.3	54.7	87.1	149.2	261.8	493.6	1062.4	5487.5
2019 M	6.9	12.3	19.4	30.9	51.3	75.8	121.3	191.1	356.0	609.9	1265.7	4863.9
2019 F	4.2	7.3	12.9	20.7	32.8	52.5	82.4	148.6	256.3	479.7	1009.7	5147.2

Table S9. Methods used to identify compensated cirrhosis and end-stage liver disease (ESLD) at SVR achievement, by setting

	Setting		
	British Columbia	Scotland	England
Compensated cirrhosis	Pre-SVR emergency department visits, inpatient and outpatient hospital admissions data using the following ICD10 codes:<br>K703 (alcohol cirrhosis of liver); K717 (Toxic liver disease with fibrosis and cirrhosis of liver); K746 (other and unspecified cirrhosis of liver); and K766 (portal hypertension)<br>Note that if an ESLD code is also present, the individual will be assigned to the ESLD group.	Clinical diagnosis of cirrhosis without record of ESLD (derived from medical notes)	Clinical diagnosis of cirrhosis without record of ESLD (derived from medical notes)
End stage liver disease (ESLD)	Pre-SVR emergency department visits, cancer registrations, inpatient and outpatient hospital admissions data using the following ICD10 codes:<br>K721 (chronic hepatic failure); K729 (hepatic failure, unspecified); K704 (alcoholic hepatic failure); K767 (hepatorenal syndrome); I850 and I983 (oesophageal varices with bleeding); R18 (ascites); C22 (primary liver cancer)<br>Note that, because ascites can have non-hepatic causes, R18 was only included if accompanied by a corroborating liver code (K70-K77).	Diagnosis of: a) bleeding varices; b) ascites; c) hepatic encephalopathy; or d) hepatocellular carcinoma, derived from informationrecorded in medical notes.	Diagnosis of: a) bleeding varices; b) ascites; c) hepatic encephalopathy; or d) hepatocellular carcinoma, derived from informationrecorded in medical notes.

N.B. pre-cirrhosis defined by the absence of compensated cirrhosis and ESLD

HCC=hepatocellular carcinoima; ICD= International Classification of Disease

Table S10. ICD codes used to define cause-specifc categories of death

Cause of death	ICD definition
Primary liver cancer	C22: Malignant neoplasm of liver and intrahepatic bile ducts
Liver failure	K70: Alcohol liver disease<br>K71-K77: Non-alcoholic liver disease<br>B15-B19: Viral hepatitis<br>B942: Sequelae of viral hepatitis<br>I850, I982 and I983: Oesophageal varices
Drug-related	F11-F16 and F19: Mental/behavioural disorders due to psychoactive substance use<br>X40-X44: Unintentional drug poisoning<br>X60-X64: Intentional drug poisoning<br>Y10-Y14: Drug-related poisoning of undetermined intent
External causes	V00-X39 and X45-X59: Accidents, excluding unintentional drug poisoning<br>X65-X84: Suicide, excluding intentional drug-related overdoses<br>X85-Y09: Homicide<br>Y15-Y99: Other external cause
Extrahepatic cancer	C00-D49: Neoplasms (excluding primary liver cancer, C22)
Diseases of circulatory system	I00-I99: Diseases of the circulatory system (excluding oesophageal varices; I850; I982, I983).
Other (non of the above)	All ICD-10 codes not listed above

ICD-10: International Classification of Diseases

Table S11: ICD-10 codes used to identify hospital admissions relating to alcohol and substance use.

	ICD-10 codes
Alcohol use*	E24.4: alcohol-induced Pseudo Cushing's syndrome
	E51.2: Wernicke's encephalopathy
	F10: Mental and behavioural disorders due to use of alcohol
	G31.2: Degeneration of nervous system due to alcohol
	G62.1: Alcoholic polyneuropathy
	G72.1: Alcoholic myopathy
	I42.6: Alcoholic cardiomyopathy
	K29.2: Alcoholic gastritis
	K70: Alcoholic liver disease
	K86.0: Alcohol-induced chronic pancreatitis
	PO4.3 AND O35.4: Fetus and newborn affected by maternal use of alcohol
	Q86.0: Fetal alcohol syndrome
	R78.0: Excessive blood level of alcohol
	T51.0 and T51.9: Toxic effect of alcohol
	X45, X65 and Y15: Alcohol poisoning
	Y90: High blood alcohol level
	Y91: Evidence of alcohol involvement determine by level of intoxication
Substance use	F11-F19: Mental and behaviour disorders due to psychoactive substance use
	T40: poisoning by narcotics and psychodysleptics

* comprised of conditions wholly attributed to alcohol use; identified in a previous review by NHS National Services Scotland. Available at: https://www.scotpho.org.uk/publications/reports-and-papers/alcohol-attributable-mortality-and-morbidity-alcohol-population-attributable-fractions-for-scotland/

Table S12: Standard populations underpinning standardised mortality rates.
Values represent the proportion of follow-up time acrrued in each age-sex group in British Columbia, by disease stage

Age group	Sex	Liver disease severity group			All patients
		Pre-cirrhosis	Cirrhosis	ESLD	
<20	Male	0.000	0.000	0.000	0.000
	Female	0.000	0.000	0.000	0.000
20-29	Male	0.006	0.000	0.000	0.005
	Female	0.009	0.000	0.001	0.007
30-39	Male	0.031	0.015	0.012	0.028
	Female	0.026	0.003	0.003	0.022
40-44	Male	0.028	0.011	0.012	0.025
	Female	0.020	0.005	0.005	0.017
45-49	Male	0.051	0.024	0.036	0.047
	Female	0.028	0.013	0.022	0.026
50-54	Male	0.092	0.091	0.055	0.089
	Female	0.049	0.037	0.049	0.048
55-59	Male	0.142	0.143	0.148	0.143
	Female	0.074	0.081	0.076	0.075
60-64	Male	0.179	0.216	0.229	0.187
	Female	0.078	0.097	0.126	0.083
65-69	Male	0.090	0.130	0.112	0.096
	Female	0.040	0.054	0.052	0.043
70-74	Male	0.025	0.034	0.030	0.026
	Female	0.010	0.021	0.012	0.011
75-79	Male	0.007	0.006	0.004	0.007
	Female	0.008	0.012	0.012	0.009
80+	Male	0.004	0.007	0.003	0.004
	Female	0.004	0.001	0.004	0.003

Table S13: Specification of standardied mortality ratio (SMR) and mortality rate models

Attribute	**Modelling SMR**	**Modelling mortality rate**
Regression model:	Poisson	Poisson
Dependent variable:	death (any cause)	death (any cause)
Independent variables:	Acquired age; sex; year; alcohol-related hospital admission; substance use related hospital admission; comorbidity score	Acquired age; sex; year; alcohol-related hospital admission; substance use related hospital admission; comorbidity score
Data structure:	Lexis expansion (split by age group, sex, calendar year)	Lexis expansion (split by age group, sex, calendar year)
Standard errors:	Robust (clustered by individual ID)	Robust (clustered by individual ID)
Offset:	Number of expected deaths (i.e. age/sex/year specific mortality rate for general population * person years of follow-up)	Person years of follow-up
Interpretation of exponentiated beta coefficient:	SMR ratio	Mortality rate ratio

Table S14: Number of deaths in each setting, by cause of death

Cause of death	BC	England	Scotland	Total
All causes	821	309	442	1572
Liver cancer	116	83	51	250
Liver failure	130	112	44	286
Drug-related	183	10	190	383
External causes	33	6	28	67
Extrahepatic cancer	123	24	34	181
Circulatory diseases	104	36	36	176
Other	132	38	59	229

Table S15. Factors associated with all-cause mortality rate in **univariable** analysis, by liver disease severity and setting

Characteristic		British Columbia; N= 11,942						Scotland; N= 7691						England; N=2157			
		Pre-cirrhosis		Cirrhosis		ESLD		Pre-cirrhosis		Cirrhosis		ESLD.		Cirrhosis		ESLD	
		Mortality rate ratio (95%CI)	P	Mortality rate ratio (95%CI)	P	Mortality rate ratio (95%CI)	P	Mortality rate ratio (95%CI)	P	Mortality rate ratio (95%CI)	P	Mortality rate ratio (95%CI)	P	Mortality rate ratio (95%CI)	P	Mortality rate ratio (95%CI)	P
Year cure achieved		1.11 (1.03 to 1.20)	0.004	0.91 (0.77 to 1.07)	0.24	0.82 (0.73 to 0.91)	<0.001	1.05 (0.97 to 1.14)	0.24	1.06 (0.95 to 1.19)	0.32	0.98 (0.79 to 1.23)	0.89	1.09 (0.93 to 1.27)	0.26	0.94 (0.85 to 1.03)	0.19
Age, per 10 year increase		1.22 (1.11 to 1.34)	<0.001	1.29 (0.98 to 1.69)	0.07	1.28 (1.03 to 1.58)	0.024	1.19 (1.05 to 1.34)	0.006	1.13 (0.97 to 1.31)	0.12	1.17 (0.91 to 1.51)	0.21	1.37 (1.13 to 1.66)	0.001	1.08 (0.90 to 1.30)	0.42
Gender	Male	REF (1.00)		REF (1.00)		REF (1.00)		REF (1.00)		REF (1.00)		REF (1.00)		REF (1.00)		REF (1.00)	
	Female	0.69 (0.56 to 0.84)	<0.001	0.57 (0.35 to 0.92)	0.02	0.76 (0.57 to 1.02)	0.07	0.69 (0.50 to 0.95)	0.02	0.89 (0.62 to 1.28)	0.54	0.79 (0.43 to 1.42)	0.43	0.87 (0.57 to 1.33)	0.53	1.07 (0.77 to 1.47)	0.70
Hospital admission	No	REF (1.00)		REF (1.00)		REF (1.00)		REF (1.00)		REF (1.00)		REF (1.00)		REF (1.00)		REF (1.00)	
for alcohol use	Yes (not recent)	1.71 (1.28 to 2.30)	<0.001	1.43 (0.73 to 2.77)	0.30	1.22 (0.81 to 1.85)	0.34	1.44 (1.01 to 2.07)	0.04	1.37 (0.87 to 2.15)	0.18	0.73 (0.28 to 1.94)	0.53	1.46 (0.81 to 2.62)	0.21	0.73 (0.42 to 1.28)	0.28
	Yes (recent)	4.00 (3.07 to 5.21)	<0.001	2.45 (1.47 to 4.06)	0.001	1.38 (1.00 to 1.90)	0.05	2.73 (1.88 to 3.96)	<0.001	2.06 (1.44 to 2.95)	<0.001	1.66 (0.95 to 2.89)	0.07	1.91 (1.21 to 2.99)	0.005	1.79 (1.32 to 2.42)	<0.001
Hospital admission	No	REF (1.00)		REF (1.00)		REF (1.00)		REF (1.00)		REF (1.00)		REF (1.00)		REF (1.00)		REF (1.00)	
for substance use	Yes (not recent)	2.05 (1.61 to 2.60)	<0.001	1.16 (0.62 to 2.17)	0.64	1.14 (0.73 to 1.77)	0.57	1.85 (1.34 to 2.56)	<0.001	1.50 (1.02 to 2.20)	0.04	1.06 (0.51 to 2.24)	0.87	1.80 (1.04 to 3.10)	0.04	1.08 (0.61 to 1.92)	0.79
	Yes (recent)	3.93 (3.17 to 4.87)	<0.001	1.83 (0.92 to 3.65)	0.09	1.65 (1.12 to 2.43)	0.01	3.08 (2.26 to 4.19)	<0.001	2.62 (1.83 to 3.74)	<0.001	1.96 (1.09 to 3.54)	0.03	1.96 (1.32 to 2.91)	0.001	1.85 (1.37 to 2.49)	<0.001
Charlson comorbidity index		0.80 (0.78 to 0.82)*	<0.001	0.35 (0.21 to 0.58)**	<0.001	1.24 (1.16 to 1.34)	<0.001	1.34 (1.19 to 1.50)	<0.001	0.25 (0.17 to 0.38)**	<0.001	1.18 (0.97 to 1.43)	0.09	0.87 (0.81 to 0.92)*	<0.001	1.04 (0.94 to 1.16)	0.41

* First degree fractional polynomial : power = -1
** First degree fractional polynomial : power = -2

Table S16. Factors associated with standardised mortality ratio in **univariable** analysis, by liver disease severity and setting

Characteristic		British Columbia; N= 11,942						Scotland; N= 7691						England; N=2157			
		Pre-cirrhosis		Cirrhosis		ESLD		Pre-cirrhosis		Cirrhosis		ESLD.		Cirrhosis		ESLD	
		SMR ratio (95%CI)	P	SMR ratio (95%CI)	P	SMR ratio (95%CI)	P	SMR ratio (95%CI)	P	SMR ratio (95%CI)	P	SMR ratio (95%CI)	P	SMR ratio (95%CI)	P	SMR ratio (95%CI)	P
Year cure achieved		1.07 (0.99 to 1.16)	0.09	0.86 (0.73 to 1.02)	0.09	0.77 (0.69 to 0.87)	<0.001	1.04 (0.94 to 1.15)	0.42	1.04 (0.91 to 1.19)	0.53	1.11 (0.88 to 1.41)	0.38	1.01 (0.86 to 1.18)	0.94	0.83 (0.75 to 0.82)	0.001
Age, per 10 year increase		0.54 (0.50 to 0.59)	<0.001	0.54 (0.43 to 0.69)	<0.001	0.54 (0.45 to 0.66)	<0.001	0.57 (0.51 to 0.64)	<0.001	0.99 (0.99 to 0.99)**	<0.001	0.52 (0.41 to 0.65)	<0.001	0.56 (0.47 to 0.67)	<0.001	0.45 (0.38 to 0.53)	<0.001
Gender	Male	REF (1.00)		REF (1.00)		REF (1.00)		REF (1.00)		REF (1.00)		REF (1.00)		REF (1.00)		REF (1.00)	
	Female	1.10 (0.89 to 1.36)	0.38	0.89 (0.54 to 1.46)	0.64	1.14 (0.81 to 1.60)	0.44	0.92 (0.65 to 1.30)	0.62	0.97 (0.64 to 1.47)	0.88	0.94 (0.47 to 1.88)	0.86	0.96 (0.61 to 1.51)	0.85	1.15 (0.79 to 1.66)	0.48
Hospital admission	No	REF (1.00)		REF (1.00)		REF (1.00)		REF (1.00)		REF (1.00)		REF (1.00)		REF (1.00)		REF (1.00)	
for alcohol use	Yes (not recent)	2.49 (1.84 to 3.37)	<0.001	1.93 (0.98 to 3.81)	0.06	1.44 (0.93 to 2.24)	0.11	1.73 (1.20 to 2.50)	0.003	1.75 (1.08 to 2.83)	0.02	0.98 (0.35 to 2.77)	0.97	1.66 (0.91 to 3.06)	0.10	1.01 (0.56 to 1.81)	0.98
	Yes (recent)	4.92 (3.59 to 6.74)	<0.001	3.55 (2.10 to 5.98)	<0.001	1.95 (1.39 to 2.76)	<0.001	3.27 (2.22 to 4.83)	<0.001	2.95 (2.00 to 4.36)	<0.001	2.54 (1.38 to 4.70)	0.003	2.61 (1.64 to 4.17)	<0.001	3.07 (2.21 to 4.27)	<0.001
Hospital admission	No	REF (1.00)		REF (1.00)		REF (1.00)		REF (1.00)		REF (1.00)		REF (1.00)		REF (1.00)		REF (1.00)	
for substance use	Yes (not recent)	3.56 (2.79 to 4.54)	<0.001	1.75 (0.93 to 3.32)	0.08	1.72 (1.06 to 2.78)	0.03	2.65 (1.91 to 3.69)	<0.001	2.46 (1.64 to 3.68)	<0.001	2.60 (1.19 to 5.65)	0.02	2.47 (1.39 to 4.37)	0.002	1.71 (0.93 to 3.12)	0.08
	Yes (recent)	7.43 (5.94 to 9.30)	<0.001	3.38 (1.68 to 6.80)	0.001	2.77 (1.83 to 4.19)	<0.001	4.76 (3.46 to 6.53)	<0.001	4.49 (3.06 to 6.59)	<0.001	3.89 (2.04 to 7.42)	<0.001	2.86 (1.91 to 4.28)	<0.001	3.14 (2.27 to 4.34)	<0.001
Charlson comorbidity index		0.38 (0.33 to 0.43)*	<0.001	1.27 (1.11 to 1.45)	<0.001	1.21 (1.12 to 1.31)	<0.001	1.24 (1.10 to 1.39)	<0.001	1.29 (1.14 to 1.45)	<0.001	1.01 (0.79 to 1.30)	0.94	1.15 (1.01 to 1.30)	0.03	0.90 (0.79 to 1.03)	0.14

* First degree fractional polynomial : power = -0.5
** First degree fractional polynomial : power =3

Interpretation:
The Standardised mortality ratio (SMR) is the ratio of observed to expected death, where expected deaths indicate the number of deaths that would have occurred if cured patients exhibited the same age/sex/year specific mortality rates as the corresponding general population.
The *SMR ratio* indicates the ratio of SMR in the group of interest relative to the reference group. For example, if the SMR ratio for individuals with a recent substance use hospital admission is 3.0, this means the SMR is three time higher in this group versus individuals with no previous admission.

Table S17. Factors associated with standardised mortality ratio in multivariable analysis, by liver disease severity and setting

Characteristic		**British Columbia; N= 11,942**						**Scotland; N= 7691**						**England; N=2157**			
		Pre-cirrhosis		Cirrhosis		ESLD		Pre-cirrhosis		Cirrhosis		ESLD.		Cirrhosis		ESLD	
		SMR ratio (95%CI)	P	SMR ratio (95%CI)	P	SMR ratio (95%CI)	P	SMR ratio (95%CI)	P	SMR ratio (95%CI)	P	SMR ratio (95%CI)	P	SMR ratio (95%CI)	P	SMR ratio (95%CI)	P
Year cure achieved		1.06 (0.98 to 1.15)	0.15	0.86 (0.73 to 1.02)	0.08	0.79 (0.70 to 0.89)	<0.001	0.98 (0.90 to 1.07)	0.66	0.96 (0.85 to 1.09)	0.55	0.99 (0.80 to 1.24)	0.95	1.03 (0.88 to 1.20)	0.72	0.93 (0.84 to 1.03)	0.18
Age, per 10 year increase		0.61 (0.56 to 0.67)	<0.001	0.61 (0.48 to 0.78)	<0.001	0.62 (0.50 to 0.76)	<0.001	0.63 (0.55 to 0.71)	<0.001	0.57 (0.50 to 0.66)	<0.001	0.58 (0.44 to 0.76)	<0.001	0.57 (0.47 to 0.69)	<0.001	0.52 (0.43 to 0.64)	<0.001
Gender	Male	REF (1.00)		REF (1.00)		REF (1.00)		REF (1.00)		REF (1.00)		REF (1.00)		REF (1.00)		REF (1.00)	
	Female	1.26 (1.03 to 1.54)	0.03	0.90 (0.56 to 1.47)	0.68	1.34 (0.99 to 1.81)	0.06	1.27 (0.91 to 1.76)	0.16	1.35 (0.92 to 1.97)	0.12	1.31 (0.71 to 2.41)	0.38	1.32 (0.84 to 2.10)	0.23	1.66 (1.18 to 2.32)	0.003
Hospital admission	No	REF (1.00)		REF (1.00)		REF (1.00)		REF (1.00)		REF (1.00)		REF (1.00)		REF (1.00)		REF (1.00)	
for alcohol use	Yes (not recent)	1.04 (0.75 to 1.44)	0.84	1.53 (0.78 to 3.01)	0.22	1.29 (0.85 to 1.95)	0.23	1.16 (0.80 to 1.67)	0.44	1.21 (0.76 to 1.94)	0.42	0.76 (0.29 to 1.98)	0.57	1.29 (0.70 to 2.39)	0.42	0.73 (0.41 to 1.30)	0.29
	Yes (recent)	1.93 (1.41 to 2.63)	<0.001	2.39 (1.35 to 4.24)	0.003	1.45 (1.01 to 2.08)	0.04	1.72 (1.14 to 2.60)	0.01	1.53 (1.04 to 2.25)	0.03	1.56 (0.86 to 2.83)	0.15	1.61 (0.97 to 2.66)	0.07	1.64 (1.16 to 2.31)	0.005
Hospital admission	No	REF (1.00)		REF (1.00)		REF (1.00)		REF (1.00)		REF (1.00)		REF (1.00)		REF (1.00)		REF (1.00)	
for substance use	Yes (not recent)	2.24 (1.71 to 2.92)	<0.001	1.08 (0.56 to 2.07)	0.82	1.33 (0.85 to 2.09)	0.22	1.93 (1.37 to 2.72)	<0.001	1.58 (1.05 to 2.37)	0.03	1.44 (0.64 to 3.20)	0.38	2.01 (1.11 to 3.61)	0.02	1.20 (0.65 to 2.23)	0.56
	Yes (recent)	2.91 (2.16 to 3.91)	<0.001	1.51 (0.69 to 3.31)	0.31	1.71 (1.09 to 2.68)	0.02	2.88 (1.99 to 4.15)	<0.001	2.54 (1.69 to 3.82)	<0.001	1.94 (0.98 to 3.83)	0.06	1.74 (1.09 to 2.77)	0.02	1.74 (1.22 to 2.47)	0.002
Charlson comorbidity index		0.99 (0.98 to 0.99)*	<0.001	1.22 (1.06 to 1.39)	0.004	1.22 (1.14 to 1.31)	<0.001	1.24 (1.10 to 1.41)	0.001	1.36 (1.20 to 1.54)	<0.001	1.14 (0.92 to 1.40)	0.24	1.18 (1.05 to 1.32)	0.004	1.02 (0.91 to 1.15)	0.68

cells shaded in grey are statistically significant at P<0.05

* First degree fractional polynomial : power = -2

Interpretation:

The Standardised mortality ratio (SMR) is the ratio of observed to expected death, where expected deaths indicate the number of deaths that would have occurred if cured patients exhibited the same age/sex/year specific mortality rates as the corresponding general population.

The *SMR ratio* indicates the ratio of SMR in the group of interest relative to the reference group. For example, if the SMR ratio for individuals with a recent substance use hospital admission is 3.0, this means the SMR is three time higher in this group versus individuals with no previous admission.

Table S18: characteristics of cured hepatitis C patients from a previous population-based study in Scotland in the IFN-free DAA era versus the present analysis

	Previous population-based study (IFN-based era): Scotland*	Present study (IFN-free era): Scottish patients
Number of patients	1824	7691
Cirrhosis, with or without ESLD (%)	5.8	25.7
Age, mean	40.7	46.1
Male (%)	67.9	72.2
Past hospitalisation due to alcohol use (%)	6.9	20.6
Past hospitalisation due to drug use (%)	14.3	34.1
Crude mortality rate	7.1 deaths/1000 PYs	16.1 deaths/1000 PYs
Standardised mortality ratio	1.86 (95%CI:1.49-2.32)	4.53 (95%CI:4.10-5.00)

* Innes H, et al. J Hepatol. 2017;66:19-27.

APPENDIX A:

Data linkage- missing data perspective.

Scotland

Patient records held on the Scottish HCV clinical database were linked to mortality and hospital admission data. This linkage was performed using the Community Health Index (CHI) number, a unique identifier allocated to every patient in NHS Scotland. The CHI number is recorded for >95% of records in the Scottish HCV Clinical database. Thus, records from the HCV clinical database were linked deterministically to records in national health registries (e.g. SMR01 and the mortality register) via matching CHI number. All linkages were approved by the Public Health Scotland Privacy Benefit Panel (application number:156-0457).

On the latest version of the HCV Scottish clinical database (downloaded April 2021) there were 12,395 records of antiviral therapy resulting in a sustained viral response. Of these, only 72 did not have a valid CHI number recorded. These individuals have unknown mortality status therefore and were excluded from this study.

England

HCVRUK study participants with cirrhosis were linked to health registries held by NHS digital. Individuals were initially linked to the Patient Demographic Service (PDS) register using NHS number and date of birth. Record linkage data were only provided for patients who could be "traced" on the PDS.

Of 2277 individuals meeting our inclusion criteria for this study, 2157 (95%) individuals were traced on the PDS. The remaining 117 participants were excluded from the study. The main explanation for why 117 participants (5%) could not be traced on the PDS is likely to be data input errors for NHS number and date of birth, as these were entered manually into the HCVRUK database.

This linkage was approved by the Independent Group Advising on the Release of Data (IGARD), application number: NIC-72626.

British Columbia:

In BC, the Personal Health Number (a unique patient identifier in BC) for individuals with a HCV laboratory test are sent to ministry of health routinely.

At ministry of health, these personal health numbers are linked deterministically to numerous administrative health registers, including the mortality register.

The cohort for the present study was derived from Personal Health numbers sent to the Ministry of Health for routine record linkage. By definition therefore, all individuals in our cohort have a valid PHN; thus, there should be no missing information on mortality status or other health registry-derived data variables.

However, some HCV laboratory tests do no provide sufficient information for the personal health number to be established – thus, there is potential for some SVR patients to be excluded from our cohort.

For more information regarding the linkage process, see Janjua NZ et al. PMID: 26954020.

APPENDIX B: Lexis expansion:

Lexis expansion was performed to update background mortality risk as individuals age and progress through calendar years.

For example, suppose an individual was born on 1st May 1960, achieved HCV cure on 15th January 2015 and died on 15th January 2019. In this case, the total follow-up time would be four years.

Before lexis expansion, this individual would be analysed as a single row of data, with a background mortality rate corresponding to the patient's age group and calendar year at the time cure was achieved.

After lexis expansion, this single row of data is split into 6 records, each representing a unique combination of age group and calendar year (see Table below).

Note that due to aging, the number of expected deaths (calculating by multiplying the general population mortality rate by the duration of follow-up) for this hypothetical patient is higher after the lexis expansion (0.0287) versus before the lexis expansion (0.019).

Of note, in lexis expansion, single calendar years were used to split follow-up; i.e. 2014; 2015; 2016; 2017; 2018 and 2019.

The age group splits were: <20; 20-29; 30-39; 40-44; 45-49; 50-54; 55-59; 60-64;65-69; 70-74; 75-79; and 80+ years.

A more general explanation of lexis expansion can be found in: Essential medical statistics book, Kirkland and Sterne; Chapter 24.

Lexis Expansion: hypothetical example

Before lexis expansion.

ID number	DOB	age at cure	date of cure	date of death	time start, years	time stop, years	Mortality event	Follow-up duration, years	age group	calendar year	acquired age	GP mortality rate	Expected deaths
100	01/05/1960	54.7	15/01/2015	15/01/2019	0	4	Yes	4	50-54	2015	54.7	0.0048	0.019

After lexis expansion for age group and sex

ID number	DOB	age at cure	date of cure	date of death	time start, years	time stop, years	Mortality event	Follow-up duration, years	age group	calendar year	acquired age	GP mortality rate	Expected deaths
100	01/05/1960	54.7	15/01/2015	15/01/2019	54.71	55.00	No	0.29	50-54	2015	54.71	0.0048	0.001397
100	01/05/1960	54.7	15/01/2015	15/01/2019	55.00	55.67	No	0.67	55-59	2015	55.00	0.0074	0.004974
100	01/05/1960	54.7	15/01/2015	15/01/2019	55.67	56.67	No	1	55-59	2016	55.67	0.0076	0.007619
100	01/05/1960	54.7	15/01/2015	15/01/2019	56.67	57.67	No	1	55-59	2017	56.67	0.0071	0.007145
100	01/05/1960	54.7	15/01/2015	15/01/2019	57.67	58.67	No	1	55-59	2018	57.67	0.0073	0.007333
100	01/05/1960	54.7	15/01/2015	15/01/2019	58.67	58.71	Yes	0.04	55-59	2019	58.67	0.0068	0.000257
